# Design and Development of an Electronic Platform for Allergen Immunotherapy in China

**DOI:** 10.1155/ijta/5572974

**Published:** 2025-12-12

**Authors:** Wanjun Wang, Zheng Zhu, Wanyi Fu, Qiurong Hu, Xu Shi, Ruchong Chen, Jing Li

**Affiliations:** ^1^ Department of Allergy and Clinical Immunology, National Clinical Research Center for Respiratory Disease, State Key Laboratory of Respiratory Disease, Guangzhou Institute of Respiratory Health, The First Affiliated Hospital of Guangzhou Medical University, Guangzhou, China, gzhmc.edu.cn

## Abstract

**Background:**

A variety of patient populations receive allergen immunotherapy (AIT), but clinicians still lack an effective electronic platform to manage them.

**Methods:**

We designed a platform based on the framework of value set extraction standardization, integration, and structurization. Average medication scoring (AMS) and other electronic medical records were developed and stored on a MySQL database. Data storage is hosted on cloud servers, linked to a mobile application, Bluetooth lung function monitoring, and a dedicated website that acts as a front‐end.

**Results:**

Since 2015, 23,847 patients in 48 hospitals with AIT prescriptions were included. Of these patients, the median age was 15 (interquartile range, 10–27) years. Allergic rhinitis was the most common disorder and accounted for 9753 (40.9%) of the cases. Five hundred and thirty‐three basic data elements and six independent modules were constructed for the platform to facilitate the physicians in establishing SCIT projects, symptom scores, AMS, lung function tests, and follow‐up appointments. One hundred and twelve drugs including 10 dosage forms were identified from an internal list of the dataset. The unit score was calculated based on the action mechanism of the medicine. The AMS formula has six parameters: total dose, frequency, period, unit score, unit dose, and follow‐up days. A lower AMS suggests a better treatment efficacy.

**Conclusion:**

The program presented our experience in developing, pilot testing, and evaluating an electronic AIT platform, and future research would indicate whether the template could be made more time efficient in clinical practice.

## 1. Introduction

In the last few decades, there has been a progressive increase in the prevalence of allergic disease in China [1]. Methods for the prevention and treatment of allergic diseases include allergen avoidance, pharmacotherapy, allergen immunotherapy (AIT), and patient education, among which AIT is the only therapy that alters the natural immunological course of allergic diseases to achieve long‐term remission [2]. The proportion of patients in China who underwent allergen subcutaneous immunotherapy (SCIT) was approximately 4.5% [3]. However, there are still some problems associated with clinical practice. First, in AIT, in comparison to medication therapy, adverse reactions occur more frequently during the dose escalation period than in the maintenance period. In rare cases, early epinephrine administration is needed to improve outcomes and prevent progression to severe and fatal anaphylaxis. This requires a high level of compliance to achieve optimal efficacy in long‐term treatment [4]. Secondly, AIT has traditionally been documented through paper‐based forms so that searching and storing the accumulated paper records were time‐consuming and laborious in the continuous monitoring of the disease. Manual input lowers clinical efficiency and so negatively impacts upon disease progression, comorbidities, wellbeing, and prognosis [5]. Thirdly, as AIT is intended to improve disease control rather than replace pharmacological treatment altogether, most patients require concomitant medication [2]. The World Allergy Organization (WAO) has suggested that a score of 1 should be attributed to antihistamines, with a score of 2 for nasal corticosteroids and a score of 3 for oral corticosteroids [6]. These medication scores had been proposed to assess treatment efficacy in clinical trials but the scores should be refined and customized according to the patients′ regimen.

These problems reveal an urgent need for the development of an easy‐to‐use and effective AIT platform. Here, we propose a new mobile application to overcome these challenges by offering allergists and medical professionals a user‐friendly, time‐efficient, and accessible mobile‐based digital SCIT system. The system was tested prospectively in multicenter real‐world research in China.

## 2. Method

### 2.1. Overview

To design the electronic platform, we established a multidisciplinary task force composed of allergists, pharmacists, nurses, software engineers, and medical informatics experts from the First Affiliated Hospital of Guangzhou Medical University and Zhejiang e‐LinkCare Meditech Co. Ltd. The task force held a meeting every 2 weeks from Jan 1 to Dec 31, 2015, and was involved in every process from service planning to the development and evaluation of the system.

**Figure 1:**
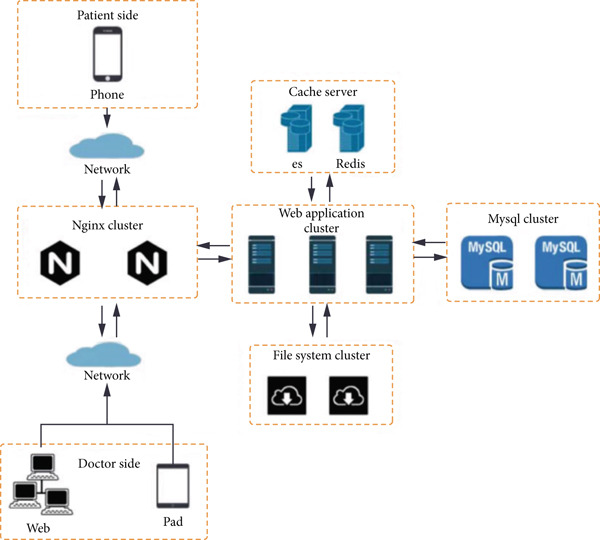
Information architecture of database linked to the AIT platform. ES: Elasticsearch data cache; Nginx: a kind of load equalization; Redis: a kind of data cache; SQL: structured query language.

The overall system design scheme comprises four procedures: (a) development of the allergy standard elements; (b) data coding, extraction, and integration in a structured query language (SQL) database based on Redis and Elasticsearch cache servers; (c) organization and refinement of each patient′s information storage architecture; and (d) visualization of modules on WeChat and iPads. An overview of the system architecture is shown in Figure 1.

### 2.2. Patient Privacy and Platform Security

The electronic platform procedure was carried out in accordance with the Declaration of Helsinki and was approved by the respective Institutional Review Boards (IRBs) of the First Affiliated Hospital of Guangzhou Medical University. Each data management department deidentified, formatted, and shared the data from the platform. Unless locally managed mapping data were exposed, no patients could be identified in particular. The system included user identification, password requirements, specific permissions for accessing data based on user roles, database security, and IP address restrictions. These restrictions maintained control over platform access and were configured according to the specific needs and policies of each hospital.

### 2.3. Statistical Analysis

We summarized data as numbers (*n*) and frequencies (%) if they are categorical, and as mean or median, and standard deviation or interquartile range if quantitative. We examined all described analyses for each of the study centers. All computations were performed with the statistical software IBM SPSS Version 25 (IBM Corporation).

## 3. Results

### 3.1. Establishment of SCIT Specific Standard Data Elements

This platform defines 533 terms to do with allergic rhinitis, asthma, and atopic dermatitis, including 210 common elements and 323 specific elements. The rhinitis dataset defines 178 fields. The asthma dataset defines 73 fields. The dermatitis dataset defines 149 fields. Common elements such as name, ID, age, gender, race, education, date of diagnosis, date of birth, height, weight, and vital signs and specific elements like allergen, wheal, skin prick test, provocation test, immunoglobulin E, induced sputum, exhaled nitric oxide, symptoms, signs, cumulative dose, peak flow, volume, and injection site are shown in Table 1.

**Table 1:** Standard elements of the SCIT platform.

**Module**	**No. of elements**	**Content**	**Data label**
Demography	45	Name, gender, ID number, telephone number, address, date of initial diagnosis, and date of birth, etc.	Essential information
		Height, weight, blood pressure, heart rate, allergy drug, date of initial injection, first diagnosed doctor, family history, smoking history, etc.	
Initial records	74	Clinical symptoms, confirmed time, frequency, daily physical activity, regimen and medication, the impact on daily life, etc.	
Allergen examination report	16	Report type, allergen wheal size, total IgE, specific IgE, challenge tests, blood routine, etc.	Laboratory test information
Lung function records	12	PEF, FEV_1_, FEV_1_% pred, FVC, FVC% pred, FEV_1_/FVC% pred, FEF 25, FEF 75, MMEF, etc.	
Payment record	6	Date, payment time, payment type, etc.	Expenditure information
Follow‐up note	206	Electronic questionnaire score (VAS, ACT, ACQ, RQLQ, AQLQ, EASI, and SCORAD), physical sign score, rash score, average medication score (total dose, frequency, period, unit score, unit dose, and follow‐up days.), etc.	Follow‐up information
Injection records	45	Ordering physician name, clinical site location, patient name, AIT indication, allergens per vial, extract lot numbers, vial expiration dates, injection administration date, extract concentration for each vial, etc.	
Nonattendance management	8	Treatment number, injection serial number, shedding time, reason of shedding, contact information, etc.	
Records′ export	54	Basic information, initial medical records, treatment records, allergen reports, records of lung function, and adverse reactions records, etc.	Technology information
Administration setting	16	Hospital name, department name, responsible account number, email address, daily injection and doctors′ opening time, stop time, follow‐up score setting, version description, etc.	
WeChat end	14	Patient application page, etc.	
	12	Follow‐up interface, mainly the delayed response and its management, etc.	
	27	System message, the next injection reminder, observation period reminder, etc.	

Abbreviations: ACQ, asthma control questionnaire; ACT, asthma control test; AQLQ, asthma quality of life questionnaire; EASI, eczema area and severity index; FEF, forced expiratory flow; FEV1, forced expiratory volume in 1 s; FVC, forced vital capacity; MMEF, maximal midexpiratory flow; PEF, peak expiratory flow; RQLQ, rhinoconjunctivitis quality of life questionnaire; RTSS, rhinoconjunctivitis total symptom score; SCORAD, scoring atopic dermatitis; VAS, visual analogue scale.

### 3.2. Establishment of Entity Diagram and SCIT Modules by Algorithm

Source code was written to represent the paper‐based text. Using predefined standard data elements, data extraction was performed using Extract‐Transform‐Load (ETL) techniques. Through ETL, data from multiple noninteroperable systems was consolidated into a common database. Then, using the unique identifier provided by the Electronic Patient Master Index (EPMI), all records for the same patient, from different systems, were integrated into a single record. All data processing up to this point was performed using Java, Objective‐C, Python, and Android. Details of the six modules of the system are shown in Table 2 and Appendix S1.

**Table 2:** Algorithms of the six modules on the platform.

**Modules**	**Content**	**No. of rules**	**Elements populated from structured data**	**Data type**	**Output code** ^**a**^ **examples**
I. Dashboard	Display an overview of the characteristics of patients including diagnosis history, allergen laboratory results, lung function test, treatment status, and reasons for stopping therapy	42	Dashboard.category Dashboard.subject Dashboard.encounter Dashboard.status Dashboard..dateAsserted	Reference	private String id;private String userName;private String realName;private String phone; private String password;private long createTime;private int userPermission

II. Lung function measurement	This module includes the real‐time transmission of flow–time curve, flow–volume curve, time–volume curve to the PC end by Bluetooth	26	Lung function measurement.category Lung function measurement.subject Lung function measurement.encounter Lung function measurement.clinicalstatus	CodeableConcept	newRecord.healthStatus = healthStatus;newRecord.beforePEF = beforePEF;newRecord.beforePEFTime = beforePEFTime;newRecord.afterPEF = afterPEF;newRecord.afterPEFTime = afterPEFTime

III. Immunotherapy	This is the basis of the template, including date of injection, number of vials, cumulative dose, site of injection, record of adverse reactions, and treatment	35	Immunotherapy.category Immunotherapy.subject Immunotherapy.encounter Immunotherapy.status Immunotherapy.patient Immunotherapy.date	CodeableConcept	Paragraph titleNumber = new Paragraph (“serial number,”chineseFont);table.addCell (getContentCenterPdfCell(titleNumber));Paragraph titleName = new Paragraph(“full name,”chineseFont)

IV. AMS template	This module is designed for users to evaluate the symptom alleviation and medication scores according to the timeline of immunotherapy	39	Averagemedicationscore template.category Averagemedicationscore template.subject Averagemedicationscore template.encounter Averagemedicationscore template.status Averagemedicationscore template.patient Averagemedicationscore template.date	Boolean and integer	CREATE TABLE “pfm2_followup_medicalrecord” (“followup_id” int(11) NOT NULL COMMENT “followup_id,”“drug_id” int(11) DEFAULT NULL COMMENT; “drug_type” int(4) DEFAULT NULL COMMENT; “drug_name” varchar(50)

V. Department project	This module is designed for users to count the workload in healthcare, statistical summary, follow up the suboptimal compliance patients, and data export	22	Department project.category Department project.subject Department project.encounter Department project.status Department project.patient Department project.dataAbsentReason	Code	private String patientId;private String followUpId;private long followUpTime;private String asthmaSympton;private long asthmaStartTime;private long asthmaEndTime;private String rhinitisSympton

VI. Application	This module is designed for reminder through mobile messaging application, allergy science popularization as well as communication between allergists and patients about delayed allergic reaction of AIT	18	Application.dateTime Application..subject Application..encounter Application..status Application..patient	Code	public synchronized void connect(BluetoothDevice device) {isConnecting = true;mInstrumentSN = null;mInstrumentState = null;startTimeoutTimer();mBtDevice = device;mBufferedCurveSevlet = null;Intentintent = new Intent(mContext, BluetoothLeService.class)

Abbreviations: AIT, allergen immunotherapy; AMS, average medication score.

^a^For expansions of abbreviations used and the whole output code, please refer to Appendix S1.

**Figure 2:**
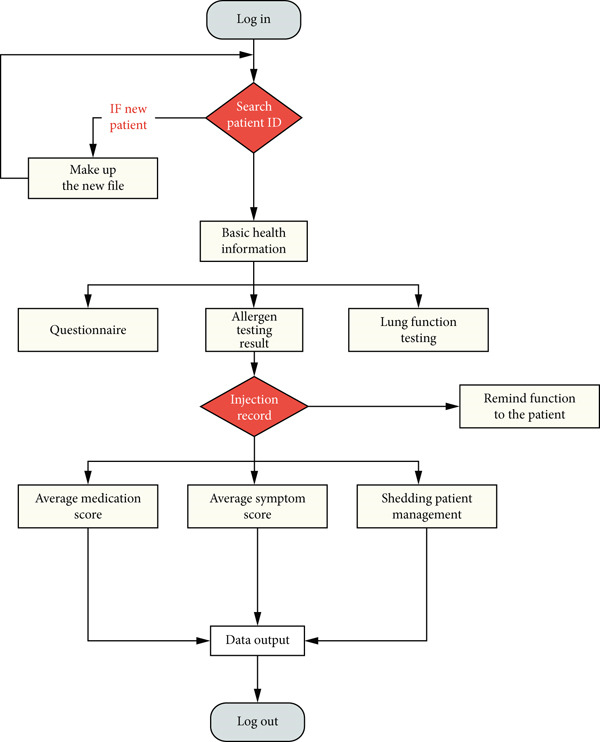
Navigation diagram for the data processing.

In the operational logic diagram, we organized the various functional modules and messy input data according to the clinical diagnosis and treatment process. For new patients, a new unique identifier needed to be created. For existing patients, physicians could input the patient identifier and the system would automatically retrieve the patient′s information that was stored on the platform. The process overview is shown in Figure 2.

### 3.3. Overview of the AIT Platform

From January 2015 to December 2023, the details of 23,847 patients with allergic diseases undergoing SCIT from 48 hospitals nationwide were stored on the platform. These hospitals had jointly signed a strategic cooperation framework agreement to form an alliance on AIT. Among these patients, 14,871 (62.4%) were male, and 8976 (37.6%) were female. The median age was 15 years (IQR, 10–27 years). The number of immunotherapy treatments was 559,926, with 157,519 follow‐up visits and 1,081,395 pulmonary function tests. All the platform data are updated in real time and transmitted to the cloud server daily. The demographics data are shown in Table 3.

**Table 3:** Demographic data—Clinical data from the 48 hospitals across China.

**Characteristics of the eligible population**	**N**	**%**
Gender		
Male	14,871	62.4
Female	8976	37.6
Total	23,847	
Hospital distribution		
Northern China	7	14.6
Eastern China	25	52.1
Central China	11	22.9
Southern coast	5	10.4
Total	48	
Year distribution		
2015	490	2.1
2016	723	3.0
2017	961	4.0
2018	2120	8.9
2019	2899	12.2
2020	3564	14.9
2021	4492	18.8
2022	3864	16.2
2023	3849	16.1
Age distribution		
3–5 years	2683	11.3
6–17 years	14,186	59.5
18–40 years	5337	22.4
41–64 years	1526	6.4
≥ 65 years	115	0.5
Median age (interquartile range)	15 (10–27)	
Disease distribution		
AR	9753	40.9
AS	4459	18.7
AR + AS	3649	15.3
AS + AD	1407	5.9
AR + AD	2003	8.4
AR + AS + AD	1812	7.6
Other	763	3.2
SCIT recordings	559,926	—
Recordings at baseline and follow‐up	157,519	—
Lung function recordings	1,081,395	—

*Note:* Percentages may not total 100 due to rounding.

Abbreviations: AD, atopic dermatitis; AR, allergic rhinitis; AS, asthma; SCIT, subcutaneous immunotherapy.

### 3.4. Interface Function of the PC End and WeChat End

The medical PC end includes (1) SCIT personal profile binding, including basic medical records, allergen information, and pulmonary function; (2) follow‐up records, including symptom score, medication score, pulmonary function measurements, and immunotherapy records (Figures 3a, 3b, 3c, 3d, and 3e); (3) patient injection list and dropout management; and (4) other functions, including security management and export of data.

Figure 3Screenshot showing electronic platform from one patient. (a) Immunotherapy screen. (b) Medication detail screen. (c) AMS screen. (d) Atopic dermatitis severity combined with AMS screen. (e) Symptom score screen. (f) WeChat end screen. The text was translated from the Chinese version by the first author. AMS: average medication score.

**Figure:**
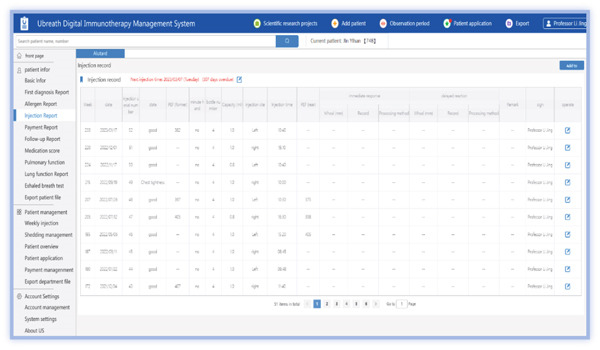
(a)

**Figure:**
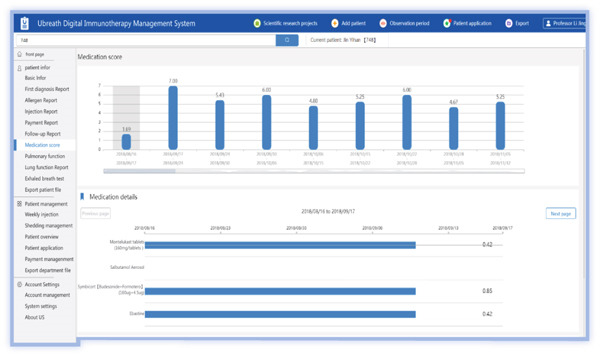
(b)

**Figure:**
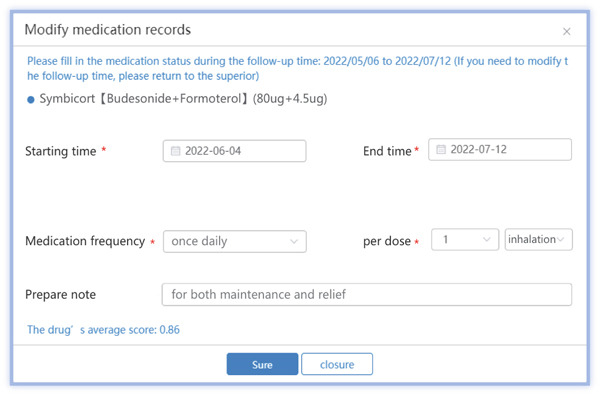
(c)

**Figure:**
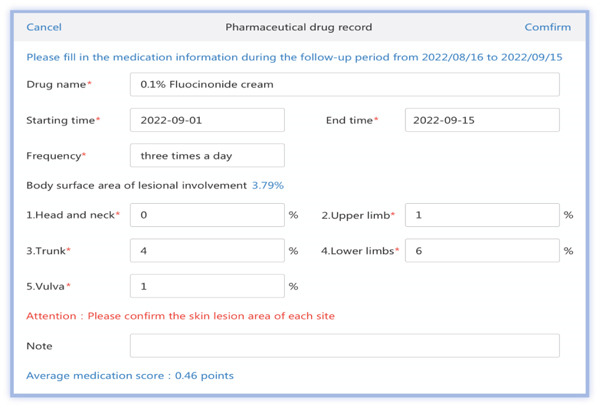
(d)

**Figure:**
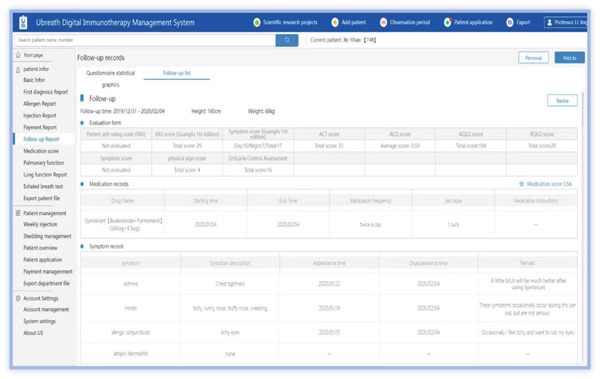
(e)

**Figure:**
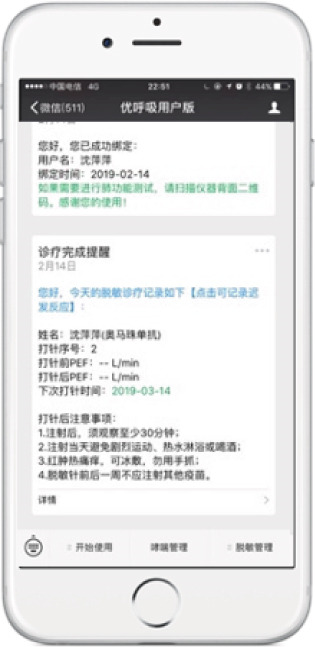
(f)

On the patient WeChat end, the platform sends messages to the patients through the application, including lung function results during AIT, and requests for patients to educate themselves in the application module or complete electronic questionnaires (Figure 3f).

### 3.5. Developing Medication Scoring Calculation for SCIT

There are 112 drugs in the medication dataset including 24 for rhinitis, 51 for asthma, and 37 for dermatitis. We defined a medication scoring policy to facilitate medical staff′s understanding of the overall medication status of patients during SCIT. The formula for the average medication score (AMS) is defined as follows:

Average medication score=total dose×frequency×period/unit dose×unit scorefollow‐up days

The unit scores of various commonly used drugs are formulated according to their pharmacological mechanism and dosage–effect relationship as shown in Table 4. The formula takes into account the days and doses of patients in the follow‐up cycle. According to the formula, the more drugs there are, the higher the dose, and the longer the days of use, the higher the AMS value is.

**Table 4:** Medication unit score policy of the electronic platform.

**Medication statement**	**Administration method**	**Medication list (** **N** **)**	**Unit score ranking**	**Examples**
Drainage and clearance	Irrigation	5	0.1	Saline solution
Short‐acting bronchodilation, topical anti‐inflammatory and topical antihistamine, ophthalmic and cutaneous corticoids cream	Inhale/spray/nebulization/ointments	3	0.5	Mometasone furoate cream
		1	0.625	Budesonide (100 *μ*g)
		35	1	Flixotide (125 *μ*g)
Low‐dose bronchodilation combined with inhaled corticosteroids	Inhale	4	1.5	Symbicort (80/4.5 *μ*g)
Short‐acting anticholinergic and beta2‐agonist, systematic antihistamine, relieve cough, phlegm‐eliminating, reduced edema and congestion	Inhale/oral/nebulization	40	2	Clarityne (10 mg) Seretide (250/50 *μ*g)
Leukotriene receptor antagonist	Oral	3	2.5–3.5	Montelukast (10 mg)
Long‐acting bronchodilation combined with inhaled corticosteroids	Inhale	4	4–5	Symbicort (320/9 *μ*g)
Long‐acting bronchodilation combined with inhaled corticosteroids and systemic antihistamine	Inhale/intramuscular injection	3	6	Seretide (500/50 *μ*g) Diphenhydramine (20 mg)
Nebulized inhaled corticosteroids	Nebulization	1	10	Budesonide suspension (1 mg)
Oral corticosteroids	Oral	4	20–25	Prednisone (5 mg)
Immunosuppression	Oral	2	40	Methotrexate (2.5 mg)
Intravenously bronchodilation	Intravenous injection	1	80	Theophylline (0.2 g)
Biologics	Subcutaneous injection	3	100	Omalizumab (150 mg)
Intravenously corticosteroids	Intravenous injection	1	150	Dexamethasone (5 mg)
Intravenously corticosteroids	Intravenous injection	1	250	Methylprednisolone (40 mg)
Antianaphylaxis	Intramuscular injection	1	1000	Adrenaline (1 mg)
Total		112		

Based on the SCORAD index [7], the skin lesion area is calculated as a percentage of the patient′s palm size to the body surface area, with 1% being the standard. In the medication scoring formula mentioned above, an additional coefficient is added based on the lesion area of dermatitis. The formula is as follows:

Average medication score=total dose×frequency×period/unit dose×unit scorefollow‐up days×skin lesion area

According to the formula, the higher the dose and the longer the days of use, the larger the skin lesion area is, and the higher the dermatitis AMS is. As shown in Figure 3c,d, clinicians can adjust weights via user interfaces based on local guidelines. Integrate with EHRs to autocalculate scores using existing medication data, which is then displayed on the screen, allowing a comparison over time. Adding the score of each drug gives the total AMS of the patient as shown in Figure 3c,d.

### 3.6. Platform‐Based Operational Flow of SCIT

**Figure 4:**
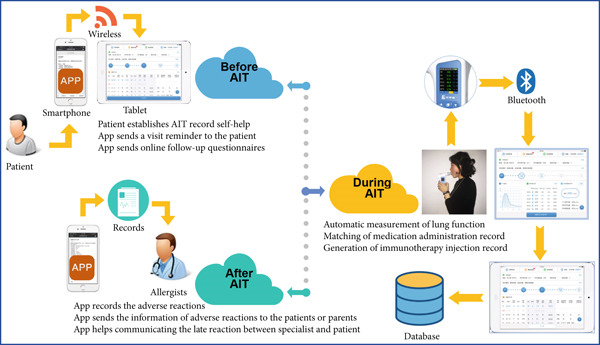
Workflow for the electronic platform. AIT: allergen immunotherapy.

The operational process is as follows: new patients use the app to create an ID number. The system will subsequently send messages for questionnaires. During SCIT, portable pulmonary function monitoring results are uploaded to the platform via Bluetooth for evaluation. The platform automatically matches the patient′s medication, injection, and adverse reaction records, which are also uploaded to the cloud database. After SCIT, the system also sends details of the next appointment to the patient. If any delayed adverse reactions occur after the patient leaves the clinic, they can contact the allergist and communicate online. The workflow is summarized in Figure 4.

### 3.7. Secure Role‐Based Clinical Research System With Dynamic Access Control

Users need an account and password to access the system. A roles‐and‐rights concept has been established to configure access to each study of the system separately. We created the main roles: allergy center physician (Level 1, Level 2)/attending physician, nurse, simple user (e.g., student), and admin. The roles of a user can change dynamically and are study‐dependent, that is, being a leading physician (Level 1) for one study and for another a standard user with only reading rights. Communication with external users from participating study centers is realized by an intermediate application gateway which receives https requests, checks them, and sends only valid requests to the server in the intranet of the hospital.

## 4. Discussion

In this study, we developed an intelligent platform for SCIT clinical practice, in which data on routine healthcare in AIT were successfully integrated, structured, and normalized. Our intelligent platform supports six modules that enable the allergist to identify eligible patients, establish SCIT projects, track the follow‐up by using AMS, monitor lung function tests and appointments, and share the data between health centers.

Internationally, electronic records, social media, and mobile applications have been applied in clinical practice and patient education of allergic diseases [8, 9]. Jariwala et al. demonstrated the clinical feasibility of an AIT system based on electronic records [10]. However, there is no published research about AIT using mobile technology in China. Therefore, this pilot study creates a domestic precedent. Much of the administrative work, previously done manually, is now being replaced by electronic platforms. Electronic spirometers improved patients′ perception of treatment efficacy, especially during the pandemic [11]. For example, patients can scan the QR code during pulmonary function tests to independently upload data through Bluetooth, instead of manual recording. Previous studies have shown that most applications and WeChat systems have a positive impact on users in terms of usefulness, acceptability, and ease of use. They provide a new way for patients to communicate with doctors and significantly improve the doctor–patient relationship [12, 13]. Our application can accurately transmit information about adverse reactions to patients or their parents and assist in effective communication between patients and doctors regarding late‐onset adverse reactions after leaving the clinic. The application can also provide access to information on various allergic diseases, such as articles, slides, and video clips on allergic disorder topics. The increasing number of records poses greater time and manpower costs of management [14]. Hence, an electronic platform can also improve the efficiency of AIT. First, compared to traditional manual paper records, a digital system makes data storage and retrieval more convenient. Doctors and nurses can focus on diagnosis and treatment giving them more time to allocate resources efficiently [15]. Second, symptom and medication scoring comparisons can detect a change over time in the SCIT responsiveness of patients. Third, utilizing the platform for managing the entire AIT process makes it less likely that treatment steps are missed. A previous study demonstrated that digital allergology can be applied in the context of broader clinical decision support systems for enhancing allergy‐related decisions and actions with pertinent, organized, clinical knowledge and patient information to improve allergy care and AIT prescriptions [16].

The drug scoring criteria in international desensitization clinical trials are relatively limited, only categorizing the use of emergency medications, antihistamines, and steroids into scores ranging from 0 to 3 [17]. This does not consider important parameters such as dosage form, duration of medication use, and follow‐up time and does not assign specific scores for each drug. Moreover, the international standards were originally designed for clinical trials and may not be suitable for other purposes [18, 19]. Our AMS algorithm accounts for distinct disease pathways (rhinitis, asthma, and dermatitis) and incorporates condition‐specific variables (e.g., lesion area for dermatitis). This modular design allows adaptation to allergic conditions by adding relevant coefficients, which can strengthen the adaptability. It defines specific scores based on the pharmacological mechanisms, half‐lives, and dose equivalence. For instance, 80 μg of budesonide is defined as 0.5 points, while 100 μg is defined as 0.625 points. Formoterol is defined as 1 point, while Symbicort (80/4.5) per inhalation is equivalent to 1.5 points and Symbicort (160/4.5) is equivalent to 2 points and so on. Symbicort (320/9) is equivalent to 4 points, while Seretide (500/50 μg) per inhalation is 6 points, and oral methylprednisolone (4 mg/tablet) is 25 points. Adrenaline is often used in severe systemic allergic reactions; it is assigned a weight where 1 mg is equivalent to 1000 points. The use of theophylline is common in China but not in other countries. Therefore, we add region‐specific drugs (e.g., theophylline in China) without disrupting the core formula. The formula integrates duration of use and dose intensity, capturing temporal aspects of medication burden. This accommodates varied follow‐up schedules (e.g., monthly vs. quarterly) and fluctuating disease severity (e.g., seasonal exacerbations).

There are some limitations of the platform. First, the platform cannot replace the face‐to‐face diagnosis and the interpersonal relationship between doctors and patients [20]. Second, cultural acceptance of digital tools (e.g., in elderly populations) and regulatory harmonization (e.g., data privacy laws) remain critical barriers [21, 22]. The solution to the problem might be that traditional healthcare must be retained simultaneously for this part of patients, with both ways in parallel and complementary. Third, in low‐income regions, limited access to diagnostics or drugs may reduce scoring accuracy. Weight‐based dosing in children or polypharmacy in elders may demand age‐adjusted algorithms of the AMS. Finally, drug metabolism (e.g., corticosteroid sensitivity) may differ across populations, potentially necessitating adjusted weights. Patients with overlapping conditions may require integrated scoring beyond allergy‐specific metrics. Thus, it needs continuous refinement via real‐world data. In the future, artificial intelligence (AI) technology is used to build a personalized prediction model, generate an accurate treatment plan suitable for patients′ characteristics, and realize closed‐loop early warning and intervention of adverse events such as adverse drug reactions and treatment risks in AIT.

In summary, the Chinese AIT digital management platform provides an integrated data management system that enables multicenter data sharing and reduces the difficulty of aggregating and analyzing data at the national level. Further studies on a larger scale may indicate whether the electronic platform can be made more time‐efficient in the setting of AIT without sacrificing safety, physician and patient satisfaction, and clinical effectiveness. The AMS algorithm is a robust, customizable tool with high potential for adaptation across contexts. Test the algorithm in diverse populations to refine weights and thresholds and correlate the AMS to clinical endpoints (e.g., symptom control and QoL) to ensure scores reflect real‐world efficacy.

## Conflicts of Interest

The authors declare no conflicts of interest.

## Author Contributions

Wanjun Wang, Zheng Zhu, Wanyi Fu, Qiurong Hu, Xu Shi and Ruchong Chen contributed equally to the study.

## Funding

The study is supported by the National Natural Science Foundation of China, 10.13039/501100001809 (82161138020), the Major Project of Guangzhou National Laboratory (GZNL2024A02002), and the Guangdong Innovation Team Project of General College and University (2023KCXTD024).

## Supporting information


**Supporting Information** Additional supporting information can be found online in the Supporting Information section. Appendix S1. The output code of the digital platform for allergen immunotherapy. The supporting information is the whole output code for six modules on the AIT platform. The codebase enables healthcare data management, patient monitoring, and telehealth services. Built with modern frameworks, it integrates EHR/EMR systems and wearable device APIs.

## Data Availability

To protect study participant privacy, the data and materials that support the findings of this study are available from the corresponding author upon reasonable request.

## References

[1] Wang W. , Wang J. , Song G. , Xie H. , Lin X. , Chai R. , Zhu R. , He Y. , Tang J. , Wang J. , Yang J. , Zhi L. , Wu L. , Jiang Y. , Zhou X. , Huang D. , Wang N. , Xu R. , Gao Y. , Chen Z. , Liu J. , Han X. , Tan G. , Wu J. , Zhao D. , Chen J. , Zhang X. , Li M. , Sun Y. , Jiang Y. , Zhang W. , Qiu Q. , Liu C. , Yin J. , Hao G. , Li H. , Xu Y. , Chen S. , Zhang H. , Chen S. , Meng J. , Zeng D. , Tang W. , Hao C. , Li J. , Zhong N. , and for the China Alliance of Research on Respiratory Allergic Disease , Environmental and sensitization variations among asthma and/or rhinitis patients between 2008 and 2018 in China, Clinical and Translational Allergy. (2022) 12, no. 2, e12116, 10.1002/clt2.12116, 35136540. 35136540 PMC8809046

[2] Bousquet J. , Lockey R. F. , and Malling H. J. , Scientific Criteria and the Selection of Allergenic Foods for Product Labelling, Allergy. (1998) 53, no. 47 Supplement, 3–21, 10.1016/S0091-6749(98)70271-4, 10100969. 10100969

[3] Zheng M. , Wang X. , Wang M. , She W. , Cheng L. , Lu M. , Xing Z. , Ma F. , Zhu L. , Chen L. , Lin X. , Jiang X. , Zhu D. , Xu G. , Wen W. , Kong W. , Chen J. , Tao Z. , Xu Y. , Wang D. , Liu S. , Wang S. , Jiang W. , Sun J. , Zhao C. , Suo L. , Zhang H. , and Zhang L. , Clinical Characteristics of Allergic Rhinitis Patients in 13 Metropolitan Cities of China, Allergy. (2021) 76, no. 2, 577–581, 10.1111/all.14561, 33460167. 33460167

[4] Feng M. , Zeng X. , and Li J. , House Dust Mite Subcutaneous Immunotherapy in Chinese Patients With Allergic Asthma and Rhinitis, Journal of Thoracic Disease. (2019) 11, no. 8, 3616–3625, 10.21037/jtd.2019.06.35, 2-s2.0-85073331987, 31559069. 31559069 PMC6753424

[5] Seidman M. D. , Gurgel R. K. , Lin S. Y. , Schwartz S. R. , Baroody F. M. , Bonner J. R. , Dawson D. E. , Dykewicz M. S. , Hackell J. M. , Han J. K. , Ishman S. L. , Krouse H. J. , Malekzadeh S. , Mims J. W. , Omole F. S. , Reddy W. D. , Wallace D. V. , Walsh S. A. , Warren B. E. , Wilson M. N. , and Nnacheta L. C. , Clinical Practice Guideline: Allergic Rhinitis, Otolaryngology–Head and Neck Surgery. (2015) 152, no. S1, 10.1177/0194599814561600, 2-s2.0-84947491709. 25644617

[6] Canonica G. W. , Baena-Cagnani C. E. , Bousquet J. , Bousquet P. J. , Lockey R. F. , Malling H. J. , Passalacqua G. , Potter P. , and Valovirta E. , Recommendations for Standardization of Clinical Trials With Allergen Specific Immunotherapy for Respiratory Allergy. A Statement of a World Allergy Organization (WAO) Taskforce, Allergy. (2007) 62, no. 3, 317–324, 10.1111/j.1398-9995.2006.01312.x, 2-s2.0-33846964159, 17298350. 17298350

[7] Severity Scoring of Atopic Dermatitis: The SCORAD Index. Consensus Report of the European Task Force on Atopic Dermatitis, Dermatology. (1993) 186, no. 4, 23–31. 8435513 10.1159/000247298

[8] Matricardi P. M. , Dramburg S. , Alvarez-Perea A. , Antolín-Amérigo D. , Apfelbacher C. , Atanaskovic-Markovic M. , Berger U. , Blaiss M. S. , Blank S. , Boni E. , Bonini M. , Bousquet J. , Brockow K. , Buters J. , Cardona V. , Caubet J. C. , Cavkaytar Ö. , Elliott T. , Esteban-Gorgojo I. , Fonseca J. A. , Gardner J. , Gevaert P. , Ghiordanescu I. , Hellings P. , Hoffmann-Sommergruber K. , Fusun Kalpaklioglu A. , Marmouz F. , Meijide Calderón Á. , Mösges R. , Nakonechna A. , Ollert M. , Oteros J. , Pajno G. , Panaitescu C. , Perez-Formigo D. , Pfaar O. , Pitsios C. , Rudenko M. , Ryan D. , Sánchez-García S. , Shih J. , Tripodi S. , Van der Poel L. A. , van Os-Medendorp H. , Varricchi G. , Wittmann J. , Worm M. , and Agache I. , The Role of Mobile Health Technologies in Allergy Care: An EAACI Position Paper, Allergy. (2020) 75, no. 2, 259–272, 10.1111/all.13953, 2-s2.0-85073823066, 31230373. 31230373

[9] Bousquet J. , Caimmi D. P. , Bedbrook A. , Bewick M. , Hellings P. W. , Devillier P. , Arnavielhe S. , Bachert C. , Bergmann K. C. , Canonica G. W. , Chavannes N. H. , Cruz A. A. , Dahl R. , Demoly P. , De Vries G. , Mathieu-Dupas E. , Finkwagner A. , Fonseca J. , Guldemond N. , Haahtela T. , Hellqvist-Dahl B. , Just J. , Keil T. , Klimek L. , Kowalski M. L. , Kuitunen M. , Kuna P. , Kvedariene V. , Laune D. , Pereira A. M. , Carreiro-Martins P. , Melén E. , Morais-Almeida M. , Mullol J. , Muraro A. , Murray R. , Nogueira-Silva L. , Papadopoulos N. G. , Passalacqua G. , Portejoie F. , Price D. , Ryan D. , Samolinski B. , Sheikh A. , Siroux V. , Spranger O. , Todo Bom A. , Tomazic P. V. , Valero A. , Valovirta E. , Valiulis A. , VandenPlas O. , van der Meulen S. , van Eerd M. , Wickman M. , and Zuberbier T. , Pilot Study of Mobile Phone Technology in Allergic Rhinitis in European Countries: The MASK-Rhinitis Study, Allergy. (2017) 72, no. 6, 857–865, 10.1111/all.13125, 2-s2.0-85013460536, 28072463. 28072463

[10] Jariwala S. P. , Keskin T. , Achar K. , Henner J. , Talla G. , Rosenstreich D. L. , and Santana C. , Developing and Pilot Testing an Electronic Medical Record-Based Allergen Immunotherapy Template, Annals of Allergy, Asthma & Immunology. (2016) 117, no. 2, 206–208, 10.1016/j.anai.2016.06.011, 2-s2.0-84977615774, 27353025. 27353025

[11] Poowuttikul P. and Seth D. , New Concepts and Technological Resources in Patient Education and Asthma Self-Management, Clinical Reviews in Allergy and Immunology. (2020) 59, no. 1, 19–37, 10.1007/s12016-020-08782-w, 32215784. 32215784

[12] Haze K. A. and Lynaugh J. , Building Patient Relationships: A Smartphone Application Supporting Communication Between Teenagers With Asthma and the RN Care Coordinator, Computers, Informatics, Nursing. (2013) 31, no. 6, 266–271, 10.1097/NXN.0b013e318295e5ba, 2-s2.0-84880061800. 23728445

[13] Pizzulli A. , Perna S. , Florack J. , Pizzulli A. , Giordani P. , Tripodi S. , Pelosi S. , and Matricardi P. M. , The Impact of Telemonitoring on Adherence to Nasal Corticosteroid Treatment in Children With Seasonal Allergic Rhinoconjunctivitis, Clinical and Experimental Allergy. (2014) 44, no. 10, 1246–1254, 10.1111/cea.12386, 2-s2.0-84927520830, 25109375. 25109375

[14] Tripodi S. , Giannone A. , Sfika I. , Pelosi S. , Dramburg S. , Bianchi A. , Pizzulli A. , Florack J. , Villella V. , Potapova E. , and Matricardi P. M. , Digital Technologies for an Improved Management of Respiratory Allergic Diseases: 10 Years of Clinical Studies Using an Online Platform for Patients and Physicians, Italian Journal of Pediatrics. (2020) 46, no. 1, 10.1186/s13052-020-00870-z, 32711557. PMC7382563 32711557

[15] Shen Z. Y. , Wang F. , Tan G. L. , Yan T. , and Li R. , Effect of Mobile Platform in Improving Compliance and Efficacy of Subcutaneous Immunotherapy in Children With Allergic Rhinitis, Lin Chuang Er bi Yan Hou Tou Jing Wai Ke Za Zhi = Journal of Clinical Otorhinolaryngology Head and Neck Surgery. (2020) 34, no. 1, 28–32, 10.13201/j.issn.1001-1781.2020.01.007, 32086893. 32086893 PMC10128573

[16] Matricardi P. M. , Potapova E. , Forchert L. , Dramburg S. , and Tripodi S. , Digital Allergology: Towards a Clinical Decision Support System for Allergen Immunotherapy, Pediatric Allergy and Immunology. (2020) 31, no. S24, 61–64, 10.1111/pai.13165, 32017213. 32017213

[17] Clark J. and Schall R. , Assessment of Combined Symptom and Medication Scores for Rhinoconjunctivitis Immunotherapy Clinical Trials, Allergy. (2007) 62, no. 9, 1023–1028, 10.1111/j.1398-9995.2007.01469.x, 2-s2.0-34547665695. 17686105

[18] Caballero R. , Grau A. , Javaloyes G. , Del Pozo S. , León M. Á. , Romero M. , and Casanovas M. , Combination of Allergic Asthma Symptom and Medication Scores in Allergen Immunotherapy Trials: A Proposal, International Archives of Allergy and Immunology. (2021) 182, no. 7, 571–573, 10.1159/000513543, 33498057. 33498057

[19] Grouin J. M. , Vicaut E. , Jean-Alphonse S. , Demoly P. , Wahn U. , Didier A. , de Beaumont O. , Montagut A. , Le Gall M. , and Devillier P. , The Average Adjusted Symptom Score, a New Primary Efficacy End-Point for Specific Allergen Immunotherapy Trials, Clinical and Experimental Allergy. (2011) 41, no. 9, 1282–1288, 10.1111/j.1365-2222.2011.03700.x, 2-s2.0-79955375673, 21375606. 21375606

[20] Licskai C. , Sands T. W. , and Ferrone M. , Development and Pilot Testing of a Mobile Health Solution for Asthma Self-Management: Asthma Action Plan Smartphone Application Pilot Study, Canadian Respiratory Journal. (2013) 20, no. 4, 301–306, 10.1155/2013/906710, 2-s2.0-84882428529, 23936890. 23936890 PMC3956342

[21] Mai J.-E. , Three Models of Privacy: New Perspectives on Informational Privacy, Nordicom Review. (2016) 37, no. s1, 171–175, 10.1515/nor-2016-0031, 2-s2.0-85006751362.

[22] Prabhakaran L. , Chee W. Y. , Chua K. C. , Abisheganaden J. , and Wong W. M. , The Use of Text Messaging to Improve Asthma Control: a Pilot Study Using the Mobile Phone Short Messaging Service (SMS), Journal of Telemedicine and Telecare. (2010) 16, no. 5, 286–290, 10.1258/jtt.2010.090809, 2-s2.0-77954872520, 20576744. 20576744

