# Supplementary material for: Design and Development of an Electronic Platform for Allergen Immunotherapy in China
Source: Int J Telemed Appl. 2025 Dec 12;2025:5572974. doi: 10.1155/ijta/5572974 (PMC12752872; doi:10.1155/ijta/5572974)
Supplement: Supplementary file 1 — Supporting Information Additional supporting information can be found online in the Supporting Information section. Appendix S1. The output code of the digital platform for allergen immunotherapy. The supporting information is the whole output code for six modules on the AIT platform. The codebase enables healthcare data management, patient monitoring, and telehealth services. Built with modern frameworks, it integrates EHR/EMR systems and wearable device APIs. [file IJTA-2025-5572974-s001.docx]

Supplemental file: This is the whole output code for six modules on the AIT platform.

Digital health management system source code


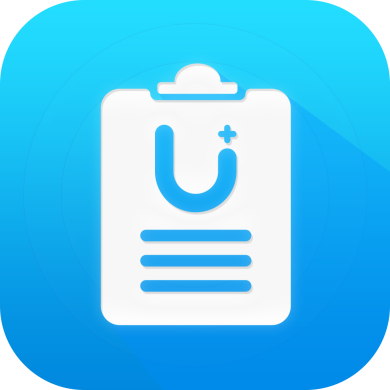


**package** com.elinkcare.ubreath.desensitization.account.accountinfo;

**import** android.os.Bundle;

**import** android.support.annotation.Nullable;

**import** android.support.v4.app.Fragment;

**import** android.support.v4.app.FragmentManager;

**import** android.support.v4.app.FragmentStatePagerAdapter;

**import** android.view.LayoutInflater;

**import** android.view.View;

**import** android.view.ViewGroup;

**import** com.elinkcare.ubreath.desensitization.R;

**import** com.elinkcare.ubreath.desensitization.widget.FragmentNavigationViewPager;

*/***

** Created by ${Ray} on 2017/3/27.*

**/*

**public class** AccountFragment **extends** Fragment{

**private** View **mView**;

**private** FragmentNavigationViewPager **mViewPager**;

**private** FragmentPagerAdapter **mPagerAdapter**;

**private** AccountInfoFragment **mAccountInfoFragment**;

**private** ChildAccountFragment **mChildAccountFragment**;

@Nullable

@Override

**public** View onCreateView(LayoutInflater inflater, @Nullable ViewGroup container, @Nullable Bundle savedInstanceState) {

**mView** = inflater.inflate(R.layout.***fragment_account*** , container, **false**);

initFragment();

initView();

initListener();

**return mView**;

}

**private void** initView(){

**mViewPager** = (FragmentNavigationViewPager) **mView**.findViewById(R.id.***vp_account***);

**mViewPager**.setScrollble(**false**);

**mPagerAdapter** = **new** FragmentPagerAdapter(getChildFragmentManager());

**mViewPager**.setAdapter(**mPagerAdapter**);

}

**private void** initFragment(){

**mAccountInfoFragment** = **new** AccountInfoFragment();

**mChildAccountFragment** = **new** ChildAccountFragment();

}

**private void** initListener(){

**mAccountInfoFragment**.setChildAccountManageClickListener(**new** View.OnClickListener() {

@Override

**public void** onClick(View v) {

**mViewPager**.setCurrentItem(1);

}

});

**mChildAccountFragment**.setOnBackClickListener(**new** View.OnClickListener() {

@Override

**public void** onClick(View v) {

**mViewPager**.setCurrentItem(0);

}

});

}

**private class** FragmentPagerAdapter **extends** FragmentStatePagerAdapter{

**public** FragmentPagerAdapter(FragmentManager fm) {

**super**(fm);

}

@Override

**public** Fragment getItem(**int** position) {

**switch** (position){

**case** 0:

**return mAccountInfoFragment**;

**case** 1:

**return mChildAccountFragment**;

**default**:

**return null**; *// never come here;*

}

}

@Override

**public int** getCount() {

**return** 2;

}

}

}**package** com.elinkcare.ubreath.desensitization.account.accountinfo;

**import** android.os.Bundle;

**import** android.support.v4.app.Fragment;

**import** android.util.Log;

**import** android.view.LayoutInflater;

**import** android.view.View;

**import** android.view.ViewGroup;

**import** android.widget.Button;

**import** android.widget.ProgressBar;

**import** android.widget.TextView;

**import** android.widget.Toast;

**import** com.elinkcare.ubreath.desensitization.R;

**import** com.elinkcare.ubreath.desensitization.core.ClientManager;

**import** com.elinkcare.ubreath.desensitization.core.CommonCallback;

**import** com.elinkcare.ubreath.desensitization.core.data.UserInfo;

**import** com.elinkcare.ubreath.desensitization.utils.StateCodeUtils;

**import** com.elinkcare.ubreath.desensitization.widget.popwindow.BasePop;

**import** com.elinkcare.ubreath.desensitization.widget.popwindow.EditUserMailboxPop;

**import** com.elinkcare.ubreath.desensitization.widget.popwindow.EditUserPasswordPop;

*/***

** A simple {****@link*** *Fragment} subclass.*

**/*

**public class** AccountInfoFragment **extends** Fragment {

**private** View **mView**;

**private** View **backLayout**;

**private** TextView **userNameTextView**;

**private** TextView **accountNameTextView**;

**private** TextView **hospitalNameTextView**;

**private** TextView **hospitalDepartmentNameTextView**;

**private** View **childAccountManageLayout**;

**private** View **editPasswordLayout**;

**private** View **editMailboxLayout**;

**private** TextView **mailboxTextView**;

**private** Button **logoutButton**;

**private** ProgressBar **waitingProgressBar**;

**private** EditUserPasswordPop **mEditPasswordPop**;

**private** EditUserMailboxPop **mEditMailBoxPop**;

**private** View.OnClickListener **mChildAccountManageClickListener**;

**public** AccountInfoFragment() {

}

@Override

**public** View onCreateView(LayoutInflater inflater, ViewGroup container,

Bundle savedInstanceState) {

**mView** = inflater.inflate(R.layout.***fragment_account_info***, container, **false**);

initView();

initAction();

initPop();

initData();

**return mView**;

}

**private void** initView(){

**backLayout** = **mView**.findViewById(R.id.***ll_back***);

**userNameTextView** = (TextView) **mView**.findViewById(R.id.***tv_user_name***);

**accountNameTextView** = (TextView) **mView**.findViewById(R.id.***tv_account_name***);

**hospitalNameTextView** = (TextView) **mView**.findViewById(R.id.***tv_hospital***);

**hospitalDepartmentNameTextView** = (TextView) **mView**.findViewById(R.id.***tv_department***);

**childAccountManageLayout** = **mView**.findViewById(R.id.***fl_child_account***);

**editPasswordLayout** = **mView**.findViewById(R.id.***fl_password***);

**editMailboxLayout** = **mView**.findViewById(R.id.***ll_mailbox***);

**mailboxTextView** = (TextView) **mView**.findViewById(R.id.***tv_mailbox***);

**logoutButton** = (Button) **mView**.findViewById(R.id.***bt_logout***);

**waitingProgressBar** = (ProgressBar) **mView**.findViewById(R.id.***pb_waitting***);

}

**private void** initAction(){

**backLayout**.setOnClickListener(**new** View.OnClickListener() {

@Override

**public void** onClick(View view) {

getActivity().finish();

}

});

**logoutButton**.setOnClickListener(**new** View.OnClickListener() {

@Override

**public void** onClick(View view) {

logout();

}

});

**childAccountManageLayout**.setOnClickListener(**new** View.OnClickListener() {

@Override

**public void** onClick(View v) {

**if** (**mChildAccountManageClickListener** != **null**) {

**mChildAccountManageClickListener**.onClick(v);

}

}

});

**editPasswordLayout**.setOnClickListener(**new** View.OnClickListener() {

@Override

**public void** onClick(View view) {

**mEditPasswordPop**.show(**null**,**mView**);

}

});

**editMailboxLayout**.setOnClickListener(**new** View.OnClickListener() {

@Override

**public void** onClick(View view) {

**mEditMailBoxPop**.show(**null**,**mView**);

}

});

}

**private void** initData(){

setUpView();

ClientManager.*getInstance*().loadUserInfo(**new** CommonCallback() {

@Override

**public void** onSuccess() {

setUpView();

}

@Override

**public void** onError(String state) {

StateCodeUtils.*alert*(state,getContext());

}

});

}

**private void** setUpView(){

UserInfo userInfo = ClientManager.*getInstance*().getUserInfo();

**if** (userInfo != **null**){

**userNameTextView**.setText(userInfo.getName());

**accountNameTextView**.setText(userInfo.getAccountName());

**hospitalNameTextView**.setText(userInfo.getHospitalName());

**hospitalDepartmentNameTextView**.setText(userInfo.getHospitalDepartmentName());

**mailboxTextView**.setText(userInfo.getMailbox());

**switch** (userInfo.getUserPermission()){

**case** 0:

**childAccountManageLayout**.setVisibility(View.***VISIBLE***);

**break**;

**case** 1:

**childAccountManageLayout**.setVisibility(View.***GONE***);

**break**;

}

}

}

**private void** initPop(){

**mEditPasswordPop** = **new** EditUserPasswordPop(getContext());

**mEditMailBoxPop** = **new** EditUserMailboxPop(getContext());

**mEditMailBoxPop**.setOnPopSelectedListener(**new** BasePop.OnPopSelectedListener() {

@Override

**public void** onSelected(String selected) {

setUpView();

}

@Override

**public void** onCancel() {

}

});

}

**private void** logout(){

**if** (**waitingProgressBar**.getVisibility() == View.***VISIBLE***){

Toast.*makeText*(getContext(), **"系统繁忙"**, Toast.***LENGTH_SHORT***).show();

**return**;

}

**waitingProgressBar**.setVisibility(View.***VISIBLE***);

ClientManager.*getInstance*().logout(**new** CommonCallback() {

@Override

**public void** onSuccess() {

**waitingProgressBar**.setVisibility(View.***INVISIBLE***);

ClientManager.*getInstance*().clearAllCache();

Log.*e*(**"Test"**,**"clearChace"**);

}

@Override

**public void** onError(String state) {

StateCodeUtils.*alert*(state,getContext());

**waitingProgressBar**.setVisibility(View.***INVISIBLE***);

}

});

}

**public void** setChildAccountManageClickListener(View.OnClickListener listener){

**mChildAccountManageClickListener** = listener;

}

}**package** com.elinkcare.ubreath.desensitization.account.accountinfo;

**import** android.os.Bundle;

**import** android.support.annotation.Nullable;

**import** android.support.v4.app.Fragment;

**import** android.view.LayoutInflater;

**import** android.view.View;

**import** android.view.ViewGroup;

**import** android.widget.BaseAdapter;

**import** android.widget.ListView;

**import** android.widget.ProgressBar;

**import** android.widget.TextView;

**import** android.widget.Toast;

**import** com.elinkcare.ubreath.desensitization.R;

**import** com.elinkcare.ubreath.desensitization.core.ClientManager;

**import** com.elinkcare.ubreath.desensitization.core.CommonCallback;

**import** com.elinkcare.ubreath.desensitization.core.data.ChildAccountInfo;

**import** com.elinkcare.ubreath.desensitization.utils.StateCodeUtils;

**import** com.elinkcare.ubreath.desensitization.widget.popwindow.BasePop;

**import** com.elinkcare.ubreath.desensitization.widget.popwindow.EditChildAccountPop;

**import** com.elinkcare.ubreath.desensitization.widget.popwindow.MessageBoxPop;

**import** java.util.ArrayList;

**import** java.util.Collections;

**import** java.util.Comparator;

**import** java.util.List;

*/***

** Created by ${Ray} on 2017/3/27.*

**/*

**public class** ChildAccountFragment **extends** Fragment {

**private** View **mView**;

**private** View **backLayout**;

**private** TextView **addNewAccountTextView**;

**private** ProgressBar **waitingProgressBar**;

**private** ListView **accountListView**;

**private** AccountListViewAdapter **mAdapter**;

**private** List<ChildAccountInfo> **mAccountList** = **new** ArrayList<>();

**private** ChildAccountInfo **mSelectedChildAccountInfo** = **null**;

**private** View.OnClickListener **mBackClickListener**;

**private** MessageBoxPop **mConfirmDeletePop**;

**private** EditChildAccountPop **mEditAccountPop**;

@Nullable

@Override

**public** View onCreateView(LayoutInflater inflater, @Nullable ViewGroup container, @Nullable Bundle savedInstanceState) {

**mView** = inflater.inflate(R.layout.***fragment_account_child***, container, **false**);

initView();

initAction();

initData();

initPop();

**return mView**;

}

**private void** initView()

{

**backLayout** = **mView**.findViewById(R.id.***ll_back***);

**addNewAccountTextView** = (TextView) **mView**.findViewById(R.id.***tv_add_account***);

**waitingProgressBar** = (ProgressBar) **mView**.findViewById(R.id.***pb_waitting***);

**accountListView** = (ListView) **mView**.findViewById(R.id.***lv_account***);

**mAdapter** = **new** AccountListViewAdapter();

**accountListView**.setAdapter(**mAdapter**);

}

**private void** initAction()

{

**backLayout**.setOnClickListener(**new** View.OnClickListener() {

@Override

**public void** onClick(View v) {

**if** (**mBackClickListener** != **null**){

**mBackClickListener**.onClick(v);

}

}

});

**addNewAccountTextView**.setOnClickListener(**new** View.OnClickListener() {

@Override

**public void** onClick(View v) {

**mEditAccountPop**.show(**null**,**mView**);

}

});

}

**private void** initData()

{

setUpView();

loadChildAccountList();

}

**private synchronized void** setUpView()

{

List<ChildAccountInfo> accountInfos = ClientManager.*getInstance*().getChildAccountInfoList();

**if** (accountInfos != **null**){

**mAccountList**.clear();

**mAccountList**.addAll(accountInfos);

}

Collections.*sort*(**mAccountList**, **new** Comparator<ChildAccountInfo>() {

@Override

**public int** compare(ChildAccountInfo lhs, ChildAccountInfo rhs) {

**long** leftTime = lhs.getCreateTime();

**long** rightTime = rhs.getCreateTime();

**if** (leftTime < rightTime){

**return** 1;

}

**if** (leftTime > rightTime){

**return** -1;

}

**return** 0;

}

});

**mAdapter**.notifyDataSetChanged();

}

**private void** initPop()

{

**mConfirmDeletePop** = **new** MessageBoxPop(getContext());

**mConfirmDeletePop**.setTitle(**"账号删除"**);

**mConfirmDeletePop**.setOkButtonContent(**"确认"**);

**mConfirmDeletePop**.setOnCancelButtonContent(**"取消"**);

**mConfirmDeletePop**.setOnPopSelectedListener(**new** BasePop.OnPopSelectedListener() {

@Override

**public void** onSelected(String selected) {

deleteChildAccountInfo();

}

@Override

**public void** onCancel() {

}

});

**mEditAccountPop** = **new** EditChildAccountPop(getContext());

**mEditAccountPop**.setOnPopSelectedListener(**new** BasePop.OnPopSelectedListener() {

@Override

**public void** onSelected(String selected) {

loadChildAccountList();

}

@Override

**public void** onCancel() {

}

});

}

**private void** loadChildAccountList()

{

**if** (**waitingProgressBar**.getVisibility() == View.***VISIBLE***){

Toast.*makeText*(getContext(), **"系统繁忙"**, Toast.***LENGTH_SHORT***).show();

**return**;

}

**waitingProgressBar**.setVisibility(View.***VISIBLE***);

ClientManager.*getInstance*().loadChildAccountInfos(**new** CommonCallback() {

@Override

**public void** onSuccess() {

**waitingProgressBar**.setVisibility(View.***GONE***);

setUpView();

}

@Override

**public void** onError(String state) {

**waitingProgressBar**.setVisibility(View.***GONE***);

StateCodeUtils.*alert*(state,getContext());

}

});

}

**private void** deleteChildAccountInfo()

{

**if** (**mSelectedChildAccountInfo** == **null**) **return**;

**if** (**waitingProgressBar**.getVisibility() == View.***VISIBLE***){

Toast.*makeText*(getContext(), **"系统繁忙"**, Toast.***LENGTH_SHORT***).show();

**return**;

}

**waitingProgressBar**.setVisibility(View.***VISIBLE***);

ClientManager.*getInstance*().deleteChildAccountInfo(**mSelectedChildAccountInfo**.getId(),

**new** CommonCallback() {

@Override

**public void** onSuccess() {

**waitingProgressBar**.setVisibility(View.***GONE***);

loadChildAccountList();

}

@Override

**public void** onError(String state) {

**waitingProgressBar**.setVisibility(View.***GONE***);

StateCodeUtils.*alert*(state,getContext());

}

});

}

**private class** AccountListViewAdapter **extends** BaseAdapter{

@Override

**public int** getCount() {

**return mAccountList**.size();

}

@Override

**public** Object getItem(**int** position) {

**if** (**mAccountList**.size() != 0) **return mAccountList**.get(position);

**return "test"**;

}

@Override

**public long** getItemId(**int** position) {

**return** getItem(position).hashCode();

}

@Override

**public** View getView(**int** position, View convertView, ViewGroup parent) {

ViewHolder viewHolder;

**if** (convertView == **null**){

viewHolder = **new** ViewHolder();

convertView = LayoutInflater.*from*(getContext()).inflate(R.layout.***listitem_child_account*** , **null**);

viewHolder.**deleteTextView** = (TextView) convertView.findViewById(R.id.***tv_delete***);

viewHolder.**nameTextView** = (TextView) convertView.findViewById(R.id.***tv_name***);

viewHolder.**accountTextView** = (TextView) convertView.findViewById(R.id.***tv_account***);

viewHolder.**hospitalTextView** = (TextView) convertView.findViewById(R.id.***tv_hospital***);

viewHolder.**departmentTextView** = (TextView) convertView.findViewById(R.id.***tv_department***);

viewHolder.**splitView** = convertView.findViewById(R.id.***v_split***);

convertView.setTag(viewHolder);

}**else** {

viewHolder = (ViewHolder) convertView.getTag();

}

**final** ChildAccountInfo accountInfo = **mAccountList**.get(position);

viewHolder.**deleteTextView**.setOnClickListener(**new** View.OnClickListener() {

@Override

**public void** onClick(View v) {

**mSelectedChildAccountInfo** = accountInfo;

**mConfirmDeletePop**.setMessage(**"您确认删除"**+accountInfo.getPhone()+**"的账号吗?"**);

**mConfirmDeletePop**.show(**null**,**mView**);

}

});

viewHolder.**nameTextView**.setText(accountInfo.getRealName());

viewHolder.**accountTextView**.setText(accountInfo.getPhone());

viewHolder.**hospitalTextView**.setText(accountInfo.getHospitalName());

viewHolder.**departmentTextView**.setText(accountInfo.getDepartmentName());

**if** (position + 1 == getCount()){

viewHolder.**splitView**.setVisibility(View.***GONE***);

}**else** {

viewHolder.**splitView**.setVisibility(View.***VISIBLE***);

}

**return** convertView;

}

**private class** ViewHolder{

**private** TextView **deleteTextView**;

**private** TextView **nameTextView**;

**private** TextView **accountTextView**;

**private** TextView **hospitalTextView**;

**private** TextView **departmentTextView**;

**private** View **splitView**;

}

}

**public void** setOnBackClickListener(View.OnClickListener listener){

**mBackClickListener** = listener;

}

}wu**package** com.elinkcare.ubreath.desensitization.account;

**import** android.os.Bundle;

**import** android.support.v4.app.Fragment;

**import** android.view.LayoutInflater;

**import** android.view.View;

**import** android.view.ViewGroup;

**import** android.widget.AdapterView;

**import** android.widget.BaseAdapter;

**import** android.widget.ListView;

**import** android.widget.TextView;

**import** com.elinkcare.ubreath.desensitization.R;

**import** com.elinkcare.ubreath.desensitization.core.ClientManager;

**import** com.elinkcare.ubreath.desensitization.core.CommonCallback;

**import** com.elinkcare.ubreath.desensitization.core.data.OverviewPatient;

**import** com.elinkcare.ubreath.desensitization.core.data.PatientInfo;

**import** com.elinkcare.ubreath.desensitization.widget.popwindow.BasePop;

**import** com.elinkcare.ubreath.desensitization.widget.popwindow.PatientInfoEditPop;

**import** java.text.SimpleDateFormat;

**import** java.util.ArrayList;

**import** java.util.Collections;

**import** java.util.Comparator;

**import** java.util.List;

*/***

** A simple {****@link*** *Fragment} subclass.*

**/*

**public class** AllPatientsFragment **extends** Fragment {

**private** View **mView**;

**private** View **backLayout**;

**private** TextView **addNewPatientTextView**;

**private** TextView **treatmentPatientNumberTextView**;

**private** TextView **allPatientNumberTextView**;

**private** ListView **patientListView**;

**private** PatientListViewAdapter **mAdapter**;

**private** List<OverviewPatient> **mPatientList** = **new** ArrayList<>();

**private** PatientInfoEditPop **mAddPatientPop**;

**private int mNumber** = 10000;

**public** AllPatientsFragment() {

*// Required empty public constructor*

}

@Override

**public** View onCreateView(LayoutInflater inflater, ViewGroup container,

Bundle savedInstanceState) {

**mView** = inflater.inflate(R.layout.***fragment_all_patients***, container, **false**);

initView();

initAction();

initPop();

initData();

**return mView**;

}

@Override

**public void** onResume() {

**super**.onResume();

**mAddPatientPop**.reloadChildPops();

}

**private void** initView(){

**backLayout** = **mView**.findViewById(R.id.***ll_back***);

**addNewPatientTextView** = (TextView) **mView**.findViewById(R.id.***tv_add_patient***);

**treatmentPatientNumberTextView** = (TextView) **mView**.findViewById(R.id.***tv_patient_treatment***);

**allPatientNumberTextView** = (TextView) **mView**.findViewById(R.id.***tv_patient_all***);

**patientListView** = (ListView) **mView**.findViewById(R.id.***lv_all_patients***);

**mAdapter** = **new** PatientListViewAdapter();

**patientListView**.setAdapter(**mAdapter**);

}

**private void** initAction(){

**backLayout**.setOnClickListener(**new** View.OnClickListener() {

@Override

**public void** onClick(View view) {

getActivity().finish();

}

});

**addNewPatientTextView**.setOnClickListener(**new** View.OnClickListener() {

@Override

**public void** onClick(View view) {

**mAddPatientPop**.show(**null**,**mView**);

}

});

**patientListView**.setOnItemClickListener(**new** AdapterView.OnItemClickListener() {

@Override

**public void** onItemClick(AdapterView<?> adapterView, View view, **int** i, **long** l) {

**if** (i == **mPatientList**.size()) **return**;

OverviewPatient patient = **mPatientList**.get(i);

PatientInfo patientInfo = ClientManager.*getInstance*().getPatient(patient.getId());

**if** (patientInfo != **null**){

ClientManager.*getInstance*().setCurrentPatient(patientInfo);

getActivity().finish();

}

}

});

}

**private void** initData(){

setUpView();

loadPatient(1);

}

**public synchronized void** loadPatient(**int** page){

ClientManager.*getInstance*().loadAllOverviewPatients(page, **mNumber**, **new** CommonCallback() {

@Override

**public void** onSuccess() {

setUpView();

}

@Override

**public void** onError(String state) {

}

});

}

**private synchronized int** getCurrentInTherapyPatientNumber(){

**int** patientInTherapyNumber = 0;

OverviewPatient patient;

**for** (**int** i = 0; i < **mPatientList**.size(); i++) {

patient = **mPatientList**.get(i);

**if** (patient.getTherapyStatus() == 0){

patientInTherapyNumber ++;

}

}

**return** patientInTherapyNumber;

}

**private synchronized void** setUpView(){

List<OverviewPatient> patients = ClientManager.*getInstance*().getAllOverviewPatients();

**if** (patients != **null**){

**mPatientList**.clear();

**mPatientList**.addAll(patients);

sortPatientListByTime(**mPatientList**);

}

**treatmentPatientNumberTextView**.setText(String.*valueOf*(getCurrentInTherapyPatientNumber()));

**allPatientNumberTextView**.setText(String.*valueOf*(**mPatientList**.size()));

**mAdapter**.notifyDataSetChanged();

}

**private void** initPop(){

**mAddPatientPop** = **new** PatientInfoEditPop(getContext());

**mAddPatientPop**.setPatient(**null**);

**mAddPatientPop**.setOnPopSelectedListener(**new** BasePop.OnPopSelectedListener() {

@Override

**public void** onSelected(String selected) {

**mAddPatientPop**.setPatient(**null**);

loadPatient(1);

}

@Override

**public void** onCancel() {

}

});

}

**private void** sortPatientListByTime(List<OverviewPatient> patients){

**if** (patients == **null**) **return**;

Collections.*sort*(patients, **new** Comparator<OverviewPatient>() {

@Override

**public int** compare(OverviewPatient lhs, OverviewPatient rhs) {

**int** statusReslut = lhs.getTherapyStatus() - rhs.getTherapyStatus();

**if** (statusReslut == 0){

**long** result = lhs.getInjectionDate() - rhs.getInjectionDate();

**if** (result < 0) **return** 1;

**if** (result > 0) **return** -1;

}

**return** statusReslut;

}

});

}

**private class** PatientListViewAdapter **extends** BaseAdapter{

**private** SimpleDateFormat **mFormat** = **new** SimpleDateFormat(**"yyyy-MM-dd"**);

@Override

**public int** getCount() {

**return mPatientList**.size();

}

@Override

**public** Object getItem(**int** i) {

**if** (**mPatientList**.size() == 0)

**return "test"**;

**return mPatientList**.get(i);

}

@Override

**public long** getItemId(**int** i) {

**return** getItem(i).hashCode();

}

@Override

**public** View getView(**int** i, View view, ViewGroup viewGroup) {

ViewHolder viewHolder;

**if** (view == **null**){

view = LayoutInflater.*from*(getContext()).inflate(R.layout.***listitem_all_patients*** ,**null**);

viewHolder = **new** ViewHolder();

viewHolder.**orderTextView** = (TextView) view.findViewById(R.id.***tv_order***);

viewHolder.**nameTextView** = (TextView) view.findViewById(R.id.***tv_name***);

viewHolder.**numberTextView** = (TextView) view.findViewById(R.id.***tv_number***);

viewHolder.**treatmentStateTextView** = (TextView) view.findViewById(R.id.***tv_treat_state***); viewHolder.**currentInjectionNumberTextView**=(TextView)view.findViewById(R.id.***tv_current_injection_number***);

viewHolder.**injectionTimeTextView** = (TextView) view.findViewById(R.id.***tv_injection_time***);

viewHolder.**spliteLine** = view.findViewById(R.id.***v_splide_line***);

view.setTag(viewHolder);

}**else** {

viewHolder = (ViewHolder) view.getTag();

}

viewHolder.**orderTextView**.setText(i+1+**""**);

OverviewPatient patient = **mPatientList**.get(i);

viewHolder.**nameTextView**.setText(patient.getName());

viewHolder.**numberTextView**.setText(patient.getNumber());

**switch** (patient.getTherapyStatus()){

**case** 0:

viewHolder.**treatmentStateTextView**.setText(**"治疗中"**);

**break**;

**case** 1:

viewHolder.**treatmentStateTextView**.setText(**"结束治疗"**);

**break**;

}

String injectionNumber = patient.getInjectionNumber();

**if** (**"0"**.equals(injectionNumber)){

viewHolder.**currentInjectionNumberTextView**.setText(**"--"**);

}**else** {

viewHolder.**currentInjectionNumberTextView**.setText(patient.getInjectionNumber());

}

**long** injectionTime = patient.getInjectionDate();

**if** (injectionTime == 0){

viewHolder.**injectionTimeTextView**.setText(**"--"**);

}**else** {

viewHolder.**injectionTimeTextView**.setText(**mFormat**.format(injectionTime*1000));

}

**if** (i+1 == getCount()){

viewHolder.**spliteLine**.setVisibility(View.***INVISIBLE***);

}**else** {

viewHolder.**spliteLine**.setVisibility(View.***VISIBLE***);

}

**return** view;

}

**private class** ViewHolder{

**private** TextView **orderTextView**;

**private** TextView **nameTextView**;

**private** TextView **numberTextView**;

**private** TextView **treatmentStateTextView**;

**private** TextView **currentInjectionNumberTextView**;

**private** TextView **injectionTimeTextView**;

**private** View **spliteLine**;

}

}

}

**package** com.elinkcare.ubreath.desensitization.account.exportfile;

**import** android.os.Bundle;

**import** android.support.annotation.Nullable;

**import** android.support.v4.app.Fragment;

**import** android.support.v4.app.FragmentManager;

**import** android.support.v4.app.FragmentStatePagerAdapter;

**import** android.view.LayoutInflater;

**import** android.view.View;

**import** android.view.ViewGroup;

**import** com.elinkcare.ubreath.desensitization.R;

**import** com.elinkcare.ubreath.desensitization.widget.FragmentNavigationViewPager;

**import** java.io.File;

*/***

** Created by ${Ray} on 2017/4/5.*

**/*

**public class** ExportFileFragmentManager **extends** Fragment {

**private** View **mView**;

**private** FragmentNavigationViewPager **mViewPager**;

**private** ExportFileManageViewPagerAdapter **mAdapter**;

**private** ExportFilesFragment **mExportFileFragment**;

**private** DownLoadFileFragment **mDownLoadFileFragment**;

**private** CreatePDFFileListener **mListener**;

@Nullable

@Override

**public** View onCreateView(LayoutInflater inflater, @Nullable ViewGroup container, @Nullable Bundle savedInstanceState)

{

**mView** = inflater.inflate(R.layout.***fragment_export_file_manager***, container, **false**);

initFragment();

initView();

initListener();

**return mView**;

}

**private void** initView()

{

**mViewPager** = (FragmentNavigationViewPager) **mView**.findViewById(R.id.***vp_file***);

**mViewPager**.setScrollble(**false**);

**mAdapter** = **new** ExportFileManageViewPagerAdapter(getChildFragmentManager());

**mViewPager**.setAdapter(**mAdapter**);

}

**private void** initListener()

{

**mExportFileFragment**.setOnExportClickListener(**new** View.OnClickListener() {

@Override

**public void** onClick(View v) {

**mViewPager**.setCurrentItem(1);

}

});

**mExportFileFragment**.setCreatePDFFileListener(**mDownLoadFileFragment**.getCreateFileListener());

}

**private void** initFragment()

{

**mExportFileFragment** = **new** ExportFilesFragment();

**mDownLoadFileFragment** = **new** DownLoadFileFragment();

}

**private class** ExportFileManageViewPagerAdapter **extends** FragmentStatePagerAdapter{

**public** ExportFileManageViewPagerAdapter(FragmentManager fm) {

**super**(fm);

}

@Override

**public** Fragment getItem(**int** position) {

**switch** (position)

{

**case** 0:

**return mExportFileFragment**;

**case** 1:

**return mDownLoadFileFragment**;

**default**:

**return null**;

}

}

@Override

**public int** getCount() {

**return** 2;

}

}

**public interface** CreatePDFFileListener

{

**public void** onStartCreate(File file);

**public void** onCreateFinished(File file);

}

}

**package** com.elinkcare.ubreath.desensitization.account.exportfile;

**import** android.graphics.Color;

**import** android.os.Bundle;

**import** android.os.Environment;

**import** android.support.v4.app.Fragment;

**import** android.view.LayoutInflater;

**import** android.view.View;

**import** android.view.ViewGroup;

**import** android.widget.AbsListView;

**import** android.widget.BaseAdapter;

**import** android.widget.ListView;

**import** android.widget.ProgressBar;

**import** android.widget.TextView;

**import** android.widget.Toast;

**import** com.elinkcare.ubreath.desensitization.R;

**import** com.elinkcare.ubreath.desensitization.core.ClientManager;

**import** com.elinkcare.ubreath.desensitization.core.CommonCallback;

**import** com.elinkcare.ubreath.desensitization.core.data.DesensitizationRecordInfo;

**import** com.elinkcare.ubreath.desensitization.core.data.PatientDesensitizationRecordInfo;

**import** com.elinkcare.ubreath.desensitization.utils.StateCodeUtils;

**import** com.elinkcare.ubreath.desensitization.widget.DesensitizationHorizontalScrollView;

**import** com.elinkcare.ubreath.desensitization.widget.popwindow.BasePop;

**import** com.elinkcare.ubreath.desensitization.widget.popwindow.FloatDatePickerPop;

**import** com.itextpdf.text.Document;

**import** com.itextpdf.text.DocumentException;

**import** com.itextpdf.text.Element;

**import** com.itextpdf.text.Font;

**import** com.itextpdf.text.PageSize;

**import** com.itextpdf.text.Paragraph;

**import** com.itextpdf.text.Rectangle;

**import** com.itextpdf.text.pdf.BaseFont;

**import** com.itextpdf.text.pdf.PdfPCell;

**import** com.itextpdf.text.pdf.PdfPTable;

**import** com.itextpdf.text.pdf.PdfWriter;

**import** java.io.File;

**import** java.io.FileNotFoundException;

**import** java.io.FileOutputStream;

**import** java.io.IOException;

**import** java.text.SimpleDateFormat;

**import** java.util.ArrayList;

**import** java.util.Calendar;

**import** java.util.Collections;

**import** java.util.Comparator;

**import** java.util.List;

*/***

** A simple {****@link*** *Fragment} subclass.*

**/*

**public class** ExportFilesFragment **extends** Fragment {

**private** View **mView**;

**private** View **backLayout**;

**private** View **startTimeLayout**;

**private** View **endTimeLayout**;

**private** TextView **startTimeTextView**;

**private** TextView **endTimeTextView**;

**private** TextView **weekRecordTextView**;

**private** TextView **monthRecordTextView**;

**private** DesensitizationHorizontalScrollView **desensitionHorizontalScrollView**;

**private** View **desentitizationRecordDevider**;

**private** TextView **exportFileTextView**;

**private** ProgressBar **waitProgressBar**;

**private** ListView **desensitizationRecordHeaderListView**;

**private** HeaderListViewAdapter **mHeaderListViewAdapter**;

**private** ListView **desensitizationRecordListView**;

**private** DesensitizationRecordListViewAdapter **mDesensitizationRecordListViewAdapter**;

**private long mStartTime**;

**private long mEndTime**;

**private** SimpleDateFormat **mFormat** = **new** SimpleDateFormat(**"yyyy年MM月dd日"**);

**private** SimpleDateFormat **dateFormat** = **new** SimpleDateFormat(**"MM-dd"**);

**private** SimpleDateFormat **timeFormat** = **new** SimpleDateFormat(**"HH:mm"**);

**private** List<PatientDesensitizationRecordInfo> **mRecords** = **new** ArrayList<>();

**private** FloatDatePickerPop **mStartTimeDatePickerPop**;

**private** FloatDatePickerPop **mEndTimeDataPickerPop**;

**private** View.OnClickListener **mExportClickListener**;

**private** ExportFileFragmentManager.CreatePDFFileListener **mListener**;

**public** ExportFilesFragment() {

*// Required empty public constructor*

}

@Override

**public** View onCreateView(LayoutInflater inflater, ViewGroup container,

Bundle savedInstanceState) {

**mView** = inflater.inflate(R.layout.***fragment_export_files***, container, **false**);

initView();

initAction();

initPop();

initTime();

initData();

**return mView**;

}

**private void** initView(){

**backLayout** = **mView**.findViewById(R.id.***ll_back***);

**startTimeLayout** = **mView**.findViewById(R.id.***ll_start_time***);

**startTimeTextView** = (TextView) **mView**.findViewById(R.id.***tv_start_time***);

**endTimeLayout** = **mView**.findViewById(R.id.***ll_end_time***);

**endTimeTextView** = (TextView) **mView**.findViewById(R.id.***tv_end_time***);

**weekRecordTextView** = (TextView) **mView**.findViewById(R.id.***tv_week_record***);

**monthRecordTextView** = (TextView) **mView**.findViewById(R.id.***tv_month_recod***);

**desentitizationRecordDevider** = **mView**.findViewById(R.id.***v_desensitization_divider***);

**exportFileTextView** = (TextView) **mView**.findViewById(R.id.***tv_export_file***);

**waitProgressBar** = (ProgressBar) **mView**.findViewById(R.id.***pb_waitting***);

**desensitizationRecordHeaderListView** = (ListView)

**mView**.findViewById(R.id.***lv_desensitization_header***);

**mHeaderListViewAdapter** = **new** HeaderListViewAdapter();

**desensitizationRecordHeaderListView**.setAdapter(**mHeaderListViewAdapter**);

**desensitizationRecordListView** = (ListView) **mView**.findViewById(R.id.***lv_desensitization_records***);

**mDesensitizationRecordListViewAdapter** = **new** DesensitizationRecordListViewAdapter();

**desensitizationRecordListView**.setAdapter(**mDesensitizationRecordListViewAdapter**);

**desensitionHorizontalScrollView** = (DesensitizationHorizontalScrollView) **mView**.findViewById(R.id.***hs_desensitization***);

}

**private void** initAction(){

**backLayout**.setOnClickListener(**new** View.OnClickListener() {

@Override

**public void** onClick(View view) {

getActivity().finish();

}

});

**startTimeLayout**.setOnClickListener(**new** View.OnClickListener() {

@Override

**public void** onClick(View view) {

**mStartTimeDatePickerPop**.setSelect(**mStartTime***1000);

**mStartTimeDatePickerPop**.show(**null**,**startTimeLayout**);

}

});

**endTimeLayout**.setOnClickListener(**new** View.OnClickListener() {

@Override

**public void** onClick(View view) {

**mEndTimeDataPickerPop**.setSelect(**mEndTime***1000);

**mEndTimeDataPickerPop**.show(**null**,**endTimeLayout**);

}

});

**weekRecordTextView**.setOnClickListener(**new** View.OnClickListener() {

@Override

**public void** onClick(View view) {

**mStartTime** = getTimeOfFirstDayOfWeek()/1000;

**mEndTime** = Calendar.*getInstance*().getTimeInMillis()/1000;

setUpView();

}

});

**monthRecordTextView**.setOnClickListener(**new** View.OnClickListener() {

@Override

**public void** onClick(View view) {

**mStartTime** = getTimeOfFirstDayOfMonth()/1000;

**mEndTime** = Calendar.*getInstance*().getTimeInMillis()/1000;

setUpView();

}

});

AbsListView.OnScrollListener scrollListener = **new** AbsListView.OnScrollListener() {

**private int mFirstVisibleItem**;

**private int mFirstVisibleTop**;

@Override

**public void** onScrollStateChanged(AbsListView absListView, **int** i) {

}

@Override

**public void** onScroll(AbsListView absListView, **int** firstVisableItem, **int** visableItemCount, **int** totalItemCount) {

**if** (absListView.getChildCount() == 0) **return**;

View topView = absListView.getChildAt(0);

**if** (topView == **null**) **return**;

**int** top = topView.getTop();

**if** ( **mFirstVisibleItem** == firstVisableItem && **mFirstVisibleTop** == top) **return**;

**if**(**desensitizationRecordHeaderListView** == absListView)

{

**desensitizationRecordListView**.setSelectionFromTop(firstVisableItem, top);

}**else** {

**desensitizationRecordHeaderListView**.setSelectionFromTop(firstVisableItem, top);

}

**mFirstVisibleItem** = firstVisableItem;

**mFirstVisibleTop** = top;

}

};

**desensitizationRecordHeaderListView**.setOnScrollListener(scrollListener);

**desensitizationRecordListView**.setOnScrollListener(scrollListener);

**desensitizationRecordHeaderListView**.setOverScrollMode(ListView.***OVER_SCROLL_NEVER***);

**desensitizationRecordListView**.setOverScrollMode(ListView.***OVER_SCROLL_NEVER***);

**desensitionHorizontalScrollView**.setOnScrollchangedListener(**new** DesensitizationHorizontalScrollView.OnScrollChangedListener() {

@Override

**public void** onScrollChanged(**int** scrollX, **int** scrollY) {

**if** (scrollX == 0){

**desentitizationRecordDevider**.setVisibility(View.***GONE***);

}**else** {

**desentitizationRecordDevider**.setVisibility(View.***VISIBLE***);

}

}

});

**exportFileTextView**.setOnClickListener(**new** View.OnClickListener() {

@Override

**public void** onClick(View v) {

*// if (mExportClickListener != null)*

*// {*

*// mExportClickListener.onClick(v);*

*// }*

exportFile();

}

});

}

**private void** initData(){

setUpView();

refreshRecordsListView();

}

**private void** initPop(){

**mStartTimeDatePickerPop** = **new** FloatDatePickerPop(getContext());

**mStartTimeDatePickerPop**.setOnPopSelectedListener(**new** BasePop.OnPopSelectedListener() {

@Override

**public void** onSelected(String selected) {

**try** {

**long** dateTime = Long.*valueOf*(selected);

Calendar calendar = Calendar.*getInstance*();

**if** (dateTime > getTimeOfBeginOfToday()) {

**mStartTime** = getTimeOfBeginOfToday() / 1000;

} **else** {

calendar.setTimeInMillis(dateTime);

}

calendar.set(Calendar.***HOUR_OF_DAY***, 0);

calendar.set(Calendar.***MINUTE***, 0);

calendar.set(Calendar.***SECOND***, 0);

**mStartTime** = calendar.getTimeInMillis() / 1000;

} **catch** (NumberFormatException e) {

e.printStackTrace();

}

setUpView();

}

@Override

**public void** onCancel() {

}

});

**mEndTimeDataPickerPop** = **new** FloatDatePickerPop(getContext());

**mEndTimeDataPickerPop**.setOnPopSelectedListener(**new** BasePop.OnPopSelectedListener() {

@Override

**public void** onSelected(String selected) {

**try** {

**long** dateTime = Long.*valueOf*(selected);

Calendar calendar = Calendar.*getInstance*();

**if** (dateTime > getTimeOfEndOfToday()) {

**mEndTime** = getTimeOfEndOfToday()/1000;

} **else** {

calendar.setTimeInMillis(dateTime);

}

calendar.set(Calendar.***HOUR_OF_DAY***, 23);

calendar.set(Calendar.***MINUTE***, 59);

calendar.set(Calendar.***SECOND***, 59);

**mEndTime** = calendar.getTimeInMillis() / 1000;

} **catch** (NumberFormatException e) {

e.printStackTrace();

}

setUpView();

}

@Override

**public void** onCancel() {

}

});

}

**private void** initTime(){

**mStartTime** = getTimeOfFirstDayOfWeek()/1000;

**mEndTime** = getTimeOfEndOfToday()/1000;

}

**private void** setUpView() {

**if** (**mStartTime** > **mEndTime**) {

**long** tempTime = **mEndTime**;

**mEndTime** = **mStartTime** + 24 * 3600 - 1;

**mStartTime** = tempTime - 24 * 3600 + 1;

}

**startTimeTextView**.setText(**mFormat**.format(**mStartTime** * 1000) + **"AM"**);

**endTimeTextView**.setText(**mFormat**.format(**mEndTime** * 1000) + **"PM"**);

loadPatientDesensitizationRecordInfo();

}

**private synchronized void** refreshRecordsListView(){

List<PatientDesensitizationRecordInfo> recordInfos = ClientManager.*getInstance*().getPatientDesensitizationRecords();

**if** (recordInfos != **null**){

**mRecords**.clear();

**mRecords**.addAll(recordInfos);

}

Collections.*sort*(**mRecords**, **new** Comparator<PatientDesensitizationRecordInfo>() {

@Override

**public int** compare(PatientDesensitizationRecordInfo lhs, PatientDesensitizationRecordInfo rhs) {

DesensitizationRecordInfo lhsRecordInfo = lhs.getDesensitizationRecordInfo();

DesensitizationRecordInfo rhsRecordInfo = rhs.getDesensitizationRecordInfo();

Calendar calDate = Calendar.*getInstance*();

Calendar calTime = Calendar.*getInstance*();

calDate.setTimeInMillis(lhsRecordInfo.getInjectDate()*1000);

calTime.setTimeInMillis(lhsRecordInfo.isInjectSeparate()? lhsRecordInfo.getInjectTime2()*1000:lhsRecordInfo.getInjectTime1()*1000);

calTime.set(calDate.get(Calendar.***YEAR***),calDate.get(Calendar.***MONTH***),calDate.get(Calendar.***HOUR_OF_DAY***));

**long** leftInjectionTime = calTime.getTimeInMillis();

calDate.setTimeInMillis(rhsRecordInfo.getInjectDate()*1000);

calTime.setTimeInMillis(rhsRecordInfo.isInjectSeparate()?rhsRecordInfo.getInjectTime2()*1000:rhsRecordInfo.getInjectTime1()*1000); calTime.set(calDate.get(Calendar.***YEAR***),calDate.get(Calendar.***MONTH***),calDate.get(Calendar.***HOUR_OF_DAY***)); **long** rightInjectionTime = calTime.getTimeInMillis();

**if** (leftInjectionTime > rightInjectionTime) **return** -1;

**if** (leftInjectionTime < rightInjectionTime) **return** 1;

**return** 0;

}

});

**mDesensitizationRecordListViewAdapter**.notifyDataSetChanged();

**mHeaderListViewAdapter**.notifyDataSetChanged();

}

**private void** loadPatientDesensitizationRecordInfo(){

**if** (**waitProgressBar**.getVisibility() == View.***VISIBLE***){

Toast.*makeText*(getContext(), **"系统繁忙"**, Toast.***LENGTH_SHORT***).show();

**return**;

}

**waitProgressBar**.setVisibility(View.***VISIBLE***);

ClientManager.*getInstance*().loadPatientDesensitizationRecordInfoByTime(**mStartTime**, **mEndTime**, **new** CommonCallback() {

@Override

**public void** onSuccess() {

**waitProgressBar**.setVisibility(View.***GONE***);

refreshRecordsListView();

}

@Override

**public void** onError(String state) {

**waitProgressBar**.setVisibility(View.***GONE***);

StateCodeUtils.*alert*(state,getContext());

}

});

}

**private long** getTimeOfFirstDayOfWeek(){

Calendar calendar = Calendar.*getInstance*();

calendar.set(Calendar.***DAY_OF_WEEK***,Calendar.***MONDAY***);

calendar.set(Calendar.***HOUR_OF_DAY***,0);

calendar.set(Calendar.***MINUTE***,0);

calendar.set(Calendar.***SECOND***,0);

**return** calendar.getTimeInMillis();

}

**private long** getTimeOfFirstDayOfMonth(){

Calendar calendar = Calendar.*getInstance*();

calendar.set(Calendar.***DAY_OF_MONTH***,1);

calendar.set(Calendar.***HOUR_OF_DAY***,0);

calendar.set(Calendar.***MINUTE***,0);

calendar.set(Calendar.***SECOND***,0);

**return** calendar.getTimeInMillis();

}

**private long** getTimeOfEndOfToday(){

Calendar calendar = Calendar.*getInstance*();

calendar.set(Calendar.***HOUR_OF_DAY***,23);

calendar.set(Calendar.***MINUTE***,59);

calendar.set(Calendar.***SECOND***,59);

**return** calendar.getTimeInMillis();

}

**private long** getTimeOfBeginOfToday(){

Calendar calendar = Calendar.*getInstance*();

calendar.set(Calendar.***HOUR_OF_DAY***,0);

calendar.set(Calendar.***MINUTE***,0);

calendar.set(Calendar.***SECOND***,0);

**return** calendar.getTimeInMillis();

}

**private void** exportFile()

{

**new** Thread(){

@Override

**public void** run() {

createPEFFile();

}

}.start();

}

**private synchronized void** createPEFFile()

{

**if** (!Environment.*getExternalStorageState*().equals(Environment.***MEDIA_MOUNTED***))

{

Toast.*makeText*(getContext(), **"没有sd卡，创建失败"**, Toast.***LENGTH_SHORT***).show();

**return**;

}

String path = Environment.*getExternalStorageDirectory*() + **"/Ubreath_DesensitizationRecorder"**;

File dir = **new** File(path);

**if** (!dir.exists()) {

dir.mkdirs();

}

File file = **new** File(dir,**mFormat**.format(**mStartTime***1000)+**"--"**+**mFormat**.format(**mEndTime***1000)+**".pdf"**);

**if** (**mListener** != **null**)

{

**mListener**.onStartCreate(file);

}

Rectangle rect = **new** Rectangle(PageSize.***A4***.rotate());

rect.setBorderWidth(5);

Document document = **new** Document(rect,5,5,15,5);

**try** {

FileOutputStream outputStream = **new** FileOutputStream(file);

**try** {

PdfWriter.*getInstance*(document,outputStream);

document.open();

BaseFont baseFont = **null**;

Font chineseFont = **null**;

**try** {

baseFont = BaseFont.*createFont*(**"STSong-Light"**,**"UniGB-UCS2-H"**,BaseFont.***NOT_EMBEDDED***);

chineseFont = **new** Font(baseFont,10.5f);

} **catch** (IOException e) {

e.printStackTrace();

}

*// 标题一;*

Font paraFont = **new** Font(baseFont ,20f);

Paragraph titleParagraph = **new** Paragraph(**"患者治疗记录"**,paraFont);

titleParagraph.setAlignment(Element.***ALIGN_CENTER***);

document.add(titleParagraph);

PdfPTable table = **new** PdfPTable(**new float**[]{14,15,12,15,12,12,15,10,12,14,15,22,15,24,20,20,15});

table.setWidthPercentage(100);

Paragraph titleNumber = **new** Paragraph(**"编号"**,chineseFont);

table.addCell(getContentCenterPdfCell(titleNumber));

Paragraph titleName = **new** Paragraph(**"姓名"**,chineseFont);

table.addCell(getContentCenterPdfCell(titleName));

Paragraph titleWeekNum = **new** Paragraph(**"周次"**,chineseFont);

table.addCell(getContentCenterPdfCell(titleWeekNum));

Paragraph titleInjectionDate = **new** Paragraph(**"注射\n日期"**,chineseFont);

table.addCell(getContentCenterPdfCell(titleInjectionDate));

Paragraph titleInjectionNumber = **new** Paragraph(**"注射\n序号"**,chineseFont);

table.addCell(getContentCenterPdfCell(titleInjectionNumber));

Paragraph titleHealthStatus = **new** Paragraph(**"状态"**,chineseFont);

table.addCell(getContentCenterPdfCell(titleHealthStatus));

Paragraph titleBeforePEF = **new** Paragraph(**"PEF\n(前)"**,chineseFont);

table.addCell(getContentCenterPdfCell(titleBeforePEF));

Paragraph titelInjectionSeparate = **new** Paragraph(**"分针"**,chineseFont);

table.addCell(getContentCenterPdfCell(titelInjectionSeparate));

Paragraph titleBottleNum = **new** Paragraph(**"瓶号"**,chineseFont);

table.addCell(getContentCenterPdfCell(titleBottleNum));

Paragraph titelDose = **new** Paragraph(**"容量\n(ml)"**,chineseFont);

table.addCell(getContentCenterPdfCell(titelDose));

Paragraph titleInjectionPart = **new** Paragraph(**"注射\n部位"**,chineseFont);

table.addCell(getContentCenterPdfCell(titleInjectionPart));

Paragraph titleInjectionTime = **new** Paragraph(**"注射\n时间"**,chineseFont);

table.addCell(getContentCenterPdfCell(titleInjectionTime));

Paragraph titleAfterPEF = **new** Paragraph(**"PEF\n(后)"**,chineseFont);

table.addCell(getContentCenterPdfCell(titleAfterPEF));

Paragraph titleimmWhealSize = **new** Paragraph(**"即刻局部\n风团大小"**,chineseFont);

table.addCell(getContentCenterPdfCell(titleimmWhealSize));

Paragraph titleimmReaction = **new** Paragraph(**"即刻反\n应记录"**,chineseFont);

table.addCell(getContentCenterPdfCell(titleimmReaction));

Paragraph titleDelayReaction = **new** Paragraph(**"迟发反\n应记录"**,chineseFont);

table.addCell(getContentCenterPdfCell(titleDelayReaction));

Paragraph titleResponsiblePerson = **new** Paragraph(**"责任人"**,chineseFont);

table.addCell(getContentCenterPdfCell(titleResponsiblePerson));

table.setHeaderRows(1);

**for** (**int** i = 0; i < **mRecords**.size(); i++) {

PatientDesensitizationRecordInfo patientDesensitizationRecordInfo = **mRecords**.get(i);

table.addCell(getContentCenterPdfCell(**new** Paragraph(patientDesensitizationRecordInfo.getPatientNumber())));

table.addCell(getContentCenterPdfCell(**new** Paragraph(patientDesensitizationRecordInfo.getPatientName(),chineseFont)));

DesensitizationRecordInfo recordInfo = patientDesensitizationRecordInfo.getDesensitizationRecordInfo();

table.addCell(getContentCenterPdfCell(**new** Paragraph(String.*valueOf*(recordInfo.getWeekNumber()))));

table.addCell(getContentCenterPdfCell(**new** Paragraph(**dateFormat**.format(recordInfo.getInjectDate()*1000))));

table.addCell(getContentCenterPdfCell(**new** Paragraph(String.*valueOf*(recordInfo.getInjectNumber()))));

table.addCell(getContentCenterPdfCell(**new** Paragraph(recordInfo.getHealthStatus(),chineseFont)));

**if** (recordInfo.getBeforePEF() == 0)

{

table.addCell(getContentCenterPdfCell(**new** Paragraph(**""**)));

}**else** {

table.addCell(getContentCenterPdfCell(**new** Paragraph(String.*valueOf*(String.*valueOf*(recordInfo.getBeforePEF())))));

}

table.addCell(getContentCenterPdfCell(**new** Paragraph(recordInfo.isInjectSeparate()? **"是"**:**"否"**,chineseFont)));

table.addCell(getContentCenterPdfCell(**new** Paragraph(String.*valueOf*(recordInfo.getBottleNumber()))));

table.addCell(getContentCenterPdfCell(**new** Paragraph(recordInfo.getInjectDose())));

**if** (recordInfo.isInjectSeparate()){

table.addCell(getContentCenterPdfCell(

**new** Paragraph(recordInfo.getInjectPart1()+**"/"**+recordInfo.getInjectPart2(),chineseFont)));

table.addCell(getContentCenterPdfCell(

**new** Paragraph(**timeFormat**.format(recordInfo.getInjectTime1()*1000)+**"/"**+**timeFormat**.format(recordInfo.getInjectTime2()*1000))));

}**else** {

table.addCell(getContentCenterPdfCell(**new** Paragraph(recordInfo.getInjectPart1(),chineseFont)));

table.addCell(getContentCenterPdfCell(**new** Paragraph(**timeFormat**.format(recordInfo.getInjectTime1()*1000))));

}

**if** (recordInfo.getAfterPEF() == 0)

{

table.addCell(getContentCenterPdfCell(**new** Paragraph(**""**)));

}**else** {

table.addCell(getContentCenterPdfCell(**new** Paragraph(String.*valueOf*(recordInfo.getAfterPEF()))));

}

table.addCell(getContentCenterPdfCell(**new** Paragraph(fixMultiPicksOrder(recordInfo.getImmedWhealSize()),chineseFont)));

table.addCell(getContentCenterPdfCell(**new** Paragraph(fixMultiPicksOrder(recordInfo.getImmedReaction()),chineseFont)));

table.addCell(getContentCenterPdfCell(**new** Paragraph(fixMultiPicksOrder(recordInfo.getDelayReaction()),chineseFont)));

table.addCell(getContentCenterPdfCell(**new** Paragraph(patientDesensitizationRecordInfo.getResponsiblePerson(),chineseFont)));

}

table.setSpacingBefore(20f);

document.add(table);

document.close();

**if** (**mListener** != **null**)

{

**mListener**.onCreateFinished(file);

}

} **catch** (DocumentException e) {

e.printStackTrace();

}

} **catch** (FileNotFoundException e) {

e.printStackTrace();

}**finally** {

}

}

**private** PdfPCell getContentCenterPdfCell(Paragraph paragraph)

{

PdfPCell cell = **new** PdfPCell(paragraph);

cell.setHorizontalAlignment(Element.***ALIGN_CENTER***);

cell.setVerticalAlignment(Element.***ALIGN_MIDDLE***);

cell.setPadding(3.0f);

**return** cell;

}

**private** String fixMultiPicksOrder(String content)

{

**if** (content == **null**) **return null**;

StringBuilder builder = **new** StringBuilder();

String[] itmes = content.split(**"\\s+"**);

**if** (itmes != **null**)

{

**for** (**int** i = 0; i < itmes.**length**; i++)

{

builder.append(itmes[i] + **"\n"**);

}

}

**return** builder.toString();

}

**private class** HeaderListViewAdapter **extends** BaseAdapter{

@Override

**public int** getCount() {

**return mRecords**.size();

}

@Override

**public** Object getItem(**int** i) {

**if** (**mRecords**.size() == 0) **return "test"**;

**return mRecords**.get(i);

}

@Override

**public long** getItemId(**int** i) {

**return** getItem(i).hashCode();

}

@Override

**public** View getView(**int** i, View view, ViewGroup viewGroup) {

ViewHolder viewHolder;

**if** (view == **null**){

viewHolder = **new** ViewHolder();

view = LayoutInflater.*from*(getContext()).inflate(R.layout.***listitem_export_file_hearder*** ,**null**);

viewHolder.**numberTextView** = (TextView) view.findViewById(R.id.***tv_number***);

viewHolder.**nameTextView** = (TextView) view.findViewById(R.id.***tv_name***);

viewHolder.**weekNumberTextView** = (TextView) view.findViewById(R.id.***tv_week***);

viewHolder.**dateTextView** = (TextView) view.findViewById(R.id.***tv_date***);

viewHolder.**injectionNumberTextView** = (TextView) view.findViewById(R.id.***tv_injection_number***);

viewHolder.**splideLine** = view.findViewById(R.id.***v_splide_header_line***);

view.setTag(viewHolder);

}**else** {

viewHolder = (ViewHolder) view.getTag();

}

PatientDesensitizationRecordInfo patientDesensitizationRecordInfo = **mRecords**.get(i);

DesensitizationRecordInfo desensitizationRecordInfo = patientDesensitizationRecordInfo.getDesensitizationRecordInfo();

viewHolder.**numberTextView**.setText(patientDesensitizationRecordInfo.getPatientNumber());

viewHolder.**nameTextView**.setText(patientDesensitizationRecordInfo.getPatientName());

viewHolder.**weekNumberTextView**.setText(String.*valueOf*(desensitizationRecordInfo.getWeekNumber()));

viewHolder.**dateTextView**.setText(**dateFormat**.format(desensitizationRecordInfo.getInjectDate()*1000));

viewHolder.**injectionNumberTextView**.setText(String.*valueOf*(desensitizationRecordInfo.getInjectNumber()));

**if** (i+1 == getCount()){

viewHolder.**splideLine**.setVisibility(View.***GONE***);

}**else** {

viewHolder.**splideLine**.setVisibility(View.***VISIBLE***);

}

**return** view;

}

**private class** ViewHolder{

**private** TextView **numberTextView**;

**private** TextView **nameTextView**;

**private** TextView **weekNumberTextView**;

**private** TextView **dateTextView**;

**private** TextView **injectionNumberTextView**;

**private** android.view.View **splideLine**;

}

}

**private class** DesensitizationRecordListViewAdapter **extends** BaseAdapter{

**private int colorBlue** = getResources().getColor(R.color.***wordblue***);

**private int colorRed** = getResources().getColor(R.color.***wordred***);

@Override

**public int** getCount() {

**return mRecords**.size();

}

@Override

**public** Object getItem(**int** i) {

**if** (**mRecords**.size() == 0) **return "test"**;

**return mRecords**.get(i);

}

@Override

**public long** getItemId(**int** i) {

**return** getItem(i).hashCode();

}

@Override

**public** View getView(**int** i, View view, ViewGroup viewGroup) {

ViewHolder viewHolder;

**if** (view == **null**){

view = LayoutInflater.*from*(getContext()).inflate(R.layout.***listitem_export_file_desensization_record***,**null**);

viewHolder = **new** ViewHolder();

viewHolder.**healthStatusTextView** = (TextView) view.findViewById(R.id.***tv_health_status***);

viewHolder.**beforePEFTextView** = (TextView) view.findViewById(R.id.***tv_before_pef***);

viewHolder.**separateInjectionTextView** = (TextView) view.findViewById(R.id.***tv_inject_separate***);

viewHolder.**bottleNumberTextView** = (TextView) view.findViewById(R.id.***tv_bottle_number***);

viewHolder.**doseTextView** = (TextView) view.findViewById(R.id.***tv_dose***);

viewHolder.**injectionPartTextView** = (TextView) view.findViewById(R.id.***tv_injection_part***);

viewHolder.**injectionTimeTextView** = (TextView) view.findViewById(R.id.***tv_injection_time***);

viewHolder.**afterPEFTextView** = (TextView) view.findViewById(R.id.***tv_after_pef***);

viewHolder.**immedWhealSizeTextView** = (TextView) view.findViewById(R.id.***tv_wheal_size***);

viewHolder.**immediateReactionTextView** = (TextView) view.findViewById(R.id.***tv_immed_reaction***);

viewHolder.**delayReactionTextView** = (TextView) view.findViewById(R.id.***tv_delay_reaction***);

viewHolder.**responsiblePersionTextView** = (TextView) view.findViewById(R.id.***tv_responsible_person***);

viewHolder.**splideLine** = view.findViewById(R.id.***v_splide_line***);

view.setTag(viewHolder);

}**else** {

viewHolder = (ViewHolder) view.getTag();

}

PatientDesensitizationRecordInfo patientDesensitizationRecordInfo = **mRecords**.get(i);

DesensitizationRecordInfo desensitizationRecordInfo = patientDesensitizationRecordInfo.getDesensitizationRecordInfo();

**if** (desensitizationRecordInfo.getHealthStatus().length() == 0){

viewHolder.**healthStatusTextView**.setText(**"无"**);

}**else** {

viewHolder.**healthStatusTextView**.setText(desensitizationRecordInfo.getHealthStatus());

}

**if** (desensitizationRecordInfo.getBeforePEF() == 0){

viewHolder.**beforePEFTextView**.setText(**"-"**);

}**else** {

viewHolder.**beforePEFTextView**.setText(String.*valueOf*(desensitizationRecordInfo.getBeforePEF()));

}

viewHolder.**separateInjectionTextView**.setText(desensitizationRecordInfo.isInjectSeparate()? **"是"**:**"否"**); viewHolder.**bottleNumberTextView**.setText(String.*valueOf*(desensitizationRecordInfo.getBottleNumber()));

viewHolder.**doseTextView**.setText(desensitizationRecordInfo.getInjectDose());

**if**(desensitizationRecordInfo.getBottleNumber() != DesensitizationRecordInfo.*getAutoBottleNumber*(desensitizationRecordInfo.getInjectNumber()))

{

viewHolder.**bottleNumberTextView**.setTextColor(**colorRed**);

}**else**

{

viewHolder.**bottleNumberTextView**.setTextColor(Color.***BLACK***);

}

**if** (desensitizationRecordInfo.isInjectSeparate()){

viewHolder.**injectionPartTextView**.setText(

desensitizationRecordInfo.getInjectPart1() + **"/"** + desensitizationRecordInfo.getInjectPart2()

);

viewHolder.**injectionTimeTextView**.setText(

**timeFormat**.format(desensitizationRecordInfo.getInjectTime1() * 1000)

+ **"/"**

+ **timeFormat**.format(desensitizationRecordInfo.getInjectTime2() * 1000)

);

viewHolder.**separateInjectionTextView**.setTextColor(**colorRed**);

viewHolder.**doseTextView**.setTextColor(**colorRed**);

viewHolder.**injectionPartTextView**.setTextColor(**colorRed**);

viewHolder.**injectionTimeTextView**.setTextColor(**colorRed**);

}**else** {

viewHolder.**injectionPartTextView**.setText(desensitizationRecordInfo.getInjectPart1()); viewHolder.**injectionTimeTextView**.setText(**timeFormat**.format(desensitizationRecordInfo.getInjectTime1()));

viewHolder.**separateInjectionTextView**.setTextColor(Color.***BLACK***);

viewHolder.**doseTextView**.setTextColor(Color.***BLACK***);

viewHolder.**injectionPartTextView**.setTextColor(Color.***BLACK***);

viewHolder.**injectionTimeTextView**.setTextColor(Color.***BLACK***);

**if**(desensitizationRecordInfo.getInjectDoseTotal() != DesensitizationRecordInfo.*getAutoInjectDose*(desensitizationRecordInfo.getInjectNumber()))

{

viewHolder.**doseTextView**.setTextColor(**colorRed**);

}

}

**if**(desensitizationRecordInfo.getAfterPEF() == 0)

{

viewHolder.**afterPEFTextView**.setText(**"--"**);

}

**else** {

viewHolder.**afterPEFTextView**.setText(String.*valueOf*(desensitizationRecordInfo.getAfterPEF()));

}

**if**(desensitizationRecordInfo.getImmedWhealSize().length() == 0)

{

viewHolder.**immedWhealSizeTextView**.setText(**"无"**);

}

**else** {

viewHolder.**immedWhealSizeTextView**.setText(desensitizationRecordInfo.getImmedWhealSize());

} **if**(desensitizationRecordInfo.getImmedWhealSize().length() == 0)

{

viewHolder.**immedWhealSizeTextView**.setText(**"无"**);

}

**else** {

viewHolder.**immedWhealSizeTextView**.setText(desensitizationRecordInfo.getImmedWhealSize());

}

**if**(desensitizationRecordInfo.getImmedReaction().length() == 0)

{

viewHolder.**immediateReactionTextView**.setText(**"无"**);

}

**else**

{

viewHolder.**immediateReactionTextView**.setText(desensitizationRecordInfo.getImmedReaction());

}

**if**(desensitizationRecordInfo.getDelayReaction().length() == 0)

{

viewHolder.**delayReactionTextView**.setText(**"无"**);

}

**else** {

viewHolder.**delayReactionTextView**.setText(desensitizationRecordInfo.getDelayReaction());

}

viewHolder.**responsiblePersionTextView**.setText(**"--"**);

**if** (i+1 == getCount()){

viewHolder.**splideLine**.setVisibility(View.***GONE***);

}**else** {

viewHolder.**splideLine**.setVisibility(View.***VISIBLE***);

}

**return** view;

}

**private class** ViewHolder{

**private** TextView **healthStatusTextView**;

**private** TextView **beforePEFTextView**;

**private** TextView **separateInjectionTextView**;

**private** TextView **bottleNumberTextView**;

**private** TextView **doseTextView**;

**private** TextView **injectionPartTextView**;

**private** TextView **injectionTimeTextView**;

**private** TextView **immedWhealSizeTextView**;

**private** TextView **immediateReactionTextView**;

**private** TextView **afterPEFTextView**;

**private** TextView **delayReactionTextView**;

**private** TextView **responsiblePersionTextView**;

**private** View **splideLine**;

}

}

**public void** setOnExportClickListener(View.OnClickListener clickListener)

{

**mExportClickListener** = clickListener;

}

**public void** setCreatePDFFileListener(ExportFileFragmentManager.CreatePDFFileListener listener)

{

**mListener** = listener;

}

}

**package** com.elinkcare.ubreath.desensitization.account;

**import** android.app.Activity;

**import** android.content.Intent;

**import** android.os.Bundle;

**import** android.text.InputType;

**import** android.view.View;

**import** android.view.inputmethod.InputMethodManager;

**import** android.widget.Button;

**import** android.widget.ProgressBar;

**import** android.widget.Toast;

**import** com.elinkcare.ubreath.desensitization.MainActivity;

**import** com.elinkcare.ubreath.desensitization.R;

**import** com.elinkcare.ubreath.desensitization.core.ClientManager;

**import** com.elinkcare.ubreath.desensitization.core.CommonCallback;

**import** com.elinkcare.ubreath.desensitization.utils.CommonUtils;

**import** com.elinkcare.ubreath.desensitization.utils.StateCodeUtils;

**import** com.elinkcare.ubreath.desensitization.widget.DeleteEditText;

**public class** LoginActivity **extends** Activity {

**private** View **loginLayout**;

**private** DeleteEditText **accountEditText**;

**private** DeleteEditText **passwdEditText**;

**private** Button **loginButton**;

**private** ProgressBar **waittingProgressBar**;

@Override

**protected void** onCreate(Bundle savedInstanceState) {

**super**.onCreate(savedInstanceState);

setContentView(R.layout.***activity_login***);

initView();

initOnAction();

initData();

}

**private void** initView()

{

**loginLayout** = findViewById(R.id.***activity_login***);

**accountEditText** = (DeleteEditText) findViewById(R.id.***det_account***);

**passwdEditText** = (DeleteEditText) findViewById(R.id.***det_passwd***);

**loginButton** = (Button) findViewById(R.id.***bt_login***);

**waittingProgressBar** = (ProgressBar) findViewById(R.id.***pb_waitting***);

**accountEditText**.setHint(**"账号"**);

**accountEditText**.setInputType(InputType.***TYPE_CLASS_TEXT***);

**passwdEditText**.setHint(**"密码"**);

}

**private void** initOnAction()

{

**loginButton**.setOnClickListener(**new** View.OnClickListener() {

@Override

**public void** onClick(View view) {

login();

}

});

**loginLayout**.setOnClickListener(**new** View.OnClickListener() {

@Override

**public void** onClick(View view) {

hideKeyBoard();

}

});

}

**private void** initData()

{

}

**private synchronized void** login()

{

**if**(**waittingProgressBar**.getVisibility() == View.***VISIBLE***)

{

Toast.*makeText*(getBaseContext(), **"系统繁忙，请稍后再试"**, Toast.***LENGTH_SHORT***).show();

**return**;

}

String username = **accountEditText**.getText();

String password = **passwdEditText**.getText();

**if**(username == **null** || username.length() == 0)

{

Toast.*makeText*(getBaseContext(), **"请输入账号"**, Toast.***LENGTH_SHORT***).show();

**return**;

}

**if**(password == **null** || password.length() == 0)

{

Toast.*makeText*(getBaseContext(), **"请输入密码"**, Toast.***LENGTH_SHORT***).show();

**return**;

}

ClientManager.*getInstance*().login(username, CommonUtils.*generatePassword*(password), **new** CommonCallback() {

@Override

**public void** onSuccess() {

**waittingProgressBar**.setVisibility(View.***GONE***);

Toast.*makeText*(getBaseContext(), **"登录成功"**, Toast.***LENGTH_SHORT***).show();

Intent intent = **new** Intent(getBaseContext(), MainActivity.**class**);

startActivity(intent);

finish();

}

@Override

**public void** onError(String state) {

StateCodeUtils.*alert*(state, getBaseContext());

**waittingProgressBar**.setVisibility(View.***GONE***);

}

});

}

**private void** hideKeyBoard(){

((InputMethodManager) getSystemService(***INPUT_METHOD_SERVICE***))

.hideSoftInputFromWindow(getCurrentFocus()

.getWindowToken() InputMethodManager.***HIDE_NOT_ALWAYS***); } }

**package** com.elinkare.ubreath.desensitization.account;

**import** android.os.Bundle;

**import** android.support.annotation.Nullable;

**import** android.support.v4.app.Fragment;

**import** android.view.LayoutInflater;

**import** android.view.View;

**import** android.view.ViewGroup;

**import** android.widget.AdapterView;

**import** android.widget.BaseAdapter;

**import** android.widget.Button;

**import** android.widget.LinearLayout;

**import** android.widget.ListView;

**import** android.widget.ProgressBar;

**import** android.widget.TextView;

**import** android.widget.Toast;

**import** com.elinkcare.ubreath.desensitization.AccountManageFragment;

**import** com.elinkcare.ubreath.desensitization.R;

**import** com.elinkcare.ubreath.desensitization.core.ClientManager;

**import** com.elinkcare.ubreath.desensitization.core.CommonCallback;

**import** com.elinkcare.ubreath.desensitization.core.data.PatientRequestInfo;

**import** com.elinkcare.ubreath.desensitization.utils.StateCodeUtils;

**import** com.elinkcare.ubreath.desensitization.widget.popwindow.BasePop;

**import** com.elinkcare.ubreath.desensitization.widget.popwindow.MessageBoxPop;

**import** com.elinkcare.ubreath.desensitization.widget.popwindow.PatientsRequestInfoPop;

**import** java.text.SimpleDateFormat;

**import** java.util.ArrayList;

**import** java.util.Collections;

**import** java.util.Comparator;

**import** java.util.List;

*/***

** Created by ${Ray} on 2017/3/24.*

**/*

**public class** PatientsRequestFragment **extends** Fragment {

**private** View **mView**;

**private** View **backLayout**;

**private** LinearLayout **blankLayout**;

**private** ProgressBar **waitingProgressBar**;

**private** ListView **requestsListView**;

**private** RequestsListViewAdapter **mAdapter**;

**private** List<PatientRequestInfo> **mRequestList** = **new** ArrayList<>();

**private** PatientRequestInfo **mCurrentSelectedPatientRequestInfo**;

**private** MessageBoxPop **mConfirmSelectionPop**;

**private** PatientsRequestInfoPop **mPatientRequestInfoPop**;

**private** BasePop.OnPopSelectedListener **mConfirmPassListener**;

**private** BasePop.OnPopSelectedListener **mConfirmIgnoreListener**;

**private** AccountManageFragment.PatientsRequestNumberListener **mNumberListener**;

@Nullable

@Override

**public** View onCreateView(LayoutInflater inflater, @Nullable ViewGroup container, @Nullable Bundle savedInstanceState) {

**mView** = inflater.inflate(R.layout.***fragment_patient_request***,container , **false**);

initView();

initAction();

initDate();

initPop();

**return mView**;

}

**private void** initView(){

**backLayout** = **mView**.findViewById(R.id.***ll_back***);

**blankLayout** = (LinearLayout) **mView**.findViewById(R.id.***ll_blank***);

**waitingProgressBar** = (ProgressBar) **mView**.findViewById(R.id.***pb_waitting***);

**requestsListView** = (ListView) **mView**.findViewById(R.id.***lv_requests***);

**mAdapter** = **new** RequestsListViewAdapter();

**requestsListView**.setAdapter(**mAdapter**);

}

**private void** initAction(){

**backLayout**.setOnClickListener(**new** View.OnClickListener() {

@Override

**public void** onClick(View v) {

getActivity().finish();

}

});

**requestsListView**.setOnItemClickListener(**new** AdapterView.OnItemClickListener() {

@Override

**public void** onItemClick(AdapterView<?> parent, View view, **int** position, **long** id) {

**mCurrentSelectedPatientRequestInfo** = **mRequestList**.get(position);

**mPatientRequestInfoPop**.setPatientRequestInfo(**mCurrentSelectedPatientRequestInfo**);

**mPatientRequestInfoPop**.show(**null**,**mView**);

}

});

}

**private void** initDate(){

setUpView();

loadAllPatientsRequests();

}

**private void** initPop(){

**mConfirmSelectionPop** = **new** MessageBoxPop(getContext());

**mConfirmSelectionPop**.setTitle(**"患者申请"**);

**mConfirmSelectionPop**.setOkButtonContent(**"确定"**);

**mConfirmSelectionPop**.setOnCancelButtonContent(**"取消"**);

**mConfirmPassListener** = **new** BasePop.OnPopSelectedListener() {

@Override

**public void** onSelected(String selected) {

**int** type = 0;*// 0 - 接受（通过）申请;*

editPatientRequest(type);

}

@Override

**public void** onCancel() {

}

};

**mConfirmIgnoreListener** = **new** BasePop.OnPopSelectedListener() {

@Override

**public void** onSelected(String selected) {

**int** type = 1;*// 1 - 删除（忽略）申请；*

editPatientRequest(type);

}

@Override

**public void** onCancel() {

}

};

**mPatientRequestInfoPop** = **new** PatientsRequestInfoPop(getContext());

**mPatientRequestInfoPop**.setOnPopSelectedListener(**new** BasePop.OnPopSelectedListener() {

@Override

**public void** onSelected(String selected) {

showConfirmSelectionPop(selected);

}

@Override

**public void** onCancel() {

}

});

}

**private synchronized void** setUpView(){

List<PatientRequestInfo> mPatientRequestInfos = ClientManager.*getInstance*().getPatientRequestInfos();

**if** (mPatientRequestInfos != **null**){

**mRequestList**.clear();

**mRequestList**.addAll(mPatientRequestInfos);

}

**if** (**mRequestList**.size() == 0){

**blankLayout**.setVisibility(View.***VISIBLE***);

**requestsListView**.setVisibility(View.***GONE***);

}**else** {

**blankLayout**.setVisibility(View.***GONE***);

**requestsListView**.setVisibility(View.***VISIBLE***);

}

Collections.*sort*(**mRequestList**, **new** Comparator<PatientRequestInfo>() {

@Override

**public int** compare(PatientRequestInfo lhs, PatientRequestInfo rhs) {

**int** leftStatus = lhs.getRespondStatus();

**int** rightStatus = rhs.getRespondStatus();

**if** (leftStatus == rightStatus){

**long** leftTime = lhs.getRequestTime();

**long** rightTime = rhs.getRequestTime();

**if** (leftTime > rightTime){

**return** -1;

}

**if** (leftTime < rightTime){

**return** 1;

}

**return** 0;

}

**if** (leftStatus > rightStatus){

**return** 1;

}

**if** (leftStatus < rightStatus){

**return** -1;

}

**return** 0;

}

});

**mAdapter**.notifyDataSetChanged();

**if** (**mNumberListener** != **null**){

**mNumberListener**.needComformNumber(getNeedConfirmCount());

}

}

**private void** loadAllPatientsRequests(){

**if** (**waitingProgressBar**.getVisibility() == View.***VISIBLE***){

Toast.*makeText*(getContext(), **"系统繁忙"**, Toast.***LENGTH_SHORT***).show();

**return**;

}

**waitingProgressBar**.setVisibility(View.***VISIBLE***);

ClientManager.*getInstance*().loadAllPatientRequests(**new** CommonCallback() {

@Override

**public void** onSuccess() {

**waitingProgressBar**.setVisibility(View.***GONE***);

setUpView();

}

@Override

**public void** onError(String state) {

**waitingProgressBar**.setVisibility(View.***GONE***);

StateCodeUtils.*alert*(state,getContext());

}

});

}

**private void** editPatientRequest(**int** type){

**if** (**waitingProgressBar**.getVisibility() == View.***VISIBLE***){

Toast.*makeText*(getContext(), **"系统繁忙，请稍后"**, Toast.***LENGTH_SHORT***).show();

**return**;

}

**waitingProgressBar**.setVisibility(View.***VISIBLE***);

ClientManager.*getInstance*().editPatientRequest(**mCurrentSelectedPatientRequestInfo**.getId(), type, **new** CommonCallback() {

@Override

**public void** onSuccess() {

**waitingProgressBar**.setVisibility(View.***GONE***);

loadAllPatientsRequests();

}

@Override

**public void** onError(String state) {

**waitingProgressBar**.setVisibility(View.***GONE***);

StateCodeUtils.*alert*(state,getContext()); } }); }

**private int** getNeedConfirmCount(){

**int** needConfirmCount = 0;

**for** (**int** i = 0; i < **mRequestList**.size();i++){

**if** (**mRequestList**.get(i).getRespondStatus() == 0){

needConfirmCount ++;

}

}

**return** needConfirmCount;

}

**private void** showConfirmSelectionPop(String type){

**if** (type.equals(**"ignore"**)){

**mConfirmSelectionPop**.setMessage(**"你确定忽略"**+ **"[ "**+**mCurrentSelectedPatientRequestInfo**.getName()+**" ]"**+ **"的申请吗?"**);

**mConfirmSelectionPop**.setOnPopSelectedListener(**mConfirmIgnoreListener**);

}

**if** (type.equals(**"pass"**)){

**mConfirmSelectionPop**.setMessage(**"你确定要通过"**+ **"[ "**+**mCurrentSelectedPatientRequestInfo**.getName()+**" ]"**+**"的申请吗?"**);

**mConfirmSelectionPop**.setOnPopSelectedListener(**mConfirmPassListener**);

}

**mConfirmSelectionPop**.show(**null**,**mView**);

}

**private class** RequestsListViewAdapter **extends** BaseAdapter{

**private** SimpleDateFormat **mFormat** = **new** SimpleDateFormat(**"yyyy-MM-dd"**);

@Override

**public int** getCount() {

**return mRequestList**.size();

}

@Override

**public** Object getItem(**int** position) {

**if** (**mRequestList**.size() != 0) **return mRequestList**.get(position);

**return "test"**;

}

@Override

**public long** getItemId(**int** position) {

**return** getItem(position).hashCode();

}

@Override

**public** View getView(**int** position, View convertView,ViewGroup parent) {

ViewHolder viewHolder;

**if** (convertView == **null**){

convertView = LayoutInflater.*from*(getContext()).inflate(R.layout.***listitem_patient_request***,**null**);

viewHolder = **new** ViewHolder();

viewHolder.**nameTextView** = (TextView) convertView.findViewById(R.id.***tv_name***);

viewHolder.**birthTextView** = (TextView) convertView.findViewById(R.id.***tv_birth***);

viewHolder.**telTextView** = (TextView) convertView.findViewById(R.id.***tv_contact_tel***);

viewHolder.**illnessTextView** = (TextView) convertView.findViewById(R.id.***tv_illness***);

viewHolder.**handleNeedConfirmLayout** = (LinearLayout) convertView.findViewById(R.id.***ll_handle_need_confirm***);

viewHolder.**ignoreButton** = (Button) convertView.findViewById(R.id.***bt_ignore***);

viewHolder.**passButton** = (Button) convertView.findViewById(R.id.***bt_pass***);

viewHolder.**handledPassTextView** = (TextView) convertView.findViewById(R.id.***tv_handle_pass***);

viewHolder.**splitView** = convertView.findViewById(R.id.***v_split***);

convertView.setTag(viewHolder);

}**else** {

viewHolder = (ViewHolder) convertView.getTag();

}

**final** PatientRequestInfo requestInfo = **mRequestList**.get(position);

viewHolder.**nameTextView**.setText(requestInfo.getName());

viewHolder.**birthTextView**.setText(**mFormat**.format(requestInfo.getBirth()*1000));

viewHolder.**telTextView**.setText(requestInfo.getPhone());

viewHolder.**illnessTextView**.setText(requestInfo.getIllness());

**switch** (requestInfo.getRespondStatus()){

**case** 0:

viewHolder.**handleNeedConfirmLayout**.setVisibility(View.***VISIBLE***);

viewHolder.**handledPassTextView**.setVisibility(View.***GONE***);

**break**;

**case** 1:

viewHolder.**handleNeedConfirmLayout**.setVisibility(View.***GONE***);

viewHolder.**handledPassTextView**.setVisibility(View.***VISIBLE***);

**break**;

}

viewHolder.**ignoreButton**.setOnClickListener(**new** View.OnClickListener() {

@Override

**public void** onClick(View v) {

**mCurrentSelectedPatientRequestInfo** = requestInfo;

showConfirmSelectionPop(**"ignore"**);

}

});

viewHolder.**passButton**.setOnClickListener(**new** View.OnClickListener() {

@Override

**public void** onClick(View v) {

**mCurrentSelectedPatientRequestInfo** = requestInfo;

showConfirmSelectionPop(**"pass"**);

}

});

**if** (getCount() == position + 1){

viewHolder.**splitView**.setVisibility(View.***GONE***);

}**else** {

viewHolder.**splitView**.setVisibility(View.***VISIBLE***);

}

**return** convertView;

}

**private class** ViewHolder{

**private** TextView **nameTextView**;

**private** TextView **birthTextView**;

**private** TextView **telTextView**;

**private** TextView **illnessTextView**;

**private** LinearLayout **handleNeedConfirmLayout**;

**private** Button **ignoreButton**;

**private** Button **passButton**;

**private** TextView **handledPassTextView**;

**private** View **splitView**;

}

}

**public void** setPatientsRequestNumberListener(AccountManageFragment.PatientsRequestNumberListener listener){

**mNumberListener** = listener;

}

}

**package** com.elinkcare.ubreath.desensitization.account;

**import** android.os.Bundle;

**import** android.support.v4.app.Fragment;

**import** android.view.LayoutInflater;

**import** android.view.View;

**import** android.view.ViewGroup;

**import** android.widget.AbsListView;

**import** android.widget.AdapterView;

**import** android.widget.BaseAdapter;

**import** android.widget.ImageView;

**import** android.widget.ListView;

**import** android.widget.TextView;

**import** com.elinkcare.ubreath.desensitization.R;

**import** com.elinkcare.ubreath.desensitization.core.ClientManager;

**import** com.elinkcare.ubreath.desensitization.core.CommonCallback;

**import** com.elinkcare.ubreath.desensitization.core.data.PatientInfo;

**import** com.elinkcare.ubreath.desensitization.core.data.PaymentInfo;

**import** com.elinkcare.ubreath.desensitization.utils.CommonUtils;

**import** com.elinkcare.ubreath.desensitization.utils.StateCodeUtils;

**import** java.text.SimpleDateFormat;

**import** java.util.ArrayList;

**import** java.util.Collections;

**import** java.util.Comparator;

**import** java.util.List;

*/***

** A simple {****@link*** *Fragment} subclass.*

**/*

**public class** PaymentManageFragment **extends** Fragment {

**private** View **mView**;

**private** View **backLayout**;

**private** TextView **needPayPatientNumberTextView**;

**private** TextView **paidPatientNumberTextView**;

**private** ListView **patientListView**;

**private** PatientListViewAdapter **mPatientAdapter**;

**private** List<PaymentInfo> **mPaymentRecordList** = **new** ArrayList<>();

**public** PaymentManageFragment() {

*// Required empty public constructor*

}

@Override

**public** View onCreateView(LayoutInflater inflater, ViewGroup container,

Bundle savedInstanceState) {

**mView** = inflater.inflate(R.layout.***fragment_payment_manage***, container, **false**);

initView();

initAction();

initData();

**return mView**;

}

**private void** initView(){

**backLayout** = **mView**.findViewById(R.id.***ll_back***);

**needPayPatientNumberTextView** = (TextView) **mView**.findViewById(R.id.***tv_need_payment_number***);

**paidPatientNumberTextView** = (TextView) **mView**.findViewById(R.id.***tv_paid_payment_number***);

**patientListView** = (ListView) **mView**.findViewById(R.id.***lv_all_patients***);

**mPatientAdapter** = **new** PatientListViewAdapter();

**patientListView**.setAdapter(**mPatientAdapter**);

}

**private void** addFootView(ListView listView){

**if** (listView == **null**) **return**;

View view = **new** View(getContext());

AbsListView.LayoutParams params = **new** AbsListView.LayoutParams(ViewGroup.LayoutParams.***MATCH_PARENT***, (**int**) CommonUtils.*dp2Px*(getContext(),50));

view.setLayoutParams(params);

view.setBackgroundColor(getResources().getColor(R.color.***background***));

listView.addFooterView(view);

}

**private void** initAction(){

**backLayout**.setOnClickListener(**new** View.OnClickListener() {

@Override

**public void** onClick(View view) {

getActivity().finish();

}

});

**patientListView**.setOnItemClickListener(**new** AdapterView.OnItemClickListener() {

@Override

**public void** onItemClick(AdapterView<?> adapterView, View view, **int** i, **long** l) {

String patientId = **mPaymentRecordList**.get(i).getPatientId();

PatientInfo patientInfo = ClientManager.*getInstance*().getPatient(patientId);

**if** (patientInfo != **null**){

ClientManager.*getInstance*().setCurrentPatient(patientInfo);

getActivity().finish();

}**else** {} } }); }

**private void** initData(){

refreshPaymentListView();

loadAllPaymentRecords();

}

**private void** loadAllPaymentRecords(){

ClientManager.*getInstance*().loadAllPatientsPaymentRecords(**new** CommonCallback() {

@Override

**public void** onSuccess() {

refreshPaymentListView();

}

@Override

**public void** onError(String state) {

StateCodeUtils.*alert*(state,getContext());

}

});

}

**private synchronized void** refreshPaymentListView(){

List<PaymentInfo> payments = ClientManager.*getInstance*().getAllPaymentRecords();

**if** (payments != **null**){

**mPaymentRecordList**.clear();

**mPaymentRecordList**.addAll(payments);

sortPaymentRecordInfoByPaymentTime(**mPaymentRecordList**);

}

**mPatientAdapter**.notifyDataSetChanged();

**private void** sortPaymentRecordInfoByPaymentTime(List<PaymentInfo> paymentInfos){

**if** (paymentInfos == **null**) **return**;

Collections.*sort*(paymentInfos, **new** Comparator<PaymentInfo>() {

@Override

**public int** compare(PaymentInfo lhs, PaymentInfo rhs) {

**if** (lhs.getPaymentTime() > rhs.getPaymentTime()){

**return** -1;

}

**if** (lhs.getPaymentTime() < rhs.getPaymentTime()){

**return** 1;

}

**return** 0;

}

});

}

**private class** PatientListViewAdapter **extends** BaseAdapter{

**private** SimpleDateFormat **mFormat** = **new** SimpleDateFormat(**"yyyy-MM-dd"**);

@Override

**public int** getCount() {

**return mPaymentRecordList**.size();

}

@Override

**public** Object getItem(**int** i) {

**if** (**mPaymentRecordList**.size() == 0){

**return "test"**;

}

**return mPaymentRecordList**.get(i);

}

@Override

**public long** getItemId(**int** i) {

**return** getItem(i).hashCode();

}

@Override

**public** View getView(**int** i, View view, ViewGroup viewGroup) {

ViewHolder viewHolder;

**if** (view == **null**){

view = LayoutInflater.*from*(getContext()).inflate(R.layout.***listitem_payment_manage***,**null**);

viewHolder = **new** ViewHolder();

viewHolder.**orderImageView** = (ImageView) view.findViewById(R.id.***iv_order***);

viewHolder.**orderTextView** = (TextView) view.findViewById(R.id.***tv_order***);

viewHolder.**nameTextView** = (TextView) view.findViewById(R.id.***tv_name***);

viewHolder.**numberTextView** = (TextView) view.findViewById(R.id.***tv_number***);

viewHolder.**paymentTimeTextView** = (TextView) view.findViewById(R.id.***tv_payment_time***);

viewHolder.**paymentTypeTextView** = (TextView) view.findViewById(R.id.***tv_payment_type***);

viewHolder.**predictInjectionTimeTextView** = (TextView) view.findViewById(R.id.***tv_predict_injection_time***);

viewHolder.**remainInjectionCountTextView** = (TextView) view.findViewById(R.id.***tv_injection_number***);

viewHolder.**remainMedicineCountTextView** = (TextView) view.findViewById(R.id.***tv_medicine_number***);

viewHolder.**spliteLine** = view.findViewById(R.id.***v_splide_line***);

view.setTag(viewHolder);

}**else** {

viewHolder = (ViewHolder) view.getTag();

}

PaymentInfo paymentInfo = **mPaymentRecordList**.get(i);

viewHolder.**nameTextView**.setText(paymentInfo.getPatientName());

viewHolder.**numberTextView**.setText(paymentInfo.getPatientNumber());

**switch** (paymentInfo.getType()){

**case** 1:

viewHolder.**paymentTypeTextView**.setText(**"药品费["**+paymentInfo.getTimes()+**"]"**);

**break**;

**case** 2:

viewHolder.**paymentTypeTextView**.setText(**"治疗费["**+paymentInfo.getTimes()+**"]"**);

**break**;

}

**if** (paymentInfo.getPaymentTime() == 0){

viewHolder.**paymentTimeTextView**.setText(**"---"**);

}**else** {

viewHolder.**paymentTimeTextView**.setText(**mFormat**.format(paymentInfo.getPaymentTime()*1000));}

viewHolder.**orderImageView**.setVisibility(View.***INVISIBLE***);

viewHolder.**orderTextView**.setText(i+1+**""**);

viewHolder.**predictInjectionTimeTextView**.setText(**"--"**);

viewHolder.**remainInjectionCountTextView**.setText(**"--"**);

viewHolder.**remainMedicineCountTextView**.setText(**"--"**);

**if** (i+1 == getCount()){

viewHolder.**spliteLine** .setVisibility(View.***INVISIBLE***);

}**else** {

viewHolder.**spliteLine**.setVisibility(View.***VISIBLE***);

}

**return** view;

}

**private class** ViewHolder{

**private** ImageView **orderImageView**;

**private** TextView **orderTextView**;

**private** TextView **nameTextView**;

**private** TextView **numberTextView**;

**private** TextView **paymentTimeTextView**;

**private** TextView **paymentTypeTextView**;

**private** TextView **predictInjectionTimeTextView**;

**private** TextView **remainInjectionCountTextView**;

**private** TextView **remainMedicineCountTextView**;

**private** View **spliteLine**;

}

}

}

**package** com.elinkcare.ubreath.desensitization.account;

**import** android.os.Bundle;

**import** android.support.v4.app.Fragment;

**import** android.view.LayoutInflater;

**import** android.view.View;

**import** android.view.ViewGroup;

**import** android.widget.AbsListView;

**import** android.widget.AdapterView;

**import** android.widget.BaseAdapter;

**import** android.widget.Button;

**import** android.widget.ListView;

**import** android.widget.ProgressBar;

**import** android.widget.TextView;

**import** android.widget.Toast;

**import** com.elinkcare.ubreath.desensitization.R;

**import** com.elinkcare.ubreath.desensitization.core.ClientManager;

**import** com.elinkcare.ubreath.desensitization.core.CommonCallback;

**import** com.elinkcare.ubreath.desensitization.core.data.DesensitizationRecordInfo;

**import** com.elinkcare.ubreath.desensitization.core.data.PatientInfo;

**import** com.elinkcare.ubreath.desensitization.utils.CommonUtils;

**import** java.text.SimpleDateFormat;

**import** java.util.ArrayList;

**import** java.util.Collections;

**import** java.util.Comparator;

**import** java.util.List;

*/***

** A simple {****@link*** *Fragment} subclass.*

**/*

**public class** InjectionListFragment **extends** Fragment {

**private** View **mView**;

**private** View **backLayout**;

**private** Button **preWeekButton**;

**private** Button **nextWeekButton**;

**private** TextView **timeTextView**;

**private** TextView **patientCountTextView**;

**private** TextView **injectedPatientCountTextView**;

**private** ListView **patientListView**;

**private** ProgressBar **waitingProgressBar**;

**private** List<DesensitizationRecordInfo> **mInjectedRecords** = **new** ArrayList<>();

**private** List<DesensitizationRecordInfo> **mUninjectedRecords** = **new** ArrayList<>();

**private** InjectRecordsAdapter **mAdapter**;

**private** SimpleDateFormat **mDateFormat** = **new** SimpleDateFormat(**"yyyy-MM-dd"**);

**private long mWeekStartTime**;

**private int mWeekPosition** = 0;

**private boolean mIsThisWeek** = **false**;

**private int mGrayColor**;

**private int mBlueColor**;

**public** InjectionListFragment() {

*// Required empty public constructor*

}

@Override

**public** View onCreateView(LayoutInflater inflater, ViewGroup container,

Bundle savedInstanceState) {

**mView** = inflater.inflate(R.layout.***fragment_injection_list***, container, **false**);

initView();

initOnAction();

initData();

**return mView**;

}

**private void** initView() {

**backLayout** = **mView**.findViewById(R.id.***ll_back***);

**timeTextView** = (TextView) **mView**.findViewById(R.id.***tv_time***);

**preWeekButton** = (Button) **mView**.findViewById(R.id.***bt_pre_week***);

**nextWeekButton** = (Button) **mView**.findViewById(R.id.***bt_next_week***);

**patientCountTextView** = (TextView) **mView**.findViewById(R.id.***tv_injection_patient_count***);

**injectedPatientCountTextView** = (TextView) **mView**.findViewById(R.id.***tv_injected_patient_count***);

**waitingProgressBar** = (ProgressBar) **mView**.findViewById(R.id.***pb_waitting***);

**patientListView** = (ListView) **mView**.findViewById(R.id.***lv_injection_list***);

addFootView(**patientListView**);

**mAdapter** = **new** InjectRecordsAdapter();

**patientListView**.setAdapter(**mAdapter**);

}

**private void** initOnAction() {

**backLayout**.setOnClickListener(**new** View.OnClickListener() {

@Override

**public void** onClick(View view) {

getActivity().finish();

}

});

**preWeekButton**.setOnClickListener(**new** View.OnClickListener() {

@Override

**public void** onClick(View view) {

**if** (**mWeekPosition** == 2) {

**nextWeekButton**.setTextColor(**mBlueColor**); **nextWeekButton**.setBackgroundResource(R.drawable.***shape_rround_blue_stroke_r5***);

}

**mWeekStartTime** -= 7 * 24 * 3600 * 1000;

**mWeekPosition**--;

refreshInjectList();

loadInjectRecords(); } });

**nextWeekButton**.setOnClickListener(**new** View.OnClickListener() {

@Override

**public void** onClick(View view) {

**if** (**mWeekPosition** == 3) {

Toast.*makeText*(getContext(), **"只能查看未来三周的打针信息"**, Toast.***LENGTH_SHORT***).show();

**return**;

}

**mWeekStartTime** += 7 * 24 * 3600 * 1000;

**mWeekPosition**++;

refreshInjectList();

loadInjectRecords();

**if** (**mWeekPosition** == 3) {

**nextWeekButton**.setTextColor(**mGrayColor**); **nextWeekButton**.setBackgroundResource(R.drawable.***shape_rround_gray_stroke_r5***); } });

**patientListView**.setOnItemClickListener(**new** AdapterView.OnItemClickListener() {

@Override

**public void** onItemClick(AdapterView<?> adapterView, View view, **int** i, **long** l){

**if** ( i == (**mInjectedRecords**.size() + **mUninjectedRecords**.size())){

**return**;}

String patientId = i< **mInjectedRecords**.size()? **mInjectedRecords**.get(i).getPatientId():**mUninjectedRecords**.get(i - **mInjectedRecords**.size()).getPatientId();

PatientInfo patientInfo = ClientManager.*getInstance*().getPatient(patientId);

**if** (patientInfo != **null**){

ClientManager.*getInstance*().setCurrentPatient(patientInfo);

getActivity().finish(); } }});}

**private void** initData() {

**mGrayColor** = getResources().getColor(R.color.***wordgray***);

**mBlueColor** = getResources().getColor(R.color.***wordblue***);

**mWeekStartTime** = CommonUtils.*getWeekStart*(System.*currentTimeMillis*());

refreshInjectList();

loadInjectRecords();

}

**private void** addFootView(ListView listView) {

**if** (listView == **null**) **return**;

View view = **new** View(getContext());

AbsListView.LayoutParams params = **new** AbsListView.LayoutParams(ViewGroup.LayoutParams.***MATCH_PARENT***, (**int**) CommonUtils.*dp2Px*(getContext(), 50));

view.setLayoutParams(params);

listView.addFooterView(view);

}

**private synchronized void** refreshInjectList() {

**long** weekEndTime = **mWeekStartTime** + 7 * 24 * 3600 * 1000 - 1;

**if** (**mWeekPosition** == 0) {

**timeTextView**.setText(**"本周"**);

**mIsThisWeek** = **true**;

} **else** {

**timeTextView**.setText(**mDateFormat**.format(**mWeekStartTime**) + **"至"** + **mDateFormat**.format(weekEndTime));

**mIsThisWeek** = **false**;

}

List<DesensitizationRecordInfo> injectedRecords = ClientManager.*getInstance*().getInjectedRecords(**mWeekStartTime** / 1000);

List<DesensitizationRecordInfo> uninjectedRecords = ClientManager.*getInstance*().getUninjectedRecords(**mWeekStartTime** / 1000);

**if** (**mInjectedRecords** != injectedRecords) {

**mInjectedRecords**.clear();

**mInjectedRecords**.addAll(injectedRecords);

}

**if** (**mUninjectedRecords** != uninjectedRecords) {

**mUninjectedRecords**.clear();

**mUninjectedRecords**.addAll(uninjectedRecords);

}

Collections.*sort*(**mInjectedRecords**, **new** Comparator<DesensitizationRecordInfo>() {

@Override

**public int** compare(DesensitizationRecordInfo lhs, DesensitizationRecordInfo rhs{

**long** leftInjectionTime = lhs.getInjectDate()/( 24 * 3600) * (24 * 3600);

**long** rightInjectionTime = rhs.getInjectDate()/( 24 * 3600) * (24 * 3600);

**if** (lhs.isInjectSeparate()){

leftInjectionTime = lhs.getInjectTime2()%(24 * 3600) + leftInjectionTime;}**else** {

leftInjectionTime = lhs.getInjectTime1()%(24 * 3600) + leftInjectionTime; }

**if** (rhs.isInjectSeparate()){

rightInjectionTime = rhs.getInjectTime2()%(24 * 3600) + rightInjectionTime;

}**else** {

rightInjectionTime = rhs.getInjectTime1()%(24 * 3600) + rightInjectionTime;

}

**if** (leftInjectionTime > rightInjectionTime) **return** -1;

**if** (leftInjectionTime < rightInjectionTime) **return** 1;

**return** 0; } });

Collections.*sort*(**mUninjectedRecords**, **new** Comparator<DesensitizationRecordInfo>() {

@Override

**public int** compare(DesensitizationRecordInfo lhs, DesensitizationRecordInfo rhs) {

**if** (lhs.getInjectDate() > rhs.getInjectDate()) **return** -1;

**if** (lhs.getInjectDate() < rhs.getInjectDate()) **return** 1;

**return** 0; } });

**mAdapter**.notifyDataSetChanged();

**patientCountTextView**.setText(String.*valueOf*(**mAdapter**.getCount()));

**injectedPatientCountTextView**.setText(String.*valueOf*(**mInjectedRecords**.size()));

}

**private class** InjectRecordsAdapter **extends** BaseAdapter {

**private** SimpleDateFormat **mDateFormat** = **new** SimpleDateFormat(**"MM-dd"**);

@Override

**public int** getCount() {

**return mInjectedRecords**.size() + **mUninjectedRecords**.size();

}

@Override

**public** Object getItem(**int** position) {

**if** (position < **mInjectedRecords**.size()) {

**return mInjectedRecords**.get(position);

} **else** {

**return mUninjectedRecords**.get(position - **mInjectedRecords**.size());

}

}

@Override

**public long** getItemId(**int** position) {

**return** getItem(position).hashCode();

}

@Override

**public** View getView(**int** position, View convertView, ViewGroup parent) {

ViewHolder viewHolder;

**if** (convertView == **null**) {

viewHolder = **new** ViewHolder();

convertView = LayoutInflater.*from*(getContext()).inflate(R.layout.***listitem_injection_record***,**null**);

viewHolder.**nameTextView** = (TextView) convertView.findViewById(R.id.***tv_name***);

viewHolder.**numberTextView** = (TextView) convertView.findViewById(R.id.***tv_number***);

viewHolder.**injectionTimeTextView** = (TextView) convertView.findViewById(R.id.***tv_injection_time***);

viewHolder.**injectionNumberTextView** = (TextView) convertView.findViewById(R.id.***tv_injection_number***);

viewHolder.**immedResponseTextView** = (TextView) convertView.findViewById(R.id.***tv_immed_response***);

viewHolder.**delayResponseTextView** = (TextView) convertView.findViewById(R.id.***tv_delay_response***);

viewHolder.**spliteLine** = convertView.findViewById(R.id.***v_splide_line***);

convertView.setTag(viewHolder);

} **else** {

viewHolder = (ViewHolder) convertView.getTag();

}

DesensitizationRecordInfo record = (DesensitizationRecordInfo) getItem(position);

PatientInfo patient = ClientManager.*getInstance*().getPatient(record.getPatientId());

**if** (patient != **null**){

viewHolder.**nameTextView**.setText(patient.getName());

viewHolder.**numberTextView**.setText(patient.getNumber());

}**else** {

viewHolder.**nameTextView**.setText(**"--"**);

viewHolder.**numberTextView**.setText(**"--"**);

}

**if** (position < **mInjectedRecords**.size()) {

viewHolder.**injectionTimeTextView**.setText(**mDateFormat**.format(record.getInjectDate()1000));

viewHolder.**injectionNumberTextView**.setText(String.*valueOf*(record.getInjectNumber()));

**if** (record.getImmedReaction().length() == 0) {

viewHolder.**immedResponseTextView**.setText(**"无"**);

} **else** {

viewHolder.**immedResponseTextView**.setText(record.getImmedReaction());

}

**if** (record.getDelayReaction().length() == 0) {

viewHolder.**delayResponseTextView**.setText(**"无"**);

} **else** {

iewHolder.**delayResponseTextView**.setText(record.getDelayReaction());

}

} **else** {

viewHolder.**injectionTimeTextView**.setText(**"未打针"**);

viewHolder.**injectionNumberTextView**.setText(**"--"**);

viewHolder.**immedResponseTextView**.setText(**"--"**);

viewHolder.**delayResponseTextView**.setText(**"--"**);

} **return** convertView;}

**private class** ViewHolder {

**private** TextView **nameTextView**;

**private** TextView **numberTextView**;

**private** TextView **injectionTimeTextView**;

**private** TextView **injectionNumberTextView**;

**private** TextView **immedResponseTextView**;

**private** TextView **delayResponseTextView**; }

**private void** loadInjectRecords() {

**if** (**waitingProgressBar**.getVisibility() == View.***VISIBLE***){

Toast.*makeText*(getContext(), **"系统繁忙"**, Toast.***LENGTH_SHORT***).show();

**return**;

}

**waitingProgressBar**.setVisibility(View.***VISIBLE***);

ClientManager.*getInstance*().loadAllWeekInjectRecords(**mWeekStartTime** / 1000, **mIsThisWeek**, **new** CommonCallback() {

@Override

**public void** onSuccess() {

**waitingProgressBar**.setVisibility(View.***GONE***);

refreshInjectList();

}

@Override

**public void** onError(String state) {

**waitingProgressBar**.setVisibility(View.***GONE***);} }); }}

**package** com.elinkcare.ubreath.desensitization.adapter;

**import** android.content.Context;

**import** android.view.LayoutInflater;

**import** android.view.View;

**import** android.view.ViewGroup;

**import** android.widget.BaseAdapter;

**import** android.widget.TextView;

**import** com.elinkcare.ubreath.desensitization.R;

**import** com.elinkcare.ubreath.desensitization.core.data.PfmDataRecordInfo;

**import** java.text.SimpleDateFormat;

**import** java.util.List;

*/***

** Created by Jason on 17/2/10.*

**/*

**public class** BriefPfmRecordsAdapter **extends** BaseAdapter{

**private** Context **mContext**;

**private** List<PfmDataRecordInfo> **mRecords**;

**private** SimpleDateFormat **mTimeFormat** = **new** SimpleDateFormat(**"HH:mm"**);

**public** BriefPfmRecordsAdapter(List<PfmDataRecordInfo> records, Context context)

{**this**.**mContext** = context;

**this**.**mRecords** = records; }

@Override

**public int** getCount() {

**if**(**mRecords** == **null**)**return** 0;

**return mRecords**.size() > 4 ? 4 : **mRecords**.size();

}

@Override

**public** Object getItem(**int** position) {

**return mRecords**.get(position);

}

@Override

**public long** getItemId(**int** position) {

**return** getItem(position).hashCode();

@Override

**public** View getView(**int** position, View convertView, ViewGroup parent) {

ViewHolder viewHolder;

**if**(convertView == **null**)

{

convertView = LayoutInflater.*from*(**mContext**).inflate(R.layout.***listitem_pfm_record_brief***, **null**);

viewHolder = **new** ViewHolder();

viewHolder.**timeTextView** = (TextView) convertView.findViewById(R.id.***tv_time***);

viewHolder.**pefTextView** = (TextView) convertView.findViewById(R.id.***tv_pef***);

viewHolder.**fev1TextView** = (TextView) convertView.findViewById(R.id.***tv_fev1***);

viewHolder.**fvcTextView** = (TextView) convertView.findViewById(R.id.***tv_fvc***);

convertView.setTag(viewHolder);

} **else**

{

viewHolder = (ViewHolder) convertView.getTag();

}

PfmDataRecordInfo record = **mRecords**.get(position);

viewHolder.**timeTextView**.setText(**mTimeFormat**.format(record.getTime() * 1000));

viewHolder.**pefTextView**.setText(String.*valueOf*(record.getPEF()));

viewHolder.**fev1TextView**.setText(String.*format*(**"%1.2f"**, 0.01f * record.getFEV1()));

viewHolder.**fvcTextView**.setText(String.*format*(**"%1.2f"**, 0.01f * record.getFVC()));

**return** convertView; }

**private class** ViewHolder

{

**public** TextView **timeTextView**;

**public** TextView **pefTextView**;

**public** TextView **fev1TextView**;

**public** TextView **fvcTextView**;

}}

**package** com.elinkcare.ubreath.desensitization.adapter;

**import** android.content.Context;

**import** android.view.LayoutInflater;

**import** android.view.View;

**import** android.view.ViewGroup;

**import** android.widget.BaseAdapter;

**import** android.widget.TextView;

**import** com.elinkcare.ubreath.desensitization.R;

**import** com.elinkcare.ubreath.desensitization.core.data.DesensitizationRecordInfo;

**import** java.text.SimpleDateFormat;

**import** java.util.Calendar;

**import** java.util.List;

*/***

** Created by yejiasheng on 17/2/17.*

**/*

**public class** DesensitizationHeaderAdapter **extends** BaseAdapter{

**private final int TYPE_COUNT** = 2;

**private final int TYPE_CURRENT** = 0;

**private final int TYPE_HISTORY** = 1;

**private long mTodayStart**;

**private long mTodayEnd**;

**private** Context **mContext**;

**private** List<DesensitizationRecordInfo> **mRecords**;

**private** SimpleDateFormat **mDateFormat** = **new** SimpleDateFormat(**"MM-dd"**);

**public** DesensitizationHeaderAdapter(Context context, List<DesensitizationRecordInfo> records)

{

**this**.**mContext** = context;

**this**.**mRecords** = records;

}

@Override

**public void** notifyDataSetChanged() {

Calendar cal = Calendar.*getInstance*();

cal.set(Calendar.***HOUR_OF_DAY***, 0);

cal.set(Calendar.***MINUTE***, 0);

cal.set(Calendar.***SECOND***, 0);

**mTodayStart** = cal.getTimeInMillis() / 1000;

**mTodayEnd** = **mTodayStart** + 24 * 3600;

**super**.notifyDataSetChanged();

}

@Override

**public int** getCount() {

**return mRecords** == **null** ? 0 : **mRecords**.size();

}

@Override

**public** Object getItem(**int** position) {

**return "test"**;

}

@Override

**public long** getItemId(**int** position) {

**return** getItem(position).hashCode();

}

@Override

**public int** getItemViewType(**int** position) {

DesensitizationRecordInfo record = **mRecords**.get(position);

**if**(record.isCurrentRecord())

{

**return TYPE_CURRENT**;

}

**else**

{

**return TYPE_HISTORY**;

}

}

@Override

**public int** getViewTypeCount() {

**return TYPE_COUNT**;

}

@Override

**public** View getView(**int** position, View convertView, ViewGroup parent) {

**switch** (getItemViewType(position))

{

**case TYPE_CURRENT**:

convertView = getCurrentView(position, convertView, parent);

**break**;

**case TYPE_HISTORY**:

convertView = getHistoryView(position, convertView, parent);

**break**;

}

**return** convertView;

**private** View getCurrentView(**int** position, View convertView, ViewGroup parent)

{

ViewHolder viewHolder;

**if**(convertView == **null**)

{

convertView = LayoutInflater.*from*(**mContext**).inflate(R.layout.***listitem_current_desens_header***, **null**);

viewHolder = **new** ViewHolder();

viewHolder.**weekNumberTextView** = (TextView) convertView.findViewById(R.id.***tv_week***);

viewHolder.**injectDateTextView** = (TextView) convertView.findViewById(R.id.***tv_inject_date***);

viewHolder.**injectNumberTextView** = (TextView) convertView.findViewById(R.id.***tv_inject_number***);

viewHolder.**healthStatusTextView** = (TextView) convertView.findViewById(R.id.***tv_health_status***);

convertView.setTag(viewHolder);

}

**else**

{

viewHolder = (ViewHolder) convertView.getTag();

}

DesensitizationRecordInfo record = **mRecords**.get(position);

viewHolder.**weekNumberTextView**.setText(String.*valueOf*(record.getWeekNumber()));

viewHolder.**injectDateTextView**.setText(**mDateFormat**.format(record.getInjectDate() * 1000));

viewHolder.**injectNumberTextView**.setText(String.*valueOf*(record.getInjectNumber()));

**if**(record.getHealthStatus().length() == 0)

{

viewHolder.**healthStatusTextView**.setText(**"无"**);

}**else** {

viewHolder.**healthStatusTextView**.setText(record.getHealthStatus());

}

**return** convertView;

}

**private** View getHistoryView(**int** position, View convertView, ViewGroup parent)

{

ViewHolder viewHolder;

**if**(convertView == **null**)

{

convertView = LayoutInflater.*from*(**mContext**).inflate(R.layout.***listitem_desens_header***, **null**);

viewHolder = **new** ViewHolder();

viewHolder.**weekNumberTextView** = (TextView) convertView.findViewById(R.id.***tv_week***);

viewHolder.**injectDateTextView** = (TextView) convertView.findViewById(R.id.***tv_inject_date***);

viewHolder.**injectNumberTextView** = (TextView) convertView.findViewById(R.id.***tv_inject_number***);

viewHolder.**healthStatusTextView** = (TextView) convertView.findViewById(R.id.***tv_health_status***)

convertView.setTag(viewHolder);

}**else**

{viewHolder = (ViewHolder) convertView.getTag(); }

DesensitizationRecordInfo record = **mRecords**.get(position);

viewHolder.**weekNumberTextView**.setText(String.*valueOf*(record.getWeekNumber()));

viewHolder.**injectDateTextView**.setText(**mDateFormat**.format(record.getInjectDate() * 1000));

viewHolder.**injectNumberTextView**.setText(String.*valueOf*(record.getInjectNumber()));

**if**(record.getHealthStatus().length() == 0)

{viewHolder.**healthStatusTextView**.setText(**"无"**); }

**else** {viewHolder.**healthStatusTextView**.setText(record.getHealthStatus()) }

**return** convertView;

}

**private class** ViewHolder

{

**public** TextView **weekNumberTextView**;

**public** TextView **injectDateTextView**;

**public** TextView **injectNumberTextView**;

**public** TextView **healthStatusTextView**;

}

}

**package** com.elinkcare.ubreath.desensitization.adapter;

**import** android.content.Context;

**import** android.graphics.Color;

**import** android.view.LayoutInflater;

**import** android.view.View;

**import** android.view.ViewGroup;

**import** android.widget.BaseAdapter;

**import** android.widget.TextView;

**import** com.elinkcare.ubreath.desensitization.R;

**import** com.elinkcare.ubreath.desensitization.core.data.DesensitizationRecordInfo;

**import** java.text.SimpleDateFormat;

**import** java.util.Calendar;

**import** java.util.List;

*/***

** Created by Jason on 17/2/13.*

**/*

**public class** DesensitizationRecordsAdapter **extends** BaseAdapter{

**private final int TYPE_COUNT** = 2;

**private final int TYPE_CURRENT** = 0;

**private final int TYPE_HISTORY** = 1;

**private long mTodayStart**;

**private long mTodayEnd**;

**private** List<DesensitizationRecordInfo> **mRecords**;

**private** Context **mContext**;

**private** SimpleDateFormat **mTimeFormat** = **new** SimpleDateFormat(**"HH:mm"**);

**private int redColor**;

**private int blueColor**;

**public** DesensitizationRecordsAdapter(Context context, List<DesensitizationRecordInfo> records)

{

**this**.**mContext** = context;

**this**.**mRecords** = records;

**redColor** = **mContext**.getResources().getColor(R.color.***wordred***);

**blueColor** = **mContext**.getResources().getColor(R.color.***wordblue***);

}

@Override

**public void** notifyDataSetChanged() {

Calendar cal = Calendar.*getInstance*();

cal.set(Calendar.***HOUR_OF_DAY***, 0);

cal.set(Calendar.***MINUTE***, 0);

cal.set(Calendar.***SECOND***, 0);

**mTodayStart** = cal.getTimeInMillis() / 1000;

**mTodayEnd** = **mTodayStart** + 24 * 3600;

**super**.notifyDataSetChanged();

}

@Override

**public int** getCount() {

**return mRecords** == **null** ? 0 : **mRecords**.size();

}

@Override

**public** Object getItem(**int** position) {

**return "test"**;

}

@Override

**public long** getItemId(**int** position) {

**return** getItem(position).hashCode();

}

@Override

**public int** getItemViewType(**int** position) {

DesensitizationRecordInfo record = **mRecords**.get(position);

**if**(record.isCurrentRecord())

{

**return TYPE_CURRENT**;

}

**else**

{

**return TYPE_HISTORY**;

}

}

@Override

**public int** getViewTypeCount() {

**return TYPE_COUNT**;

}

@Override

**public** View getView(**int** position, View convertView, ViewGroup parent) {

**switch** (getItemViewType(position))

{

**case TYPE_CURRENT**:

convertView = getCurrentView(position, convertView, parent);

**break**;

**case TYPE_HISTORY**:

convertView = getHistoryView(position, convertView, parent);

**break**;

}

**return** convertView;

}

**private** View getCurrentView(**int** position, View convertView, ViewGroup parent)

{

ViewHolder viewHolder;

**if**(convertView == **null**)

{

convertView = LayoutInflater.*from*(**mContext**).inflate(R.layout.***listitem_current_desens_record***, **null**);

viewHolder = **new** ViewHolder();

viewHolder.**beforePEFTextView** = (TextView) convertView.findViewById(R.id.***tv_before_pef***);

viewHolder.**injectSeperateTextView** = (TextView) convertView.findViewById(R.id.***tv_inject_separate***);

viewHolder.**bottleNumberTextView** = (TextView) convertView.findViewById(R.id.***tv_bottle_number***);

viewHolder.**injectDoseTextView** = (TextView) convertView.findViewById(R.id.***tv_dose***); viewHolder.**injectPartTextView** = (TextView) convertView.findViewById(R.id.***tv_inject_part***);

viewHolder.**injectTimeTextView** = (TextView) convertView.findViewById(R.id.***tv_inject_time***); viewHolder.**afterPEFTextView** = (TextView) convertView.findViewById(R.id.***tv_after_pef***);

viewHolder.**immedWhealSizeTextView** = (TextView) convertView.findViewById(R.id.***tv_immed_wheal_size***);

viewHolder.**immedReactionTextView** = (TextView) convertView.findViewById(R.id.***tv_immed_reaction***);

viewHolder.**delayReactionTextView** = (TextView) convertView.findViewById(R.id.***tv_delay_reaction***);

convertView.setTag(viewHolder);

}

**else**

{

viewHolder = (ViewHolder) convertView.getTag();

}

DesensitizationRecordInfo record = **mRecords**.get(position);

**if**(record.getBeforePEF() == 0)

{

viewHolder.**beforePEFTextView**.setText(**"--"**);

}

**else** {

viewHolder.**beforePEFTextView**.setText(String.*valueOf*(record.getBeforePEF()));

}

viewHolder.**injectSeperateTextView**.setText(record.isInjectSeparate() ? **"是"** : **"否"**);

viewHolder.**bottleNumberTextView**.setText(String.*valueOf*(record.getBottleNumber()));

viewHolder.**injectDoseTextView**.setText(record.getInjectDose());

**if**(record.getBottleNumber() != DesensitizationRecordInfo.*getAutoBottleNumber*(record.getInjectNumber()))

{

viewHolder.**bottleNumberTextView**.setTextColor(**redColor**);

}

**else**

{

viewHolder.**bottleNumberTextView**.setTextColor(**blueColor**);

}

**if**(record.isInjectSeparate())

{

viewHolder.**injectPartTextView**.setText(

record.getInjectPart1() + **"/"** + record.getInjectPart2()

);

viewHolder.**injectTimeTextView**.setText(

**mTimeFormat**.format(record.getInjectTime1() * 1000)

+ **"/"** + **mTimeFormat**.format(record.getInjectTime2() * 1000)

);

viewHolder.**injectSeperateTextView**.setTextColor(**redColor**);

viewHolder.**injectDoseTextView**.setTextColor(**redColor**);

viewHolder.**injectPartTextView**.setTextColor(**redColor**);

viewHolder.**injectTimeTextView**.setTextColor(**redColor**);

}

**else**

{

viewHolder.**injectPartTextView**.setText(record.getInjectPart1());

viewHolder.**injectTimeTextView**.setText(**mTimeFormat**.format(record.getInjectTime1()*1000));

viewHolder.**injectSeperateTextView**.setTextColor(**blueColor**);

viewHolder.**injectDoseTextView**.setTextColor(**blueColor**);

viewHolder.**injectPartTextView**.setTextColor(**blueColor**);

viewHolder.**injectTimeTextView**.setTextColor(**blueColor**);

**if**(record.getInjectDoseTotal() != DesensitizationRecordInfo.*getAutoInjectDose*(record.getInjectNumber()))

{

viewHolder.**injectDoseTextView**.setTextColor(**redColor**);

}

}

**if**(record.getAfterPEF() == 0)

{

viewHolder.**afterPEFTextView**.setText(**"--"**);

}

**else** {

viewHolder.**afterPEFTextView**.setText(String.*valueOf*(record.getAfterPEF()));

}

**if**(record.getImmedWhealSize().length() == 0)

{

viewHolder.**immedWhealSizeTextView**.setText(**"无"**);

}

**else** {

viewHolder.**immedWhealSizeTextView**.setText(record.getImmedWhealSize());

}

**if**(record.getImmedReaction().length() == 0)

{

viewHolder.**immedReactionTextView**.setText(**"无"**);

}

**else**

{

viewHolder.**immedReactionTextView**.setText(record.getImmedReaction());

}

**if**(record.getDelayReaction().length() == 0)

{

viewHolder.**delayReactionTextView**.setText(**"无"**);

}

**else** {

viewHolder.**delayReactionTextView**.setText(record.getDelayReaction());

}

**return** convertView;

}

**private** View getHistoryView(**int** position, View convertView, ViewGroup parent)

{

ViewHolder viewHolder;

**if**(convertView == **null**)

{

convertView = LayoutInflater.*from*(**mContext**).inflate(R.layout.***listitem_desens_record***, **null**);

viewHolder = **new** ViewHolder();

viewHolder.**beforePEFTextView** = (TextView) convertView.findViewById(R.id.***tv_before_pef***);

viewHolder.**injectSeperateTextView** = (TextView) convertView.findViewById(R.id.***tv_inject_separate***);

viewHolder.**bottleNumberTextView** = (TextView) convertView.findViewById(R.id.***tv_bottle_number***);

viewHolder.**injectDoseTextView** = (TextView) convertView.findViewById(R.id.***tv_dose***);

viewHolder.**injectPartTextView** = (TextView) convertView.findViewById(R.id.***tv_inject_part***);

viewHolder.**injectTimeTextView** = (TextView) convertView.findViewById(R.id.***tv_inject_time***);

viewHolder.**afterPEFTextView** = (TextView) convertView.findViewById(R.id.***tv_after_pef***);

viewHolder.**immedWhealSizeTextView** = (TextView) convertView.findViewById(R.id.***tv_immed_wheal_size***);

viewHolder.**immedReactionTextView** = (TextView) convertView.findViewById(R.id.***tv_immed_reaction***);

viewHolder.**delayReactionTextView** = (TextView) convertView.findViewById(R.id.***tv_delay_reaction***);

convertView.setTag(viewHolder);

}

**else**

{

viewHolder = (ViewHolder) convertView.getTag();

}

DesensitizationRecordInfo record = **mRecords**.get(position);

**if**(record.getBeforePEF() == 0)

{

viewHolder.**beforePEFTextView**.setText(**"--"**);

}

**else** {

viewHolder.**beforePEFTextView**.setText(String.*valueOf*(record.getBeforePEF()));

}

viewHolder.**injectSeperateTextView**.setText(record.isInjectSeparate() ? **"是"** : **"否"**);

viewHolder.**bottleNumberTextView**.setText(String.*valueOf*(record.getBottleNumber()));

viewHolder.**injectDoseTextView**.setText(record.getInjectDose());

**if**(record.getBottleNumber() != DesensitizationRecordInfo.*getAutoBottleNumber*(record.getInjectNumber()))

{

viewHolder.**bottleNumberTextView**.setTextColor(**redColor**);

}

**else**

{

viewHolder.**bottleNumberTextView**.setTextColor(Color.***BLACK***);

}

**if**(record.isInjectSeparate())

{

viewHolder.**injectPartTextView**.setText(

record.getInjectPart1() + **"/"** + record.getInjectPart2()

);

viewHolder.**injectTimeTextView**.setText(

**mTimeFormat**.format(record.getInjectTime1() * 1000)

+ **"/"**

+ **mTimeFormat**.format(record.getInjectTime2() * 1000)

);

viewHolder.**injectSeperateTextView**.setTextColor(**redColor**);

viewHolder.**injectDoseTextView**.setTextColor(**redColor**);

viewHolder.**injectPartTextView**.setTextColor(**redColor**);

viewHolder.**injectTimeTextView**.setTextColor(**redColor**);

}

**else**

{

viewHolder.**injectPartTextView**.setText(record.getInjectPart1());

viewHolder.**injectTimeTextView**.setText(**mTimeFormat**.format(record.getInjectTime1()*1000));

viewHolder.**injectSeperateTextView**.setTextColor(Color.***BLACK***);

viewHolder.**injectDoseTextView**.setTextColor(Color.***BLACK***);

viewHolder.**injectPartTextView**.setTextColor(Color.***BLACK***);

viewHolder.**injectTimeTextView**.setTextColor(Color.***BLACK***);

**if**(record.getInjectDoseTotal() != DesensitizationRecordInfo.*getAutoInjectDose*(record.getInjectNumber()))

{

viewHolder.**injectDoseTextView**.setTextColor(**redColor**);

}

}

**if**(record.getAfterPEF() == 0)

{

viewHolder.**afterPEFTextView**.setText(**"--"**);

}

**else** {

viewHolder.**afterPEFTextView**.setText(String.*valueOf*(record.getAfterPEF()));

}

**if**(record.getImmedWhealSize().length() == 0)

{

viewHolder.**immedWhealSizeTextView**.setText(**"无"**);

}

**else** {

viewHolder.**immedWhealSizeTextView**.setText(record.getImmedWhealSize());

}

**if**(record.getImmedReaction().length() == 0)

{

viewHolder.**immedReactionTextView**.setText(**"无"**);

}

**else**

{

viewHolder.**immedReactionTextView**.setText(record.getImmedReaction());

}

**if**(record.getDelayReaction().length() == 0)

{

viewHolder.**delayReactionTextView**.setText(**"无"**);

}

**else** {

viewHolder.**delayReactionTextView**.setText(record.getDelayReaction());

}

**return** convertView;

}

**private class** ViewHolder

{

**public** TextView **beforePEFTextView**;

**public** TextView **injectSeperateTextView**;

**public** TextView **bottleNumberTextView**;

**public** TextView **injectDoseTextView**;

**public** TextView **injectPartTextView**;

**public** TextView **injectTimeTextView**;

**public** TextView **afterPEFTextView**;

**public** TextView **immedWhealSizeTextView**;

**public** TextView **immedReactionTextView**;

**public** TextView **delayReactionTextView**;

}

}

**package** com.elinkcare.ubreath.desensitization.adapter;

**import** com.elinkcare.ubreath.desensitization.core.data.CurveRecordInfo;

**import** com.elinkcare.ubreath.desensitization.widget.CurveChartView;

*/***

** Created by Jason on 17/2/10.*

**/*

**public class** FVCurveAdapter **extends** CurveChartView.ChartAdapter {

**private** CurveRecordInfo **mFVCurve**;

**public** FVCurveAdapter()

{

}

**public void** setFVCurve(CurveRecordInfo fvCurve)

{

**mFVCurve** = fvCurve;

notifyDataSetChanged();

}

@Override

**public int** getCount() {

**if**(**mFVCurve** == **null**) **return** 0;

**if** (**mFVCurve**.getValues() == **null**) **return** 0;

**return mFVCurve**.getValues().**length**;

}

@Override

**public float** getMaxXValue() {

**return** 3.5f;

}

@Override

**public float** getMinXValue() {

**return** 0;

}

@Override

**public float** getMaxYValue() {

**return** 14f;

}

@Override

**public float** getMinYValue() {

**return** 0;

}

@Override

**public float** getXAxisStep() {

**return** 1;

}

@Override

**public float** getYAxisStep() {

**return** 4;

}

@Override

**public float** getYValue(**int** position) {

**return mFVCurve**.getValues()[position] / 100f;

}

@Override

**public float** getXValue(**int** position) {

**if** (position <= 100) {

**return** 0.02f * position;

} **else** {

**return** 2f + 0.04f * (position - 100);

}

}

@Override

**public** String getXTitle() {

**return "L"**;

}

@Override

**public** String getYTitle() {

**return "L/s"**;

}

@Override

**public float** getGridStepX() {

**return** 0.5f;

}

@Override

**public float** getGridStepY() {

**return** 2f;

}

}

**package** com.elinkcare.ubreath.desensitization.controller.data;

*/***

** Created by Jason on 16/9/18.*

**/*

**public class** BTBatteryState {

**public static final int *ST_LESS*** = 0x00;

**public static final int *ST_LOW*** = 0x01;

**public static final int *ST_NORMAL*** = 0x02;

**public static final int *ST_CHARGING*** = 0x03;

**public static final int *ST_CHARGED*** = 0x04;

**private int batteryState**;

**public** BTBatteryState(**int** state)

{

**this**.**batteryState** = **batteryState**;

}

**public int** getBatteryState()

{

**return this**.**batteryState**;

}

}

**package** com.elinkcare.ubreath.desensitization.controller.data;

*/***

** Created by Jason on 16/9/18.*

**/*

**public class** BTCurveRecord {

**public static final int *CURVE_F_T*** = 0x01;

**public static final int *CURVE_F_V*** = 0x02;

**public static final int *CURVE_V_T*** = 0x03;

**private** String **id**;

**private long time**;

**private int type**;

**private int**[] **values**;

**private** String **deviceSN**;

**private** String **userId**;

**public** BTCurveRecord(String id)

{

**this**.**id** = id;

}

**public void** setId(String id)

{

**this**.**id** = id;

}

**public void** setUserId(String userId)

{

**this**.**userId** = userId;

}

**public void** setTime(**long** time)

{

**this**.**time** = time;

}

**public void** setType(**int** type)

{

**this**.**type** = type;

}

**public void** setValues(**int**[] values)

{

**this**.**values** = values;

}

**public void** setDeviceSN(String deviceSN)

{

**this**.**deviceSN** = deviceSN;

}

**public** String getId()

{

**return this**.**id**;

}

**public** String getUserId()

{

**return this**.**userId**;

}

**public long** getTime()

{

**return this**.**time**;

}

**public int** getType()

{

**return this**.**type**;

}

**public int**[] getValues()

{

**return this**.**values**;

}

**public** String getDeviceSN()

{

**return this**.**deviceSN**;

}

}

**package** com.elinkcare.ubreath.desensitization.controller.data;

*/***

** Created by Jason on 16/9/18.*

**/*

**public class** BTInstrumentInfo {

**public final static int *IN_FACTORY_NAME*** = 0x00;

**public final static int *IN_INST_NAME*** = 0x01;

**public final static int *IN_INST_TYPE*** = 0x02;

**public final static int *IN_INST_SN*** = 0x03;

**public final static int *IN_PRODUCT_DATE*** = 0x04;

**public final static int *IN_FIRMWARE_VERSION*** = 0x05;

**public final static int *IN_PCB_VERSION*** = 0x06;

**public final static int *IN_PCBA_VERSION*** = 0x07;

**private int type**;

**public** String **value**;

**public** BTInstrumentInfo(**int** type, String value)

{

**this**.**type** = type;

**this**.**value** = value;

}

**public int** getType()

{

**return this**.**type**;

}

**public** String getValue()

{

**return this**.**value**;

}

}

**package** com.elinkcare.ubreath.desensitization.controller.data;

*/***

** Created by Jason on 16/9/18.*

**/*

**public class** BTInstrumentState {

**public static final int *ST_IDLE*** = 0x00;

**public static final int *ST_TESTING*** = 0x01;

**public static final int *ST_TEST_DONE*** = 0x02;

**public static final int *ST_SHUT_BLE*** = 0x03;

**public static final int *ST_CHARGING*** = 0x04;

**private int instrumentState**;

**public** BTInstrumentState(**int** state)

{

**this**.**instrumentState** = state;

}

**public int** getState()

{

**return this**.**instrumentState**;

}

}

**package** com.elinkcare.ubreath.desensitization.controller.data;

*/***

** Created by Jason on 16/9/18.*

**/*

**public class** BTSpirometerDataRecord {

**private** String **id**;

**private** String **userId**;

**private long time**;

**private int temperature**;

**private int pef**;

**private int fev1**;

**private int fvc**;

**private int fev1per**;

**private int fef50**;

**private int fef75**;

**private int fev_d5**;

**private int mmef**;

**private** String **deviceSN**;

**public** BTSpirometerDataRecord(String id)

{

**this**.**id** = id;

}

**public void** setId(String id)

{

**this**.**id** = id;

}

**public void** setUserId(String userId)

{

**this**.**userId** = userId;

}

**public void** setTime(**long** time)

{

**this**.**time** = time;

}

**public void** setTemperature(**int** temperature)

{

**this**.**temperature** = temperature;

}

**public void** setPEF(**int** pef)

{

**this**.**pef** = pef;

}

**public void** setFEV1(**int** pev1)

{

**this**.**fev1** = pev1;

}

**public void** setFVC(**int** fvc)

{

**this**.**fvc** = fvc;

}

**public void** setFEV1per(**int** fev1per)

{

**this**.**fev1per** = fev1per;

}

**public void** setFEF50(**int** pef50)

{

**this**.**fef50** = pef50;

}

**public void** setFEF75(**int** pef75)

{

**this**.**fef75** = pef75;

}

**public void** setFEVd5(**int** fev_d5)

{

**this**.**fev_d5** = fev_d5;

}

**public void** setMMEF(**int** mmef)

{

**this**.**mmef** = mmef;

}

**public void** setDeviceSN(String deviceSN)

{

**this**.**deviceSN** = deviceSN;

}

**public** String getId()

{

**return this**.**id**;

}

**public** String getUserId()

{

**return this**.**userId**;

}

**public long** getTime()

{

**return this**.**time**;

}

**public int** getTemperature()

{

**return this**.**temperature**;

}

**public int** getPEF()

{

**return this**.**pef**;

}

**public int** getFEV1()

{

**return this**.**fev1**;

}

**public int** getFVC()

{

**return this**.**fvc**;

}

**public int** getFEV1per()

{

**return this**.**fev1per**;

}

**public int** getFEF50()

{

**return this**.**fef50**;

}

**public int** getFEF75()

{

**return this**.**fef75**;

}

**public int** getFEVd5()

{

**return this**.**fev_d5**;

}

**public int** getMMEF()

{

**return this**.**mmef**;

}

**public** String getDeviceSN()

{

**return this**.**deviceSN**;

}

}

**package** com.elinkcare.ubreath.desensitization.controller.data;

*/***

** Created by Jason on 16/9/18.*

**/*

**public class** BTSynchronizeState {

**private long time**;

**public void** setTime(**long** time)

{

**this**.**time** = time;

}

**public long** getTime()

{

**return this**.**time**;

}

}

**package** com.elinkcare.ubreath.desensitization.controller.data;

*/***

** Created by Jason on 16/9/18.*

**/*

**public class** BTUserHealthInfo {

**private** String **name** = **"USERB"**;

**private int sex** = 0; *//0-male, 1-female*

**private long birth** = 611856000;

**private int height** = 175;*//cm*

**private int weight** = 60;*//kg*

**public void** setName(String name)

{

**this**.**name** = name;

}

**public void** setSex(**int** sex)

{

**this**.**sex** = sex;

}

**public void** setBirth(**long** birth)

{

**this**.**birth** = birth;

}

**public void** setHeight(**int** height)

{

**this**.**height** = height;

}

**public void** setWeight(**int** weight)

{

**this**.**weight** = weight;

}

**public** String getName()

{

**return this**.**name**;

}

**public int** getSex()

{

**return this**.**sex**;

}

**public long** getBirth()

{

**return this**.**birth**;

}

**public int** getHeight()

{

**return this**.**height**;

}

**public int** getWeight()

{

**return this**.**weight**;

}

}

**package** com.elinkcare.ubreath.desensitization.controller.data;

**public class** DataFrame {

**private static** DataFrame *mErrorFrame* = **new** DataFrame();

**private int cmd**;

**private byte**[] **params**;

**private int crc**;

**public** DataFrame() {

}

**public** DataFrame(**int** cmd, **byte**[] params) {

**this**.**cmd** = cmd;

**this**.**params** = params;

**int** crc = 0;

**int** paramLen = **this**.**params** == **null** ? 0 : **this**.**params**.**length**;

**int** len = 1 + paramLen + 2 + 1;

crc += 0x55 + 0xAA + len;

crc += cmd;

**for** (**int** i = 0; i < paramLen; i++) {

crc += 0xFF & **this**.**params**[i];

}

**this**.**crc** = crc & 0xFFFF;

}

**public int** getCommand() {

**return this**.**cmd**;

}

**public byte**[] getParams() {

**return this**.**params**;

}

**public static** DataFrame fromBytes(**byte**[] data) {

*mErrorFrame*.**cmd** = -1;

**if** (data.**length** < 2) **return** *mErrorFrame*;

**int** head0 = (**int**) (0xFF & data[0]);

**int** head1 = (**int**) (0xFF & data[1]);

**if** (head0 != 0x55) **return** *mErrorFrame*;

**if** (head1 != 0xAA) **return** *mErrorFrame*;

**int** len = (**int**) (0xFF & data[2]);

**int** paramLen = len - 1 - 2 - 1;

**if** (data.**length** < paramLen + 7) **return null**;

**int** totalLen = data.**length**;

DataFrame frame = **new** DataFrame();

frame.**cmd** = (**int**) (0xFF & data[3]);

**if** (paramLen > 0) {

frame.**params** = **new byte**[paramLen];

**for** (**int** i = 0; i < paramLen; i++) {

frame.**params**[i] = data[i + 4];

}

}

frame.**crc** = (data[totalLen - 3] & 0xFF)

| ((data[totalLen - 2] & 0xFF) << 8);

**if** (frame.**crc** != *calCrc*(data, totalLen - 3)) frame.**cmd** = -1;

**return** frame;

}

**public byte**[] toBytes() {

**int** paramLen = **params** == **null** ? 0 : **params**.**length**;

**int** totalLen = 2 + 1 + 1 + paramLen + 2 + 1;

**byte**[] result = **new byte**[totalLen];

result[0] = 0x55;

result[1] = (**byte**) 0xAA;

result[2] = (**byte**) (1 + paramLen + 2 + 1);

result[3] = (**byte**) **cmd**;

**for** (**int** i = 0; i < paramLen; i++) {

result[i + 4] = **params**[i];

}

result[totalLen - 3] = (**byte**) (**crc** & 0xFF);

result[totalLen - 2] = (**byte**) ((**crc** & 0xFF00) >> 8);

result[totalLen - 1] = 0x0D;

**return** result;

}

**private static int** calCrc(**byte**[] data, **int** len) {

**int** sum = 0;

**for** (**int** i = 0; i < len && i < data.**length**; i++) {

sum += 0xFF & data[i];

}

**return** sum & 0xFFFF;

}

}

**package** com.elinkcare.ubreath.desensitization.controller.data;

*/***

** Created by Jason on 16/9/21.*

**/*

**public class** G2Device {

**private** String **id**;

**private** String **deviceSN**;

**private** String **name**;

**public** G2Device(String id)

{

**this**.**id** = id;

}

**public void** setDeviceSN(String deviceSN)

{

**this**.**deviceSN** = deviceSN;

}

**public void** setName(String name)

{

**this**.**name** = name;

}

**public** String getId()

{

**return this**.**id**;

}

**public** String getDeviceSN()

{

**return this**.**deviceSN**;

}

**public** String getName()

{

**return this**.**name**;

}

}

**package** com.elinkcare.ubreath.desensitization.controller.servlet;

**import** com.elinkcare.ubreath.desensitization.controller.data.BTBatteryState;

**import** com.elinkcare.ubreath.desensitization.controller.data.DataFrame;

*/***

** Created by Jason on 16/9/18.*

**/*

**public class** BatteryStateServlet **extends** GattServlet<BTBatteryState>{

**private** BTBatteryState **mBatteryState**;

**public** BatteryStateServlet(**int** command) {

**super**(command);

}

@Override

**public** BTBatteryState getValue() {

**return mBatteryState**;

}

@Override

**public** DataFrame handlePost(DataFrame frame, String uuid) {

**byte**[] params = frame.getParams();

**if**(params != **null** && params.**length** > 0)

{

**mBatteryState** = **new** BTBatteryState((**int**) (0xFF & params[0]));

}

**return new** DataFrame(**mCommand**, **new byte**[]{0x01});

}

}

**package** com.elinkcare.ubreath.desensitization.controller.servlet;

**import** android.util.Log;

**import** com.elinkcare.ubreath.desensitization.controller.data.BTCurveRecord;

**import** com.elinkcare.ubreath.desensitization.controller.data.DataFrame;

**import** java.util.Calendar;

*/***

** Created by Jason on 16/9/18.*

**/*

**public class** CurveServlet **extends** GattServlet<BTCurveRecord>{

**private boolean mFirstFrame** = **true**;

**private long mRecordTime**;

**private int mType**;

**private** BTCurveRecord **mRecord**;

**private** BTCurveRecord **mTempRecord**;

**public** CurveServlet(**int** command) {

**super**(command);

}

@Override

**public** BTCurveRecord getValue() {

**return mRecord**;

}

@Override

**public** DataFrame handlePost(DataFrame frame, String uuid) {

DataFrame resultFrame = **new** DataFrame(**mCommand**, **new byte**[]{0x01});

**if**(**mFirstFrame**)

{

handleFirstPost(frame, uuid);

**mFirstFrame** = **false**;

}

**else**

{

handleNextPost(frame, uuid);

}

**return** resultFrame;

}

**private void** handleFirstPost(DataFrame frame, String uuid) {

**byte**[] params = frame.getParams();

**if**(params == **null** || params.**length** != 6)**return**;

**int** index = 0;

**int** year = 0xFF & params[index ++];

year = year < 50 ? 2000 + year : 1900 + year;

**int** month = (0xFF & params[index ++]) - 1;

**int** day = 0xFF & params[index ++];

**int** hour = 0xFF & params[index ++];

**int** minute = 0xFF & params[index ++];

**int** second = 0xFF & params[index];

Calendar cal = Calendar.*getInstance*();

cal.set(year, month, day, hour, minute, second);

**mRecordTime** = cal.getTimeInMillis() / 1000;

BTCurveRecord record = **new** BTCurveRecord(**null**);

record.setTime(**mRecordTime**);

**mTempRecord** = record;

}

**private void** handleNextPost(DataFrame frame, String uuid)

{

**byte**[] params = frame.getParams();

**if**(params == **null** || params.**length** == 0)**return**;

**int** index = 0;

**int** type = params[index++];

**if**(type == 0)

{

**mRecord** = **mTempRecord**;

**mTempRecord** = **null**;

Log.*e*(**"CurveServlet"**, **"type = "** + **mRecord**.getType() + **"//////////////////////////////////////////"**);

**return**;

}

**if**(**mTempRecord** == **null**)

{

Log.*e*(**"CurveServlet"**, **"next curve type = "** + type);

**mTempRecord** = **new** BTCurveRecord(**null**);

**mTempRecord**.setTime(**mRecordTime**);

**mRecord** = **null**;

}

**mTempRecord**.setType(type);

**int** valueLength = (params.**length** - 1) /2;

**if**(valueLength <= 0)**return**;

**int**[] values = **new int**[valueLength];

**for**(**int** i = 0; i < valueLength; i++)

{

values[i] = 0xFF & params[index ++];

values[i] = ((0xFF & params[index ++]) << 8 ) | values[i];

}

**if**(**mTempRecord**.getValues() == **null**)

{

**mTempRecord**.setValues(values);

}

**else**

{

**int**[] oldValues = **mTempRecord**.getValues();

**int**[] newValues = **new int**[oldValues.**length** + values.**length**];

**for**(**int** i = 0; i < oldValues.**length**; i++)

{

newValues[i] = oldValues[i];

}

**for**(**int** i = oldValues.**length**; i < newValues.**length**; i++)

{

newValues[i] = values[i - oldValues.**length**];

}

**mTempRecord**.setValues(newValues);

}

}

}

**package** com.elinkcare.ubreath.desensitization.controller.servlet;

**import** com.elinkcare.ubreath.desensitization.controller.data.BTSpirometerDataRecord;

**import** com.elinkcare.ubreath.desensitization.controller.data.DataFrame;

**import** java.util.Calendar;

*/***

** Created by Jason on 16/9/18.*

**/*

**public class** DataRecordServlet **extends** GattServlet<BTSpirometerDataRecord>{

**private** BTSpirometerDataRecord **mDataRecord**;

**public** DataRecordServlet(**int** command) {

**super**(command);

}

@Override

**public** BTSpirometerDataRecord getValue() {

**return mDataRecord**;

}

@Override

**public** DataFrame handlePost(DataFrame frame, String uuid) {

**byte**[] params = frame.getParams();

DataFrame resultFrame = **new** DataFrame(**mCommand**, **new byte**[]{0x01});

**if**(params == **null** || params.**length** <= 0)**return null**;

*//time*

**int** index = 0;

**int** year = (**int**)(0xFF & params[index ++]);

year = year < 50 ? 2000 + year : 1900 + year;

**int** month = (**int**)(0xFF & params[index ++]) - 1;

**int** day = (**int**) (0xFF & params[index ++]);

**int** hour = (**int**) (0xFF & params[index ++]);

**int** minute = (**int**) (0xFF & params[index ++]);

**int** second = (**int**) (0xFF & params[index ++]);

Calendar calendar = Calendar.*getInstance*();

calendar.set(year, month, day, hour, minute, second);

BTSpirometerDataRecord record = **new** BTSpirometerDataRecord(**null**);

record.setTime(calendar.getTimeInMillis() / 1000);

*//BTPS*

**int** btps = (0xFF & params[index ++]);

btps = ((0xFF & params[index ++]) << 8) | btps;

record.setTemperature(btps);

*//PEF*

**int** pef = (0xFF & params[index ++]);

pef = ((0xFF & params[index ++]) << 8) | pef;

record.setPEF(pef);

*//PEV1*

**int** pev1 = (0xFF & params[index ++]);

pev1 = ((0xFF & params[index ++]) << 8) | pev1;

record.setFEV1(pev1);

*//FVC*

**int** fvc = (0xFF & params[index ++]);

fvc = ((0xFF & params[index ++]) << 8) | fvc;

record.setFVC(fvc);

*//FEV 1%*

**int** fev1 = (0xFF & params[index ++]);

fev1 = ((0xFF & params[index ++]) << 8) | fev1;

record.setFEV1per(fev1);

*//PEF50*

**int** pef50 = (0xFF & params[index ++]);

pef50 = ((0xFF & params[index ++]) << 8) | pef50;

record.setFEF50(pef50);

*//PEF75*

**int** pef75 = (0xFF & params[index ++]);

pef75 = ((0xFF & params[index ++]) << 8) | pef75;

record.setFEF75(pef75);

*//FEV0.5*

**int** fev_d5 = (0xFF & params[index ++]);

fev_d5 = ((0xFF & params[index ++]) << 8) | fev_d5;

record.setFEVd5(fev_d5);

*//MMEF*

**int** mmef = (0xFF & params[index ++]);

mmef = ((0xFF & params[index ++]) << 8) | mmef;

record.setMMEF(mmef);

**mDataRecord** = record;

**return** resultFrame;

}

}

**package** com.elinkcare.ubreath.desensitization.controller.servlet;

**import** com.elinkcare.ubreath.desensitization.controller.data.DataFrame;

*/***

** Created by Jason on 16/9/18.*

**/*

**public abstract class** GattServlet<T> {

**protected int mCommand**;

**public** GattServlet(**int** command)

{

**this**.**mCommand** = command;

}

**public abstract** T getValue();

**public abstract** DataFrame handlePost(DataFrame frame, String uuid);

}

**package** com.elinkcare.ubreath.desensitization.controller.servlet;

**import** com.elinkcare.ubreath.desensitization.controller.data.BTInstrumentInfo;

**import** com.elinkcare.ubreath.desensitization.controller.data.DataFrame;

**import** java.util.Arrays;

*/***

** Created by Jason on 16/9/18.*

**/*

**public class** InstrumentInfoServlet **extends** GattServlet<BTInstrumentInfo> {

**private** BTInstrumentInfo **mInstrumentInfo**;

**public** InstrumentInfoServlet(**int** command)

{

**super**(command);

}

@Override

**public** BTInstrumentInfo getValue() {

**return mInstrumentInfo**;

}

@Override

**public** DataFrame handlePost(DataFrame frame, String uuid) {

**byte**[] params = frame.getParams();

**if**(params != **null** && params.**length** > 1) {

**int** type = (**int**) (0xFF & params[0]);

String value = **new** String(Arrays.*copyOfRange*(params, 1, params.**length** - 1));

**mInstrumentInfo** = **new** BTInstrumentInfo(type, value);

}

**return new** DataFrame(**mCommand**, **new byte**[]{0x01});

}

}

**package** com.elinkcare.ubreath.desensitization.controller.servlet;

**import** com.elinkcare.ubreath.desensitization.controller.data.BTInstrumentState;

**import** com.elinkcare.ubreath.desensitization.controller.data.DataFrame;

*/***

** Created by Jason on 16/9/18.*

**/*

**public class** InstrumentStateServlet **extends** GattServlet<BTInstrumentState> {

**private** BTInstrumentState **mInstrumentState**;

**public** InstrumentStateServlet(**int** command) {

**super**(command);

}

@Override

**public** BTInstrumentState getValue() {

**return mInstrumentState**;

}

@Override

**public** DataFrame handlePost(DataFrame frame, String uuid) {

**byte**[] params = frame.getParams();

**if**(params != **null** && params.**length** > 0) {

**mInstrumentState** = **new** BTInstrumentState((**int**)(0xFF & params[0]));

}

**return new** DataFrame(**mCommand**, **new byte**[]{0x01});

}

}

**package** com.elinkcare.ubreath.desensitization.controller.servlet;

**import** com.elinkcare.ubreath.desensitization.controller.data.DataFrame;

*/***

** Created by yejiasheng on 16/9/27.*

**/*

**public class** OtherCmdServlet **extends** GattServlet<String>{

**public** OtherCmdServlet(**int** command) {

**super**(command);

}

@Override

**public** String getValue() {

**return null**;

}

@Override

**public** DataFrame handlePost(DataFrame frame, String uuid) {

DataFrame resultFrame = **new** DataFrame(**mCommand**, **new byte**[]{0x01});

**return** resultFrame;

}

}

**package** com.elinkcare.ubreath.desensitization.controller.servlet;

**import** com.elinkcare.ubreath.desensitization.controller.data.DataFrame;

**import** java.util.Calendar;

*/***

** Created by Jason on 16/9/18.*

**/*

**public class** SetSystemTimeServlet **extends** GattServlet{

**public** SetSystemTimeServlet(**int** command) {

**super**(command);

}

@Override

**public** Object getValue() {

**return null**;

}

@Override

**public** DataFrame handlePost(DataFrame frame, String uuid) {

Calendar cal = Calendar.*getInstance*();

**int** year = cal.get(Calendar.***YEAR***) % 100;

**int** month = cal.get(Calendar.***MONTH***) + 1;

**int** day = cal.get(Calendar.***DAY_OF_MONTH***);

**int** hour = cal.get(Calendar.***HOUR_OF_DAY***);

**int** minute = cal.get(Calendar.***MINUTE***);

**int** second = cal.get(Calendar.***SECOND***);

**byte**[] time = **new byte**[6];

time[0] = (**byte**) year;

time[1] = (**byte**) month;

time[2] = (**byte**) day;

time[3] = (**byte**) hour;

time[4] = (**byte**) minute;

time[5] = (**byte**) second;

DataFrame resultFrame = **new** DataFrame(**mCommand**, time);

**return** resultFrame;

}

}

**package** com.elinkcare.ubreath.desensitization.controller.servlet;

**import** com.elinkcare.ubreath.desensitization.controller.data.BTUserHealthInfo;

**import** com.elinkcare.ubreath.desensitization.controller.data.DataFrame;

**import** com.elinkcare.ubreath.desensitization.utils.CommonUtils;

*/***

** Created by yejiasheng on 16/9/18.*

**/*

**public class** SetUserInfoServlet **extends** GattServlet<BTUserHealthInfo>{

**private** BTUserHealthInfo **mHealthInfo**;

**public** SetUserInfoServlet(**int** command, BTUserHealthInfo healthInfo) {

**super**(command);

**this**.**mHealthInfo** = healthInfo;

}

@Override

**public** BTUserHealthInfo getValue() {

**return null**;

}

@Override

**public** DataFrame handlePost(DataFrame frame, String uuid) {

**byte**[] name = **"USERA"**.getBytes();

**byte**[] sex;

**switch** (**mHealthInfo**.getSex())

{

**case** 1:

sex = **"female"**.getBytes();

**break**;

**default**:

sex = **"male"**.getBytes();

}

**int** paramLen = name.**length** + 1

+ sex.**length** + 1

+ 3;*//age height weight*

**byte**[] params = **new byte**[paramLen];

**for**(**int** i = 0; i < name.**length**; i++)

{

params[i] = name[i];

}

**for**(**int** i = 0; i < sex.**length**; i++)

{

params[name.**length** + i + 1] = sex[i];

}

params[paramLen - 3] = (**byte**) CommonUtils.*getAge*(**mHealthInfo**.getBirth() * 1000);

params[paramLen - 2] = (**byte**) **mHealthInfo**.getHeight();

params[paramLen - 1] = (**byte**) **mHealthInfo**.getWeight();

DataFrame resultFrame = **new** DataFrame(**mCommand**, params);

**return** resultFrame;

}

}

**package** com.elinkcare.ubreath.desensitization.controller.servlet;

**import** com.elinkcare.ubreath.desensitization.controller.data.DataFrame;

**import** java.util.Calendar;

*/***

** Created by Jason on 16/9/18.*

**/*

**public class** SynchronizeStateServlet **extends** GattServlet{

**private long mLastRecordTime**;

**public** SynchronizeStateServlet(**int** command, **long** lastRecordTime) {

**super**(command);

**this**.**mLastRecordTime** = lastRecordTime;

}

@Override

**public** Object getValue() {

**return null**;

}

@Override

**public** DataFrame handlePost(DataFrame frame, String uuid) {

**long** currentTime = -1;

**byte**[] params = frame.getParams();

**if**(params != **null** && params.**length** > 0)

{

**int** year = 0xFF & params[0];

year = year < 50 ? 2000 + year : 1900 + year;

**int** month = (0xFF & params[1]) - 1;

**int** day = 0xFF & params[2];

**int** hour = 0xFF & params[3];

**int** minute = 0xFF & params[4];

**int** second = 0xFF & params[5];

Calendar cal = Calendar.*getInstance*();

cal.set(year, month, day, hour, minute, second);

currentTime = cal.getTimeInMillis() / 1000;

}

**byte** uploadState = 1; *//0- 未上传, 1-已上传*

*//if(mLastRecordTime >= currentTime)*

*//{*

*// uploadState = 1;*

*//}*

DataFrame resultFrame = **new** DataFrame(**mCommand**, **new byte**[]{uploadState});

**return** resultFrame;

}

}

**package** com.elinkcare.ubreath.desensitization.controller;

**import** android.bluetooth.BluetoothDevice;

**import** android.bluetooth.BluetoothGatt;

**import** android.bluetooth.BluetoothGattCallback;

**import** android.bluetooth.BluetoothGattCharacteristic;

**import** android.bluetooth.BluetoothGattService;

**import** android.bluetooth.BluetoothProfile;

**import** android.content.Context;

**import** android.os.Handler;

**import** android.util.Log;

**import** com.elinkcare.ubreath.desensitization.controller.data.DataFrame;

**import** java.util.ArrayList;

**import** java.util.Arrays;

**import** java.util.LinkedList;

**import** java.util.List;

**import** java.util.UUID;

**import** java.util.concurrent.locks.ReentrantReadWriteLock;

*/***

** Created by Jason on 16/9/18.*

**/*

**public class** BtDeviceController {

**private static final** String ***TAG*** = **"BtDeviceController"**;

**private** BluetoothDevice **mDevice**;

**private** Context **mContext**;

**private static final int *MAX_FRAME_PARAM_LEN*** = 13;

**private boolean mConnected** = **false**;

**private** BluetoothGatt **mBluetoothGatt**;

**private** BluetoothGattService **mGattService**;

**private** BluetoothGattCharacteristic **mReadCharacteristic**;

**private** BluetoothGattCharacteristic **mWriteCharacteristic**;

**private** BluetoothGattCallback **mGattCallback** = **new** SpirometerGattCallback();

**private byte**[] **mReceivedDataBuffer**;

**private static final** UUID ***SERVICE_UUID*** = UUID

.*fromString*(**"0000FFF0-0000-1000-8000-00805F9B34FB"**);

**private static final** UUID ***CHARACTERISTIC_WRITE_UUID*** = UUID

.*fromString*(**"0000FFF6-0000-1000-8000-00805F9B34FB"**);

**private static final** UUID ***CHARACTERISTIC_READ_UUID*** = UUID

.*fromString*(**"0000FFF7-0000-1000-8000-00805F9B34FB"**);

**private** Thread **mSendThread** = **null**;

**private** LinkedList<DataFrame> **mSendDataFrameList** = **new** LinkedList<>();

**private** ReentrantReadWriteLock **mSendLock** = **new** ReentrantReadWriteLock();

**private** OnBtDeviceReceivedListener **mListener** = **null**;

**private** Handler **mHandler**;

**public** BtDeviceController(BluetoothDevice device, Context context) {

**this**.**mDevice** = device;

**this**.**mContext** = context;

**mHandler** = **new** Handler(context.getMainLooper());

}

**public void** connect() {

**mBluetoothGatt** = **mDevice**.connectGatt(**mContext**, **false**, **mGattCallback**);

}

**public void** disconnect() {

*//Log.e(TAG, "try disconnect " + mConnected);*

**if** (!**mConnected**) {

handleConnectStateChanged(**false**);

**return**;

}

**mBluetoothGatt**.disconnect();

*//mBluetoothGatt.close();*

*//handleConnectStateChanged(false);*

}

**public boolean** isConnected() {

**return this**.**mConnected**;

}

**public void** setConnected(**boolean** connected)

{

**this**.**mConnected** = connected;

}

**public void** writeSendDataFrame(DataFrame frame) {

**if** (frame == **null**) **return**;

**try** {

**mSendLock**.writeLock().lock();

**mSendDataFrameList**.addLast(frame);

initSendThread();

} **finally** {

**mSendLock**.writeLock().unlock();

}

}

**private** List<DataFrame> splitDataFrame(DataFrame frame) {

**byte**[] params = frame.getParams();

**int** startIndex = 0;

**int** paramsLen = params.**length**;

List<DataFrame> frames = **new** ArrayList<>();

**while** (paramsLen > ***MAX_FRAME_PARAM_LEN***) {

DataFrame subFrame = **new** DataFrame(frame.getCommand(), Arrays.*copyOfRange*(params, startIndex, startIndex + ***MAX_FRAME_PARAM_LEN***));

frames.add(subFrame);

paramsLen -= ***MAX_FRAME_PARAM_LEN***;

startIndex += ***MAX_FRAME_PARAM_LEN***;

}

**if** (paramsLen > 0) {

DataFrame subFrame = **new** DataFrame(frame.getCommand(), Arrays.*copyOfRange*(params, startIndex, startIndex + paramsLen));

frames.add(subFrame);

}

**return** frames;

}

**public void** setOnBtDeviceReceivedListener(OnBtDeviceReceivedListener listener) {

**this**.**mListener** = listener;

}

**private synchronized void** handleDataReceived(**byte**[] data, UUID uuid) {

StringBuilder builder = **new** StringBuilder(**"receive data: "**);

**for** (**int** i = 0; i < data.**length**; i++) {

builder.append(String.*format*(**"%02x "**, data[i]));

}

*//Log.e(TAG, builder.toString());*

DataFrame frame;

**if** (**mReceivedDataBuffer** == **null**) {

frame = DataFrame.*fromBytes*(data);

**if** (frame == **null**) {

appendToDataBuffer(data);

**return**;

}

} **else** {

appendToDataBuffer(data);

frame = DataFrame.*fromBytes*(**mReceivedDataBuffer**);

**if** (frame == **null**) **return**;

}

*//Log.e(TAG, "frame = " + frame);*

**mReceivedDataBuffer** = **null**;

**if** (frame.getCommand() < 0) {

**return**;

}

**if** (**mListener** != **null**) **mListener**.onDataReceived(frame, uuid.toString());

}

**private void** appendToDataBuffer(**byte**[] data) {

**if** (**mReceivedDataBuffer** == **null**) {

*//if(data.length < 2)return;*

**mReceivedDataBuffer** = data;

} **else** {

**byte**[] temp = **mReceivedDataBuffer**;

**if** (**mReceivedDataBuffer**.**length** >= 2) {

**int** head0 = 0xFF & **mReceivedDataBuffer**[0];

**int** head1 = 0xFF & **mReceivedDataBuffer**[1];

**if** (head0 != 0x55 || head1 != 0xAA) {

**mReceivedDataBuffer** = data;

**return**;

}

}

**mReceivedDataBuffer** = **new byte**[temp.**length** + data.**length**];

**for** (**int** i = 0; i < temp.**length**; i++) {

**mReceivedDataBuffer**[i] = temp[i];

}

**for** (**int** i = 0; i < data.**length**; i++) {

**mReceivedDataBuffer**[temp.**length** + i] = data[i];

}

}

}

**private synchronized void** sendData(**byte**[] data) {

**if** (!**mConnected**) **return**;

StringBuilder builder = **new** StringBuilder(**"send data : "**);

**for** (**int** i = 0; i < data.**length**; i++) {

builder.append(String.*format*(**"%02x "**, data[i]));

}

*//Log.e(TAG, builder.toString());*

**if** (**mWriteCharacteristic** == **null**) {

**mGattService** = **mBluetoothGatt**.getService(***SERVICE_UUID***);

**if** (**mGattService** == **null**) **return**;

**mWriteCharacteristic** = **mGattService**.getCharacteristic(***CHARACTERISTIC_WRITE_UUID***);

}

**if** (**mWriteCharacteristic** == **null**) **return**;

**mWriteCharacteristic**.setValue(data);

**mBluetoothGatt**.writeCharacteristic(**mWriteCharacteristic**);

}

**private synchronized void** handleConnectStateChanged(**boolean** connect) {

**this**.**mConnected** = connect;

**if** (**mListener** != **null**) **mListener**.onConnectionChanged(connect);

}

**private class** SpirometerGattCallback **extends** BluetoothGattCallback {

@Override

**public void** onConnectionStateChange(BluetoothGatt gatt, **int** status, **int** newState) {

**super**.onConnectionStateChange(gatt, status, newState);

**if** (newState == BluetoothProfile.***STATE_CONNECTED***) {

Log.*e*(***TAG***, **"Connected to GATT server."**);

*// Attempts to discover services after successful connection.*

**mBluetoothGatt**.discoverServices();

handleConnectStateChanged(**true**);

} **else if** (newState == BluetoothProfile.***STATE_CONNECTING***) {

} **else if** (newState == BluetoothProfile.***STATE_DISCONNECTED***) {

Log.*e*(***TAG***, **"bt device disconnected"**);

**mBluetoothGatt**.close();

handleConnectStateChanged(**false**);

} **else if** (newState == BluetoothProfile.***STATE_DISCONNECTING***) {

*//Log.e(TAG, "bt device disconnecting");*

}

}

@Override

**public void** onServicesDiscovered(BluetoothGatt gatt, **int** status) {

**super**.onServicesDiscovered(gatt, status);

**if** (status == BluetoothGatt.***GATT_SUCCESS***) {

**if** (**mBluetoothGatt** != **null**) {

**mGattService** = **mBluetoothGatt**.getService(***SERVICE_UUID***);

**if** (**mGattService** == **null**) **return**;

**mReadCharacteristic** = **mGattService**.getCharacteristic(***CHARACTERISTIC_READ_UUID***);

**if** (**mReadCharacteristic** != **null**) {

**mBluetoothGatt**.setCharacteristicNotification(**mReadCharacteristic**, **true**);

}

}

} **else** {

Log.*w*(***TAG***, **"onServicesDiscovered received: "** + status);

}

}

@Override

**public void** onCharacteristicChanged(BluetoothGatt gatt, BluetoothGattCharacteristic characteristic) {

**super**.onCharacteristicChanged(gatt, characteristic);

handleDataReceived(characteristic.getValue(), characteristic.getUuid());

}

@Override

**public void** onCharacteristicRead(BluetoothGatt gatt, BluetoothGattCharacteristic characteristic, **int** status) {

**super**.onCharacteristicRead(gatt, characteristic, status);

handleDataReceived(characteristic.getValue(), characteristic.getUuid());

}

}

**private synchronized void** initSendThread() {

**if** (**mSendThread** != **null**) **return**;

**mSendThread** = **new** Thread(**new** Runnable() {

@Override

**public void** run() {

**while** (**mSendDataFrameList**.size() > 0) {

**try** {

**mSendLock**.writeLock().lock();

DataFrame frame = **mSendDataFrameList**.pollFirst();

**if** (frame == **null**) **break**;

**byte**[] data = frame.toBytes();

**int** remain = data.**length**;

**int** startIndex = 0;

**int** n = 0;

**while** (remain > 20) {

*//Log.e(TAG, "send in loop " + n + ":" + startIndex + ", 20");*

sendData(Arrays.*copyOfRange*(data, startIndex, startIndex + 20));

remain -= 20;

startIndex += 20;

Thread.*sleep*(150);

n++;

}

**if** (remain > 0) {

*//Log.e(TAG, "send remain " + startIndex + ", " + remain);*

sendData(Arrays.*copyOfRange*(data, startIndex, startIndex + remain));

}

*// Thread.sleep(150);*

} **catch** (InterruptedException e) {

e.printStackTrace();

} **finally** {

**mSendLock**.writeLock().unlock();

}

}

**mSendThread** = **null**;

}

});

**mSendThread**.start();

}

**public static interface** OnBtDeviceReceivedListener {

**public void** onConnectionChanged(**boolean** connected);

**public void** onDataReceived(DataFrame frame, String uuid);

}

}

**package** com.elinkcare.ubreath.desensitization.controller;

**import** android.bluetooth.BluetoothDevice;

**import** java.util.ArrayList;

**import** java.util.List;

*/***

** Created by Jason on 16/11/22.*

**/*

**public class** BtDeviceManager {

**private static final** String ***TAG*** = **"BtDeviceManager"**;

**private static** BtDeviceManager *mManager*;

**private** List<BluetoothDevice> **mDevices** = **new** ArrayList<>();

**private** BtDeviceManager()

{

}

**public static** BtDeviceManager getInstance()

{

**if**(*mManager* != **null**)**return** *mManager*;

**synchronized** (***TAG***)

{

**if**(*mManager* != **null**)**return** *mManager*;

*mManager* = **new** BtDeviceManager();

**return** *mManager*;

}

}

**public synchronized void** refreshBtDevices(List<BluetoothDevice> devices)

{

**if**(devices != **null**) {

**mDevices**.clear();

**mDevices**.addAll(devices);

}

}

**public synchronized** List<BluetoothDevice> getDevices()

{

List<BluetoothDevice> devices = **new** ArrayList<>();

devices.addAll(**mDevices**);

**return** devices;

}

**public synchronized** BluetoothDevice getDevice(String address)

{

**if**(address == **null**)**return null**;

**for**(**int** i = 0; i < **mDevices**.size(); i++)

{

**if**(address.equals(**mDevices**.get(i).getAddress()))

{

**return mDevices**.get(i);

}

}

**return null**;

}

}

**package** com.elinkcare.ubreath.desensitization.controller;

**import** android.bluetooth.BluetoothAdapter;

**import** android.bluetooth.BluetoothDevice;

**import** android.bluetooth.BluetoothManager;

**import** android.content.Context;

**import** android.util.Log;

**import** java.util.ArrayList;

**import** java.util.HashSet;

**import** java.util.Iterator;

**import** java.util.List;

**import** java.util.Set;

**import** java.util.Timer;

**import** java.util.TimerTask;

*/***

** Created by Administrator on 2016/3/22.*

**/*

**public class** BtDeviceScanner {

**private static** BtDeviceScanner *manager* = **new** BtDeviceScanner();

**private** BluetoothAdapter **mBtAdapter**;

**private** Set<BluetoothDevice> **mDeviceSet** = **new** HashSet<BluetoothDevice>();

**private** Timer **mTimer**;

**private long mScanPeriod** = 2000;

**private boolean mIsConnected** = **false**;

**private** Set<IScanChangedWatcher> **mScanChangedWatcherSet** = **new** HashSet<IScanChangedWatcher>();

**private** IScanResultFilter **mScanResultFilter**;

**private** BtDeviceScanner()

{

*//****TODO: nothing***

}

**public static synchronized** BtDeviceScanner getInstance(Context context)

{

**if**(context == **null**) **throw new** NullPointerException(**"BluetoothManager.getInstance(context), context couldn't be null"**);

**if**(*manager*.**mBtAdapter** == **null**)

{

*manager*.**mBtAdapter** = ((BluetoothManager) context.getSystemService(Context.***BLUETOOTH_SERVICE***)).getAdapter();

}

**return** *manager*;

}

**public static interface** IScanChangedWatcher

{

**public void** onChanged(List<BluetoothDevice> devices);

}

**public static interface** IScanResultFilter

{

**public boolean** filter(BluetoothDevice device);

}

**public static class** WHQFilter **implements** IScanResultFilter

{

@Override

**public boolean** filter(BluetoothDevice device) {

**return** device.getName().startsWith(**"eLinkCareWHQ"**);

}

}

**public synchronized void** enableBluetooth()

{

**mBtAdapter**.enable();

}

**public synchronized void** disableBluetooth()

{

**mBtAdapter**.disable();

}

**public synchronized void** startScanDevice()

{

**if**(!**mBtAdapter**.isEnabled())

{

enableBluetooth();

}

**mDeviceSet**.clear();

**mBtAdapter**.startLeScan(**mLeScanCallback**);

**if**(**mTimer** != **null**)

{

**mTimer**.purge();

}

**mTimer** = **new** Timer();

**mTimer**.schedule(**new** TimerTask()

{

@Override

**public void** run() {

**mBtAdapter**.stopLeScan(**mLeScanCallback**);

**mTimer** = **null**;

refreshDevice(**null**);

}

}, **mScanPeriod**);

}

**public synchronized void** stopScanDevice()

{

**if**(**mTimer** != **null**) **mTimer**.purge();

**mBtAdapter**.stopLeScan(**mLeScanCallback**);

**mTimer** = **null**;

}

**public** BluetoothDevice getDevice(String address)

{

**return mBtAdapter**.getRemoteDevice(address);

}

**public void** addScanChangedWatcher(IScanChangedWatcher watcher)

{

**mScanChangedWatcherSet**.add(watcher);

}

**public void** removeScanChangedWatcher(IScanChangedWatcher watcher)

{

**mScanChangedWatcherSet**.remove(watcher);

}

**public void** setScanResultFilter(IScanResultFilter filter)

{

**mScanResultFilter** = filter;

}

**private** BluetoothAdapter.LeScanCallback **mLeScanCallback** = **new** BluetoothAdapter.LeScanCallback()

{

@Override

**public synchronized void** onLeScan(BluetoothDevice device, **int** rssi, **byte**[] scanRecord) {

**if**(**mScanResultFilter** == **null**

|| **mScanResultFilter**.filter(device))

{

**mDeviceSet**.add(device);

}

Log.*e*(**"search device"**, **"device = "** + device);

ArrayList<BluetoothDevice> deviceList = **new** ArrayList<BluetoothDevice>();

deviceList.addAll(**mDeviceSet**);

refreshDevice(deviceList);

}

};

**private synchronized void** refreshDevice(ArrayList<BluetoothDevice> deviceList)

{

Iterator<IScanChangedWatcher> iterator = **mScanChangedWatcherSet**.iterator();

**while**(iterator.hasNext())

{

iterator.next().onChanged(deviceList);

}

}

}

**package** com.elinkcare.ubreath.desensitization.controller;

**import** android.bluetooth.BluetoothDevice;

**import** android.content.ComponentName;

**import** android.content.Context;

**import** android.content.Intent;

**import** android.content.ServiceConnection;

**import** android.os.IBinder;

**import** android.util.Log;

**import** com.elinkcare.ubreath.desensitization.BluetoothLeService;

**import** com.elinkcare.ubreath.desensitization.controller.data.BTBatteryState;

**import** com.elinkcare.ubreath.desensitization.controller.data.BTCurveRecord;

**import** com.elinkcare.ubreath.desensitization.controller.data.BTInstrumentInfo;

**import** com.elinkcare.ubreath.desensitization.controller.data.BTInstrumentState;

**import** com.elinkcare.ubreath.desensitization.controller.data.BTSpirometerDataRecord;

**import** com.elinkcare.ubreath.desensitization.controller.data.BTUserHealthInfo;

**import** com.elinkcare.ubreath.desensitization.controller.data.DataFrame;

**import** com.elinkcare.ubreath.desensitization.controller.servlet.BatteryStateServlet;

**import** com.elinkcare.ubreath.desensitization.controller.servlet.CurveServlet;

**import** com.elinkcare.ubreath.desensitization.controller.servlet.DataRecordServlet;

**import** com.elinkcare.ubreath.desensitization.controller.servlet.InstrumentInfoServlet;

**import** com.elinkcare.ubreath.desensitization.controller.servlet.InstrumentStateServlet;

**import** com.elinkcare.ubreath.desensitization.controller.servlet.OtherCmdServlet;

**import** com.elinkcare.ubreath.desensitization.controller.servlet.SetSystemTimeServlet;

**import** com.elinkcare.ubreath.desensitization.controller.servlet.SetUserInfoServlet;

**import** com.elinkcare.ubreath.desensitization.controller.servlet.SynchronizeStateServlet;

**import** com.elinkcare.ubreath.desensitization.core.ClientManager;

**import** com.elinkcare.ubreath.desensitization.core.data.PatientInfo;

**import** java.util.ArrayList;

**import** java.util.Collections;

**import** java.util.List;

**import** java.util.Timer;

**import** java.util.TimerTask;

*/***

** Created by Jason on 16/9/18.*

**/*

**public class** PfmmedController {

**private final static** String ***TAG*** = **"PfmmedController"**;

**private** Context **mContext**;

**private static** PfmmedController *mController*;

**private static final int *CMD_INST_INFO*** = 0x01;

**private static final int *CMD_INST_STATE*** = 0x02;

**private static final int *CMD_CELL_STATE*** = 0x03;

**private static final int *CMD_GET_DATA*** = 0x04;

**private static final int *CMD_GET_CURVE*** = 0x05;

**private static final int *CMD_CHECK_SYNC*** = 0x06;

**private static final int *CMD_ERROR*** = 0x07;

**private static final int *CMD_GET_USERINFO*** = 0x08;

**private static final int *CMD_SET_USERINFO*** = 0x09;

**private static final int *CMD_SET_SYSTEM_TIME*** = 0x12;

**public static final int *STATE_IDLE*** = 0x00;

**public static final int *STATE_TESTING*** = 0x01;

**public static final int *STATE_TEST_DONE*** = 0x02;

**public static final int *STATE_SHUT_BLE*** = 0x03;

**public static final int *STATE_CHARGING*** = 0x04;

**public static final int *INST_STATE_NO_DEV*** = 0x00;

**public static final int *INST_STATE_CONNECTING*** = 0x01;

**public static final int *INST_STATE_INITIALIZING*** = 0x02;

**public static final int *INST_STATE_IDLE*** = 0x03;

**public static final int *INST_STATE_TESTING*** = 0x04;

**public static final int *INST_STATE_FINISH_TEST*** = 0x05;

**public static final int *INST_STATE_DISCONNECTED*** = 0x06;

**private** BTUserHealthInfo **mCurrentHealthInfo** = **new** BTUserHealthInfo();

**private** BTInstrumentInfo **mInstrumentSN**;

**private** BTInstrumentState **mInstrumentState**;

**private** BTBatteryState **mBatteryState**;

**private static** List<OnConnectionChangedListener> *mOnConnectionChangedListeners* =

Collections.*synchronizedList*(**new** ArrayList<OnConnectionChangedListener>());

**private** OnStateChangedListener **mOnStateChangedListener**;

**private** OnBatteryStateChangedListener **mOnBatteryStateChangedListener**;

**private** OnDataReceivedListener **mOnDataReceivedListener**;

**private** BluetoothDevice **mBtDevice**;

**private** BluetoothLeService **mService**;

**private boolean isBound** = **false**;

**private boolean isConnecting** = **false**;

**private** Timer **mTimeoutTimer**;

**private** ServiceConnection **mServiceConnection** = **new** ServiceConnection() {

@Override

**public void** onServiceConnected(ComponentName name, IBinder service) {

**synchronized** (***TAG***) {

**isBound** = **true**;

**mService** = ((BluetoothLeService.BluetoothLeBinder) service).getBluetoothLeService();

**mService**.connect(**mBtDevice**);

BtDeviceController controller = **mService**.getController();

controller.setOnBtDeviceReceivedListener(**new** OnSpirometerDataReceivedListener());

}

}

@Override

**public void** onServiceDisconnected(ComponentName name) {

}

};

**public synchronized static void** init(Context context) {

**if** (*mController* != **null**) **return**;

*mController* = **new** PfmmedController(context);

}

**public static** PfmmedController getInstance() {

**return** *mController*;

}

**private** PfmmedController(Context context) {

**mContext** = context;

}

**public synchronized void** connect(BluetoothDevice device) {

**isConnecting** = **true**;

**mInstrumentSN** = **null**;

**mInstrumentState** = **null**;

startTimeoutTimer();

**mBtDevice** = device;

**mBufferedCurveSevlet** = **null**;

Intent intent = **new** Intent(**mContext**, BluetoothLeService.**class**);

**mContext**.bindService(intent, **mServiceConnection**, Context.***BIND_AUTO_CREATE***);

}

**public synchronized void** disconnect() {

*//isConnecting = false;*

*//mBtDeviceController.disconnect();*

**mBufferedCurveSevlet** = **null**;

**if** (**mService** == **null**) {

**return**;

}

**try** {

**mService**.disconnect();

} **catch** (Exception e) {

e.printStackTrace();

}

}

**public boolean** isConnected() {

**if** (**mService** == **null**) **return false**;

BtDeviceController controller = **mService**.getController();

**if** (controller == **null**) **return false**;

**boolean** connection = controller.isConnected();

**synchronized** (***TAG***) {

**if** (**isBound** && !connection) {

**mContext**.unbindService(**mServiceConnection**);

**isBound** = **false**;

}

}

**return** connection;

}

@Deprecated

**public int** getInstrumentState_bak() {

**if** (**mInstrumentState** == **null**) **return *STATE_IDLE***;

**return mInstrumentState**.getState();

}

**public static int** getInstrumentState()

{

**if**(*mController* == **null**) **return *INST_STATE_NO_DEV***;

**if**(!*mController*.isConnected())**return *INST_STATE_DISCONNECTED***;

**if**(*mController*.**isConnecting**)**return *INST_STATE_CONNECTING***;

**if**(*mController*.**mInstrumentSN** == **null**) **return *INST_STATE_INITIALIZING***;

**if**(*mController*.**mInstrumentState** == **null**)**return *INST_STATE_IDLE***;

**switch** (*mController*.**mInstrumentState**.getState())

{

**case *STATE_TESTING***:

**return *INST_STATE_TESTING***;

**case *STATE_TEST_DONE***:

**return *INST_STATE_FINISH_TEST***;

**default**:

**return *INST_STATE_IDLE***;

}

}

**public** String getInstrumentSN() {

**if**(**mInstrumentSN** == **null**) **return null**;

**return mInstrumentSN**.getValue();

}

**public static void** addOnConnectionChangedListener(OnConnectionChangedListener listener) {

*mOnConnectionChangedListeners*.add(listener);

}

**public static void** removeOnConnectionChangedListener(OnConnectionChangedListener listener) {

*mOnConnectionChangedListeners*.remove(listener);

}

**public void** setOnStateChangedListener(OnStateChangedListener listener) {

**this**.**mOnStateChangedListener** = listener;

}

**public void** setOnBatteryStateChangedListener(OnBatteryStateChangedListener listener) {

**this**.**mOnBatteryStateChangedListener** = listener;

}

**public void** setOnDataReceivedListener(OnDataReceivedListener listener) {

**this**.**mOnDataReceivedListener** = listener;

}

**private class** OnSpirometerDataReceivedListener **implements** BtDeviceController.OnBtDeviceReceivedListener {

@Override

**public void** onConnectionChanged(**boolean** connected) {

refreshConnectionState(connected);

}

@Override

**public void** onDataReceived(DataFrame frame, String uuid) {

handleDataFrame(frame, uuid);

}

}

**private synchronized void** startTimeoutTimer() {

**if** (**mTimeoutTimer** != **null**) {

**mTimeoutTimer**.cancel();

}

**mTimeoutTimer** = **new** Timer();

**mTimeoutTimer**.schedule(**new** TimerTask() {

@Override

**public void** run() {

Log.*e*(**"PfmmedController"**, **"auto disconnect device"**);

stopTimeoutTimer();

disconnect();

refreshConnectionState(**false**);

}

}, 8000);

}

**private synchronized void** stopTimeoutTimer() {

**if** (**mTimeoutTimer** == **null**) **return**;

**mTimeoutTimer**.cancel();

**mTimeoutTimer** = **null**;

}

**private void** handleDataFrame(DataFrame frame, String uuid) {

DataFrame responseFrame = **null**;

**switch** (frame.getCommand()) {

**case *CMD_INST_INFO***:

responseFrame = handleCmdInstInfo(frame, uuid);

**break**;

**case *CMD_INST_STATE***:

responseFrame = handleCmdInstState(frame, uuid);

**break**;

**case *CMD_CELL_STATE***:

responseFrame = handleCmdBatteryState(frame, uuid);

**break**;

**case *CMD_GET_DATA***:

responseFrame = handleCmdGetDataRecord(frame, uuid);

**break**;

**case *CMD_GET_CURVE***:

responseFrame = handleCmdGetCurve(frame, uuid);

**break**;

**case *CMD_CHECK_SYNC***:

responseFrame = handleCmdCheckSync(frame, uuid);

**break**;

**case *CMD_SET_USERINFO***:

responseFrame = handleCmdSetUserInfo(frame, uuid);

**break**;

**case *CMD_SET_SYSTEM_TIME***:

responseFrame = handleCmdSetSystemTime(frame, uuid);

**break**;

**default**:

responseFrame = handleOtherCmd(frame, uuid);

}

**if** (responseFrame != **null** && **mService** != **null**) {

BtDeviceController controller = **mService**.getController();

**if** (controller != **null**)

**mService**.getController().writeSendDataFrame(responseFrame);

}

}

**private** DataFrame handleCmdInstInfo(DataFrame frame, String uuid) {

InstrumentInfoServlet servlet = **new** InstrumentInfoServlet(***CMD_INST_INFO***);

DataFrame responseFrame = servlet.handlePost(frame, uuid);

BTInstrumentInfo instrumentInfo = servlet.getValue();

**if** (instrumentInfo != **null**

&& instrumentInfo.getType() == BTInstrumentInfo.***IN_INST_SN***) {

**mInstrumentSN** = servlet.getValue();

stopTimeoutTimer();

}

refreshInstrumentInfo(servlet.getValue());

**return** responseFrame;

}

**private** DataFrame handleCmdInstState(DataFrame frame, String uuid) {

InstrumentStateServlet servlet = **new** InstrumentStateServlet(***CMD_INST_STATE***);

DataFrame responseFrame = servlet.handlePost(frame, uuid);

**mInstrumentState** = servlet.getValue();

refreshInstrumentState(servlet.getValue());

**if** (**mInstrumentState**.getState() == ***STATE_SHUT_BLE***) {

disconnect();

}

**return** responseFrame;

}

**private** DataFrame handleCmdBatteryState(DataFrame frame, String uuid) {

BatteryStateServlet servlet = **new** BatteryStateServlet(***CMD_CELL_STATE***);

DataFrame responseFrame = servlet.handlePost(frame, uuid);

**mBatteryState** = servlet.getValue();

refreshBatteryStateChanged(**mBatteryState**);

**return** responseFrame;

}

**private** DataFrame handleCmdGetDataRecord(DataFrame frame, String uuid) {

DataRecordServlet servlet = **new** DataRecordServlet(***CMD_GET_DATA***);

DataFrame responseFrame = servlet.handlePost(frame, uuid);

BTSpirometerDataRecord record = servlet.getValue();

**if** (**mInstrumentSN** != **null**) record.setDeviceSN(**mInstrumentSN**.getValue());

refreshDataRecord(record);

**return** responseFrame;

}

**private** CurveServlet **mBufferedCurveSevlet** = **null**;

**private** DataFrame handleCmdGetCurve(DataFrame frame, String uuid) {

CurveServlet servlet;

**if** (**mBufferedCurveSevlet** == **null**)

servlet = **new** CurveServlet(***CMD_GET_CURVE***);

**else**

servlet = **mBufferedCurveSevlet**;

DataFrame responseFrame = servlet.handlePost(frame, uuid);

BTCurveRecord curve = servlet.getValue();

**if** (curve == **null**) {

**mBufferedCurveSevlet** = servlet;

} **else** {

**if** (**mInstrumentSN** != **null**) curve.setDeviceSN(**mInstrumentSN**.getValue());

**if** (curve.getType() == BTCurveRecord.***CURVE_V_T***) {

**mBufferedCurveSevlet** = **null**;

}

refreshCurveRecord(curve);

}

**return** responseFrame;

}

**private** DataFrame handleCmdCheckSync(DataFrame frame, String uuid) {

SynchronizeStateServlet servlet = **new** SynchronizeStateServlet(***CMD_CHECK_SYNC***, 0);

DataFrame responseFrame = servlet.handlePost(frame, uuid);

**return** responseFrame;

}

**private** DataFrame handleCmdSetUserInfo(DataFrame frame, String uuid) {

PatientInfo currentPatient = ClientManager.*getInstance*().getCurrentPatient();

**if**(currentPatient != **null**)

{

**mCurrentHealthInfo**.setBirth(currentPatient.getBirth());

**mCurrentHealthInfo**.setSex(currentPatient.getSex());

**mCurrentHealthInfo**.setHeight(currentPatient.getHeight());

**mCurrentHealthInfo**.setWeight(currentPatient.getWeight());

}

SetUserInfoServlet servlet = **new** SetUserInfoServlet(***CMD_SET_USERINFO***, **mCurrentHealthInfo**);

DataFrame responseFrame = servlet.handlePost(frame, uuid);

**return** responseFrame;

}

**private** DataFrame handleCmdSetSystemTime(DataFrame frame, String uuid) {

SetSystemTimeServlet servlet = **new** SetSystemTimeServlet(***CMD_SET_SYSTEM_TIME***);

DataFrame responseFrame = servlet.handlePost(frame, uuid);

**return** responseFrame;

}

**private** DataFrame handleOtherCmd(DataFrame frame, String uuid) {

OtherCmdServlet servlet = **new** OtherCmdServlet(frame.getCommand());

**return** servlet.handlePost(frame, uuid);

}

**private void** forceUnbind() {

}

**private synchronized void** refreshConnectionState(**boolean** connection) {

**synchronized** (***TAG***) {

**if** (!connection && **isBound**) {

**mContext**.unbindService(**mServiceConnection**);

**isBound** = **false**;

*//Log.e(TAG, "unbind service");*

}

}

**isConnecting** = **false**;

**if** (connection) {

**for** (**int** i = 0; i < *mOnConnectionChangedListeners*.size(); i++) {

*mOnConnectionChangedListeners*.get(i).onConnected();

}

} **else** {

**for** (**int** i = 0; i < *mOnConnectionChangedListeners*.size(); i++) {

*mOnConnectionChangedListeners*.get(i).onDisconnected();

}

}

}

**private void** refreshInstrumentState(BTInstrumentState state) {

**if** (**mOnStateChangedListener** != **null**) {

**mOnStateChangedListener**.onStateChanged(state);

}

}

**private void** refreshBatteryStateChanged(BTBatteryState state) {

**if** (**mOnBatteryStateChangedListener** != **null**) {

**mOnBatteryStateChangedListener**.onBatteryStateChanged(state);

}

}

**private void** refreshInstrumentInfo(BTInstrumentInfo instInfo) {

**if** (**mOnDataReceivedListener** != **null**) {

**mOnDataReceivedListener**.onInstrumentInfoReceived(instInfo);

}

}

**private void** refreshDataRecord(BTSpirometerDataRecord record) {

**if** (**mOnDataReceivedListener** != **null**) {

**mOnDataReceivedListener**.onDataRecordReceived(record);

}

}

**private void** refreshCurveRecord(BTCurveRecord curve) {

**if** (**mOnDataReceivedListener** != **null**) {

**switch** (curve.getType()) {

**case** BTCurveRecord.***CURVE_F_T***:

**mOnDataReceivedListener**.onFTCurveReceived(curve);

**break**;

**case** BTCurveRecord.***CURVE_F_V***:

**mOnDataReceivedListener**.onFVCurveReceived(curve);

**break**;

**case** BTCurveRecord.***CURVE_V_T***:

**mOnDataReceivedListener**.onVTCurveReceived(curve);

**break**;

}

}

}

**public interface** OnConnectionChangedListener {

**public void** onConnected();

**public void** onDisconnected();

}

**public interface** OnStateChangedListener {

**public void** onStateChanged(BTInstrumentState state);

}

**public interface** OnBatteryStateChangedListener {

**public void** onBatteryStateChanged(BTBatteryState state);

}

**public interface** OnDataReceivedListener {

**public void** onInstrumentInfoReceived(BTInstrumentInfo instInfo);

**public void** onDataRecordReceived(BTSpirometerDataRecord record);

**public void** onFTCurveReceived(BTCurveRecord curve);

**public void** onFVCurveReceived(BTCurveRecord curve);

**public void** onVTCurveReceived(BTCurveRecord curve);

}

}

**package** com.elinkcare.ubreath.desensitization.core.data;

*/***

** Created by ${Ray} on 2017/3/29.*

**/*

**public class** ChildAccountInfo {

**private** String **id**;

**private** String **userName**;

**private** String **realName**;

**private** String **phone**;

**private** String **password**;

**private long createTime**;

**private** String **hospitalName**;

**private** String **departmentName**;

**private int userPermission**;

**public** String getId() {

**return id**;

}

**public void** setId(String id) {

**this**.**id** = id;

}

**public** String getRealName() {

**return realName**;

}

**public void** setRealName(String realName) {

**this**.**realName** = realName;

}

**public** String getPhone() {

**return phone**;

}

**public void** setPhone(String phone) {

**this**.**phone** = phone;

}

**public** String getPassword() {

**return password**;

}

**public void** setPassword(String password) {

**this**.**password** = password;

}

**public long** getCreateTime() {

**return createTime**;

}

**public void** setCreateTime(**long** createTime) {

**this**.**createTime** = createTime;

}

**public** String getUserName() {

**return userName**;

}

**public void** setUserName(String userName) {

**this**.**userName** = userName;

}

**public int** getUserPermission() {

**return userPermission**;

}

**public void** setUserPermission(**int** userPermission) {

**this**.**userPermission** = userPermission;

}

**public** String getHospitalName() {

**return hospitalName**;

}

**public void** setHospitalName(String hospitalName) {

**this**.**hospitalName** = hospitalName;

}

**public** String getDepartmentName() {

**return departmentName**;

}

**public void** setDepartmentName(String departmentName) {

**this**.**departmentName** = departmentName;

}

}

**package** com.elinkcare.ubreath.desensitization.core.data;

*/***

** Created by Jason on 16/9/18.*

**/*

**public class** CurveRecordInfo {

**private int**[] **values**;

**public void** setValues(**int**[] values)

{

**this**.**values** = values;

}

**public int**[] getValues()

{

**return this**.**values**;

}

}

**package** com.elinkcare.ubreath.desensitization.core.data;

**import** java.util.Calendar;

*/***

** Created by Jason on 17/2/16.*

**/*

**public class** DesensitizationRecordInfo {

**private** String **id**;

**private long tmpId**;

**private** String **patientId**;

**private int weekNumber**;

**private long injectDate**;

**private int injectNumber**;

**private** String **healthStatus**;

**private int beforePEF**;

**private long beforePEFTime**;

**private int afterPEF**;

**private long afterPEFTime**;

**private int bottleNumber**;

**private int concentration**;

**private int injectDoseTotal**;

**private int injectDose2**;

**private** String **injectPart1**;

**private long injectTime1**;

**private** String **injectPart2**;

**private long injectTime2**;

**private boolean injectSeparate**;

**private** String **immedWhealSize**;

**private long immedReactionTime**;

**private** String **immedReactionHandling**;

**private** String **immedReaction**;

**private** String **delayWhealSize**;

**private long delayReactionTime**;

**private** String **delayReactionHandling**;

**private** String **delayReaction**;

**private boolean commited** = **false**;

**public** DesensitizationRecordInfo(String id)

{

**this**.**id** = id;

}

**public** String getId() {

**return id**;

}

**public void** setId(String id) {

**this**.**id** = id;

}

**public long** getTempId()

{

**return tmpId**;

}

**public void** setTempId(**long** tmpId)

{

**this**.**tmpId** = tmpId;

}

**public** String getPatientId() {

**return patientId**;

}

**public void** setPatientId(String patientId) {

**this**.**patientId** = patientId;

}

**public int** getWeekNumber() {

**return weekNumber**;

}

**public void** setWeekNumber(**int** weekNumber) {

**this**.**weekNumber** = weekNumber;

}

**public long** getInjectDate() {

**return injectDate**;

}

**public void** setInjectDate(**long** injectDate) {

**this**.**injectDate** = injectDate;

}

**public int** getInjectNumber() {

**return injectNumber**;

}

**public void** setInjectNumber(**int** injectNumber) {

**this**.**injectNumber** = injectNumber;

}

**public** String getHealthStatus() {

**return healthStatus** == **null** ? **""** : **healthStatus**;

}

**public void** setHealthStatus(String healthStatus) {

**this**.**healthStatus** = healthStatus;

}

**public int** getBeforePEF() {

**return beforePEF**;

}

**public void** setBeforePEF(**int** beforePEF) {

**this**.**beforePEF** = beforePEF;

}

**public long** getBeforePEFTime() {

**return beforePEFTime**;

}

**public void** setBeforePEFTime(**long** beforePEFTime) {

**this**.**beforePEFTime** = beforePEFTime;

}

**public int** getAfterPEF() {

**return afterPEF**;

}

**public void** setAfterPEF(**int** afterPEF) {

**this**.**afterPEF** = afterPEF;

}

**public long** getAfterPEFTime() {

**return afterPEFTime**;

}

**public void** setAfterPEFTime(**long** afterPEFTime) {

**this**.**afterPEFTime** = afterPEFTime;

}

**public int** getBottleNumber() {

**return bottleNumber**;

}

**public void** setBottleNumber(**int** bottleNumber) {

**this**.**bottleNumber** = bottleNumber;

}

**public int** getConcentration() {

**return concentration**;

}

**public void** setConcentration(**int** concentration) {

**this**.**concentration** = concentration;

}

**public** String getInjectDose() {

**if**(isInjectSeparate())

{

**return** String.*format*(**"%1.1f/%1.1f"**, 0.01f * (**injectDoseTotal** - **injectDose2**),

0.01f * **injectDose2**);

}

**else**

{

**return** String.*format*(**"%1.1f"**, 0.01f * **injectDoseTotal**);

}

}

**public void** setInjectDoseTotal(**int** dose)

{

**this**.**injectDoseTotal** = dose;

}

**public void** setInjectDose2(**int** dose2)

{

**this**.**injectDose2** = dose2;

}

**public int** getInjectDoseTotal()

{

**return injectDoseTotal**;

}

**public int** getInjectDose2()

{

**return injectDose2**;

}

**public** String getInjectPart1() {

**return injectPart1** == **null** ? **""** : **injectPart1**;

}

**public void** setInjectPart1(String injectPart1) {

**this**.**injectPart1** = injectPart1;

}

**public long** getInjectTime1() {

**return injectTime1**;

}

**public void** setInjectTime1(**long** injectTime1) {

**this**.**injectTime1** = injectTime1;

}

**public** String getInjectPart2() {

**return injectPart2** == **null** ? **""** : **injectPart2**;

}

**public void** setInjectPart2(String injectPart2) {

**this**.**injectPart2** = injectPart2;

}

**public long** getInjectTime2() {

**return injectTime2**;

}

**public void** setInjectTime2(**long** injectTime2) {

**this**.**injectTime2** = injectTime2;

}

**public boolean** isInjectSeparate() {

**return injectSeparate**;

}

**public void** setInjectSeparate(**boolean** injectSeparate) {

**this**.**injectSeparate** = injectSeparate;

}

**public** String getImmedWhealSize() {

**return immedWhealSize** == **null** ? **""** : **immedWhealSize**;

}

**public void** setImmedWhealSize(String immedWhealSize) {

**this**.**immedWhealSize** = immedWhealSize;

}

**public long** getImmedReactionTime() {

**return immedReactionTime**;

}

**public void** setImmedReactionTime(**long** immedReactionTime) {

**this**.**immedReactionTime** = immedReactionTime;

}

**public** String getImmedReactionHandling() {

**return immedReactionHandling** == **null** ? **""** : **immedReactionHandling**;

}

**public void** setImmedReactionHandling(String immedReactionHandling) {

**this**.**immedReactionHandling** = immedReactionHandling;

}

**public** String getImmedReaction() {

**return immedReaction** == **null** ? **""** : **immedReaction**;

}

**public void** setImmedReaction(String immedReaction) {

**this**.**immedReaction** = immedReaction;

}

**public** String getDelayWhealSize() {

**return delayWhealSize** == **null** ? **""** : **delayWhealSize**;

}

**public void** setDelayWhealSize(String delayWhealSize) {

**this**.**delayWhealSize** = delayWhealSize;

}

**public long** getDelayReactionTime() {

**return delayReactionTime**;

}

**public void** setDelayReactionTime(**long** delayReactionTime) {

**this**.**delayReactionTime** = delayReactionTime;

}

**public** String getDelayReactionHandling() {

**return delayReactionHandling** == **null** ? **""** : **delayReactionHandling**;

}

**public void** setDelayReactionHandling(String delayReactionHandling) {

**this**.**delayReactionHandling** = delayReactionHandling;

}

**public** String getDelayReaction() {

**return delayReaction** == **null** ? **""** : **delayReaction**;

}

**public void** setDelayReaction(String delayReaction) {

**this**.**delayReaction** = delayReaction;

}

**public boolean** isCommited() {

**return commited**;

}

**public void** setCommited(**boolean** commited) {

**this**.**commited** = commited;

}

**public boolean** isCurrentRecord()

{

**long** todayWeekStart = *getWeekStart*(System.*currentTimeMillis*());

**long** recordWeekStart = *getWeekStart*(getInjectDate() * 1000);

**return** todayWeekStart == recordWeekStart;

}

**public boolean** isWithImmedReaction()

{

**if**(getImmedReaction().length() > 0)**return true**;

**if**(getImmedReactionHandling().length() > 0) **return true**;

**if**(getImmedWhealSize().length() > 0) **return true**;

**return false**;

}

**public boolean** isWithDelayReaction()

{

**if**(getDelayReaction().length() > 0) **return true**;

**if**(getDelayReactionHandling().length() > 0) **return true**;

**if**(getDelayWhealSize().length() > 0) **return true**;

**return false**;

}

**public** DesensitizationRecordInfo copy()

{

DesensitizationRecordInfo newRecord = **new** DesensitizationRecordInfo(**id**);

newRecord.**patientId** = **patientId**;

newRecord.**tmpId** = **tmpId**;

newRecord.**weekNumber** = **weekNumber**;

newRecord.**injectDate** = **injectDate**;

newRecord.**injectNumber** = **injectNumber**;

newRecord.**healthStatus** = **healthStatus**;

newRecord.**beforePEF** = **beforePEF**;

newRecord.**beforePEFTime** = **beforePEFTime**;

newRecord.**afterPEF** = **afterPEF**;

newRecord.**afterPEFTime** = **afterPEFTime**;

newRecord.**injectSeparate** = **injectSeparate**;

newRecord.**injectDoseTotal** = **injectDoseTotal**;

newRecord.**injectDose2** = **injectDose2**;

newRecord.**bottleNumber** = **bottleNumber**;

newRecord.**injectPart1** = **injectPart1**;

newRecord.**injectPart2** = **injectPart2**;

newRecord.**injectTime1** = **injectTime1**;

newRecord.**injectTime2** = **injectTime2**;

newRecord.**immedWhealSize** = **immedWhealSize**;

newRecord.**immedReaction** = **immedReaction**;

newRecord.**immedReactionHandling** = **immedReactionHandling**;

newRecord.**immedReactionTime** = **immedReactionTime**;

newRecord.**delayWhealSize** = **delayWhealSize**;

newRecord.**delayReaction** = **delayReaction**;

newRecord.**delayReactionHandling** = **delayReactionHandling**;

newRecord.**delayReactionTime** = **delayReactionTime**;

**return** newRecord;

}

**public static** DesensitizationRecordInfo create(DesensitizationRecordInfo oldRecord)

{

DesensitizationRecordInfo newRecord = **new** DesensitizationRecordInfo(**null**);

Calendar cal = Calendar.*getInstance*();

cal.set(Calendar.***HOUR_OF_DAY***, 0);

cal.set(Calendar.***MINUTE***, 0);

cal.set(Calendar.***MINUTE***, 0);

**long** injectDate = cal.getTimeInMillis() / 1000;

**int** weekSkip = (**int**) ((*getWeekStart*(injectDate * 1000) - *getWeekStart*(oldRecord.getInjectDate() * 1000))

/ (7 * 24 * 3600 * 1000));

**if**(weekSkip <= 0) weekSkip = 1;

newRecord.setWeekNumber(oldRecord.getWeekNumber() + weekSkip);

newRecord.setInjectDate(injectDate);

newRecord.setInjectNumber(oldRecord.getInjectNumber() + 1);

newRecord.setHealthStatus(**"好"**);

newRecord.setBottleNumber(*getAutoBottleNumber*(newRecord.getInjectNumber()));

newRecord.setInjectDoseTotal(*getAutoInjectDose*(newRecord.getInjectNumber()));

**if**(**"右"**.equals(oldRecord.getInjectPart1())) {

newRecord.setInjectPart1(**"左"**);

}

**else**

{

newRecord.setInjectPart1(**"右"**);

}

newRecord.setInjectTime1(System.*currentTimeMillis*() / 1000);

newRecord.setInjectSeparate(**false**);

newRecord.setInjectPart2(**null**);

newRecord.setInjectTime2(0);

newRecord.setImmedReactionTime(System.*currentTimeMillis*() / 1000);

**return** newRecord;

}

**public static** DesensitizationRecordInfo create(**int** weekNumber)

{

DesensitizationRecordInfo newRecord = **new** DesensitizationRecordInfo(**null**);

Calendar cal = Calendar.*getInstance*();

cal.set(Calendar.***HOUR_OF_DAY***, 0);

cal.set(Calendar.***MINUTE***, 0);

cal.set(Calendar.***MINUTE***, 0);

**long** injectDate = cal.getTimeInMillis() / 1000;

newRecord.setWeekNumber(weekNumber);

newRecord.setInjectDate(injectDate);

newRecord.setInjectNumber(weekNumber);

newRecord.setHealthStatus(**"好"**);

newRecord.setBottleNumber(*getAutoBottleNumber*(newRecord.getInjectNumber()));

newRecord.setInjectDoseTotal(*getAutoInjectDose*(newRecord.getInjectNumber()));

newRecord.setInjectPart1(**"左"**);

newRecord.setInjectTime1(System.*currentTimeMillis*() / 1000);

newRecord.setInjectSeparate(**false**);

newRecord.setInjectPart2(**null**);

newRecord.setInjectTime2(0);

newRecord.setImmedReactionTime(System.*currentTimeMillis*() / 1000);

**return** newRecord;

}

**public static int** getAutoBottleNumber(**int** weekNumber)

{

**int** bottleNumber = 0;

**switch** (weekNumber)

{

**case** 1:

bottleNumber = 1;

**break**;

**case** 2:

bottleNumber = 1;

**break**;

**case** 3:

bottleNumber = 1;

**break**;

**case** 4:

bottleNumber = 2;

**break**;

**case** 5:

bottleNumber = 2;

**break**;

**case** 6:

bottleNumber = 2;

**break**;

**case** 7:

bottleNumber = 3;

**break**;

**case** 8:

bottleNumber = 3;

**break**;

**case** 9:

bottleNumber = 3;

**break**;

**case** 10:

bottleNumber = 4;

**break**;

**case** 11:

bottleNumber = 4;

**break**;

**case** 12:

bottleNumber = 4;

**break**;

**case** 13:

bottleNumber = 4;

**break**;

**case** 14:

bottleNumber = 4;

**break**;

**case** 15:

bottleNumber = 4;

**break**;

}

**if**(weekNumber > 15)

{

bottleNumber = 4;

}

**return** bottleNumber;

}

**public static int** getAutoInjectDose(**int** weekNumber)

{

**int** injectDose = 0;

**switch** (weekNumber)

{

**case** 1:

injectDose = 20;

**break**;

**case** 2:

injectDose = 40;

**break**;

**case** 3:

injectDose = 80;

**break**;

**case** 4:

injectDose = 20;

**break**;

**case** 5:

injectDose = 40;

**break**;

**case** 6:

injectDose = 80;

**break**;

**case** 7:

injectDose = 20;

**break**;

**case** 8:

injectDose = 40;

**break**;

**case** 9:

injectDose = 80;

**break**;

**case** 10:

injectDose = 10;

**break**;

**case** 11:

injectDose = 20;

**break**;

**case** 12:

injectDose = 40;

**break**;

**case** 13:

injectDose = 60;

**break**;

**case** 14:

injectDose = 80;

**break**;

**case** 15:

injectDose = 100;

}

**if**(weekNumber > 15)

{

injectDose = 100;

}

**return** injectDose;

}

**public static long** getWeekStart(**long** day) {

Calendar cal = Calendar.*getInstance*();

cal.setTimeInMillis(day);

**int** dayOfWeek = cal.get(Calendar.***DAY_OF_WEEK***) - cal.getFirstDayOfWeek();

cal.setTimeInMillis(day - 24 * 3600 * 1000 * (dayOfWeek - 1));

cal.set(Calendar.***HOUR_OF_DAY***, 0);

cal.set(Calendar.***MINUTE***, 0);

cal.set(Calendar.***SECOND***, 0);

cal.set(Calendar.***MILLISECOND***, 0);

**return** cal.getTimeInMillis();

}

}

**package** com.elinkcare.ubreath.desensitization.core.data;

**import** java.util.List;

*/***

** Created by ${Ray} on 2017/3/22.*

**/*

**public class** FollowUpInfo {

**private** String **id**;

**private** String **patientId**;

**private** String **followUpId**;

**private long followUpTime**;

**private** String **asthmaSympton**;

**private long asthmaStartTime**;

**private long asthmaEndTime**;

**private** String **rhinitisSympton**;

**private long rhinitisStartTime**;

**private long rhinitisEndTime**;

**private** String **conjunctivitisSympton**;

**private long conjunctivitisStartTime**;

**private long conjunctivitisEndTime**;

**private** String **dermatitisSympton**;

**private long dermatitisStartTime**;

**private long dermatitisEndTime**;

**private** String **respiratoryTractSympton**;

**private long respiratoryTractStartTime**;

**private long respiratoryTractEndTime**;

**private** String **angiocarpySympton**;

**private long angiocarpyStartTime**;

**private long angiocarpyEndTime**;

**private** String **diagestionSystem**;

**private long diagestionSystemStartTime**;

**private long diagestionSystemEndTime**;

**private** String **urinationSystem**;

**private long urinationSystemStartTime**;

**private long urinationSystemEndTime**;

**private** String **nervousSystem**;

**private long nervousStartTime**;

**private long nervousEndTime**;

**private int height**;

**private int weight**;

**private** List<MedicineInfo> **medicineInfos**;*// 用药集合；*

**private int readStatus**;*// 是否查看过； 0 - 没有查看 1- 已查看；*

**private boolean isCheck** = **false**;

**public** FollowUpInfo(String id){

**this**.**id** = id;

}

**public** String getId() {

**return id**;

}

**public void** setId(String id) {

**this**.**id** = id;

}

**public** String getPatientId() {

**return patientId**;

}

**public void** setPatientId(String patientId) {

**this**.**patientId** = patientId;

}

**public** String getFollowUpId() {

**return followUpId**;

}

**public void** setFollowUpId(String followUpId) {

**this**.**followUpId** = followUpId;

}

**public** String getAsthmaSympton() {

**return asthmaSympton**;

}

**public void** setAsthmaSympton(String asthmaSympton) {

**this**.**asthmaSympton** = asthmaSympton;

}

**public long** getFollowUpTime() {

**return followUpTime**;

}

**public void** setFollowUpTime(**long** followUpTime) {

**this**.**followUpTime** = followUpTime;

}

**public long** getAsthmaStartTime() {

**return asthmaStartTime**;

}

**public void** setAsthmaStartTime(**long** asthmaStartTime) {

**this**.**asthmaStartTime** = asthmaStartTime;

}

**public long** getAsthmaEndTime() {

**return asthmaEndTime**;

}

**public void** setAsthmaEndTime(**long** asthmaEndTime) {

**this**.**asthmaEndTime** = asthmaEndTime;

}

**public** String getRhinitisSympton() {

**return rhinitisSympton**;

}

**public void** setRhinitisSympton(String rhinitisSympton) {

**this**.**rhinitisSympton** = rhinitisSympton;

}

**public long** getRhinitisStartTime() {

**return rhinitisStartTime**;

}

**public void** setRhinitisStartTime(**long** rhinitisStartTime) {

**this**.**rhinitisStartTime** = rhinitisStartTime;

}

**public long** getRhinitisEndTime() {

**return rhinitisEndTime**;

}

**public void** setRhinitisEndTime(**long** rhinitisEndTime) {

**this**.**rhinitisEndTime** = rhinitisEndTime;

}

**public** String getConjunctivitisSympton() {

**return conjunctivitisSympton**;

}

**public void** setConjunctivitisSympton(String conjunctivitisSympton) {

**this**.**conjunctivitisSympton** = conjunctivitisSympton;

}

**public long** getConjunctivitisStartTime() {

**return conjunctivitisStartTime**;

}

**public void** setConjunctivitisStartTime(**long** conjunctivitisStartTime) {

**this**.**conjunctivitisStartTime** = conjunctivitisStartTime;

}

**public long** getConjunctivitisEndTime() {

**return conjunctivitisEndTime**;

}

**public void** setConjunctivitisEndTime(**long** conjunctivitisEndTime) {

**this**.**conjunctivitisEndTime** = conjunctivitisEndTime;

}

**public** String getDermatitisSympton() {

**return dermatitisSympton**;

}

**public void** setDermatitisSympton(String dermatitisSympton) {

**this**.**dermatitisSympton** = dermatitisSympton;

}

**public long** getDermatitisStartTime() {

**return dermatitisStartTime**;

}

**public void** setDermatitisStartTime(**long** dermatitisStartTime) {

**this**.**dermatitisStartTime** = dermatitisStartTime;

}

**public long** getDermatitisEndTime() {

**return dermatitisEndTime**;

}

**public void** setDermatitisEndTime(**long** dermatitisEndTime) {

**this**.**dermatitisEndTime** = dermatitisEndTime;

}

**public** String getRespiratoryTractSympton() {

**return respiratoryTractSympton**;

}

**public void** setRespiratoryTractSympton(String respiratoryTractSympton) {

**this**.**respiratoryTractSympton** = respiratoryTractSympton;

}

**public long** getRespiratoryTractStartTime() {

**return respiratoryTractStartTime**;

}

**public void** setRespiratoryTractStartTime(**long** respiratoryTractStartTime) {

**this**.**respiratoryTractStartTime** = respiratoryTractStartTime;

}

**public** String getAngiocarpySympton() {

**return angiocarpySympton**;

}

**public void** setAngiocarpySympton(String angiocarpySympton) {

**this**.**angiocarpySympton** = angiocarpySympton;

}

**public long** getRespiratoryTractEndTime() {

**return respiratoryTractEndTime**;

}

**public void** setRespiratoryTractEndTime(**long** respiratoryTractEndTime) {

**this**.**respiratoryTractEndTime** = respiratoryTractEndTime;

}

**public long** getAngiocarpyStartTime() {

**return angiocarpyStartTime**;

}

**public void** setAngiocarpyStartTime(**long** angiocarpyStartTime) {

**this**.**angiocarpyStartTime** = angiocarpyStartTime;

}

**public long** getAngiocarpyEndTime() {

**return angiocarpyEndTime**;

}

**public void** setAngiocarpyEndTime(**long** angiocarpyEndTime) {

**this**.**angiocarpyEndTime** = angiocarpyEndTime;

}

**public** String getDiagestionSystem() {

**return diagestionSystem**;

}

**public void** setDiagestionSystem(String diagestionSystem) {

**this**.**diagestionSystem** = diagestionSystem;

}

**public long** getDiagestionSystemStartTime() {

**return diagestionSystemStartTime**;

}

**public void** setDiagestionSystemStartTime(**long** diagestionSystemStartTime) {

**this**.**diagestionSystemStartTime** = diagestionSystemStartTime;

}

**public long** getDiagestionSystemEndTime() {

**return diagestionSystemEndTime**;

}

**public void** setDiagestionSystemEndTime(**long** diagestionSystemEndTime) {

**this**.**diagestionSystemEndTime** = diagestionSystemEndTime;

}

**public** String getUrinationSystem() {

**return urinationSystem**;

}
